# Supplementary material for: The human inactive X chromosome modulates expression of the active X chromosome
Source: Cell Genom. 2023 Feb 8;3(2):100259. doi: 10.1016/j.xgen.2023.100259 (PMC9932992; doi:10.1016/j.xgen.2023.100259)
Supplement: Document S1. Figures S1–S19 [file mmc1.pdf]

Cell Genomics, Volume *3*

Supplemental information

The human inactive X chromosome modulates expression of the active X chromosome

Adrianna K. San Roman, Alexander K. Godfrey, Helen Skaletsky, Daniel W. Bellott, Abigail F. Groff, Hannah L. Harris, Laura V. Blanton, Jennifer F. Hughes, Laura Brown, Sidaly Phou, Ashley Buscetta, Paul Kruszka, Nicole Banks, Amalia Dutra, Evgenia Pak, Patricia C. Lasutschinkow, Colleen Keen, Shanlee M. Davis, Nicole R. Tartaglia, Carole Samango-Sprouse, Maximilian Muenke, and David C. Page

Supplemental Information

Figures S1-S19

Figure S1

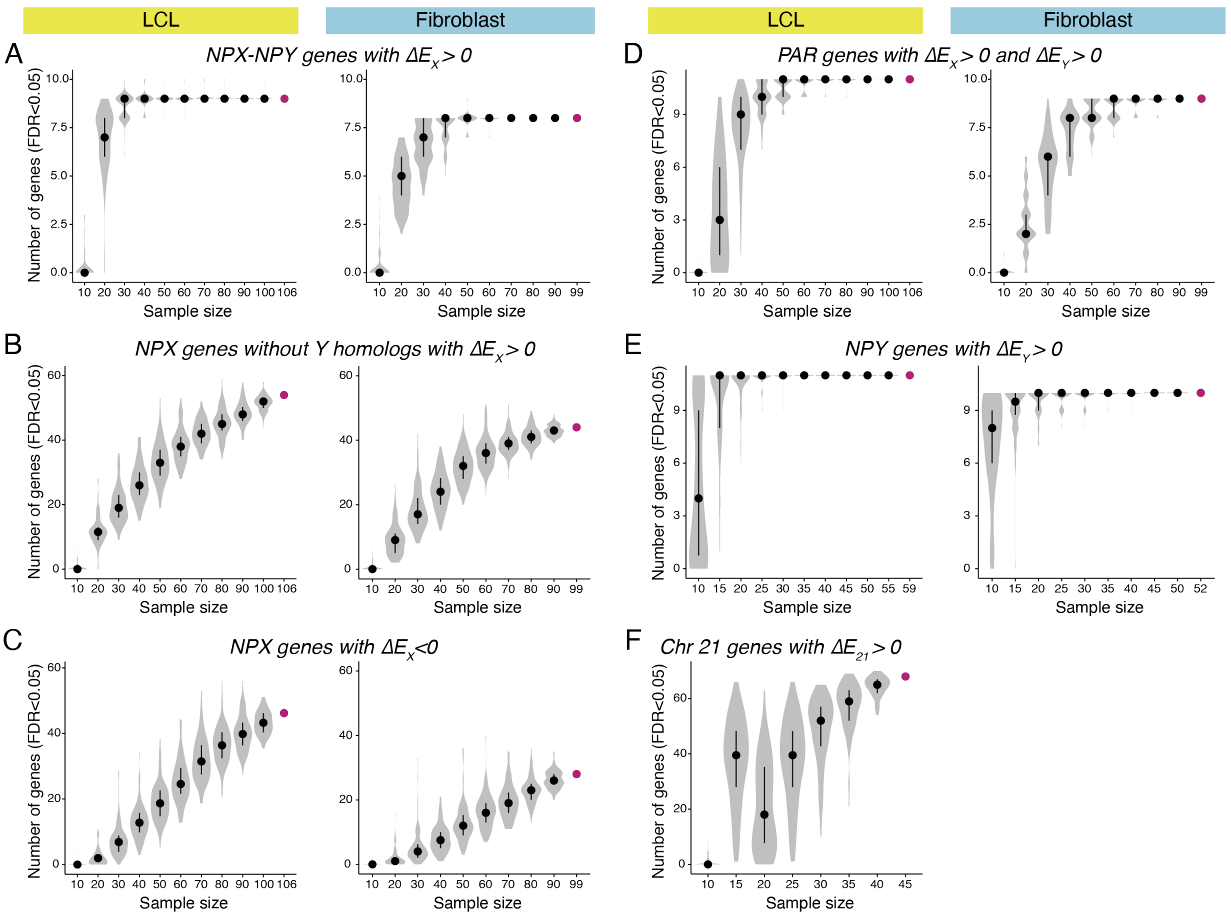

Figure S1. Bootstrapping analyses reveal that few additional significant genes would be observed with a larger sample size, related to Figures 2 and 3. Each analysis was performed by randomly choosing the indicated number of samples and performing the linear regression analysis, repeated 100 times for each sample size. The magenta point in each figure represents the number of significant genes in the final analysis using all of the samples. **(A)** NPX genes with Y homologs with ΔE_X>0 reach saturation by a sample size of 40. (**B)** For NPX genes without Y homologs, the number of genes with ΔE_X>0 increases rapidly at low sample sizes, and then levels off. **(C)** For NPX genes with ΔE_X<0, more samples are required to see an initial increase in the number of significant genes. In LCLs, 50-60 samples are needed to identify 20 genes with ΔE_X<0, whereas only 30-40 samples are needed to identify 20 genes with ΔE_X>0. For LCLs, the maximum increase was observed between 60-70 samples where a median of 7 genes were added, with subsequent increases being less (5 genes from 70-80, 3.5 from 80-90 and 3.5 from 90-100). In fibroblasts, the maximum increase was observed between 40-50 samples where

a median of 4.5 genes were added, with only 3 or 4 additional genes with each additional 10 samples. If this trajectory continues, these results indicate that additional samples would only result in a few additional genes being identified as significant. **(D)** PAR1 genes with both $\Delta E_X>0$ and $\Delta E_Y>0$ reach saturation by sample size of 50. **(E)** NPY genes with $\Delta E_Y>0$ reach saturation after 25 samples containing at least one copy of Chr Y. **(F)** The trajectory for Chr 21 genes with $\Delta E_{21}>0$ increases rapidly between 10-30 samples, and then slows.

Figure S2.

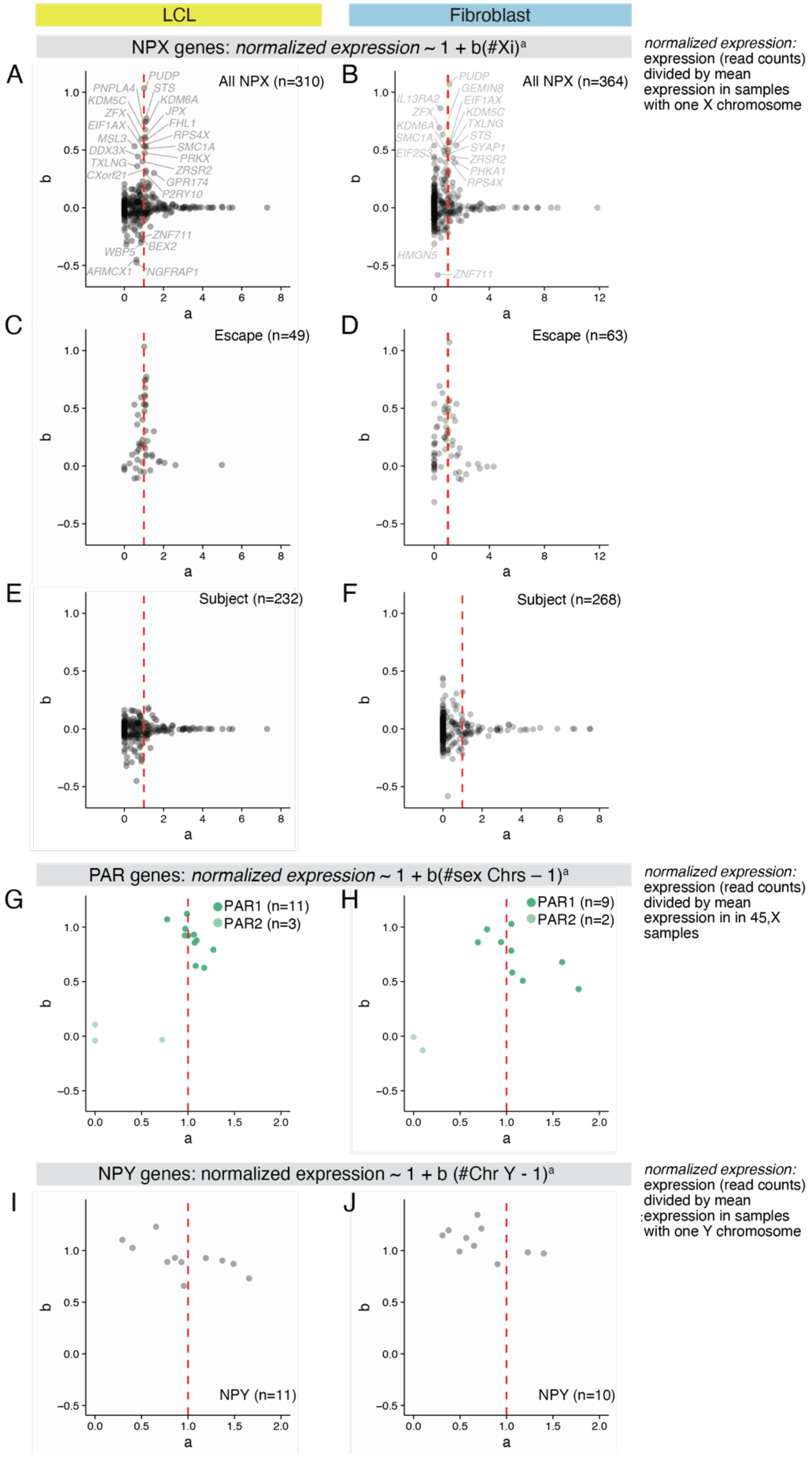

LCL
Fibroblast
NPX genes: *normalized expression* ~ 1 + b(#Xi)[a]
normalized expression: expression (read counts) divided by mean expression in samples with one X chromosome
A
All NPX (n=310)
B
All NPX (n=364)
C
Escape (n=49)
D
Escape (n=63)
E
Subject (n=232)
F
Subject (n=268)
PAR genes: *normalized expression* ~ 1 + b(#sex Chrs – 1)[a]
normalized expression: expression (read counts) divided by mean expression in in 45,X samples
G
PAR1 (n=11)
PAR2 (n=3)
H
PAR1 (n=9)
PAR2 (n=2)
NPY genes: normalized expression ~ 1 + b (#Chr Y - 1)[a]
normalized expression: expression (read counts) divided by mean expression in samples with one Y chromosome
I
NPY (n=11)
J
NPY (n=10)

Figure S2. Linear functions are a good fit for modeling sex chromosome aneuploidy RNA-seq data, related to Figures 2 and 3. For sex chromosome genes in LCLs and fibroblasts, power functions were fit (gray boxes) by non-linear least squares regression. Scatterplots show fitted values for the exponent parameter, *a*, and the coefficient, *b*, for each gene. *XIST* and genes for which the regression model did not converge are excluded from the plots. **(A-B)** Some NPX genes cluster near *a*=1, indicating their expression increases by a fixed amount for each additional X or Y chromosome. These correspond to genes previously annotated as escaping XCI **(C-D)**. Other NPX genes cluster near *a*=0 or *b*=0, indicating that they do not change in expression with additional copies of Xi, and correspond to genes previously annotated as subject to XCI (**E-F**). (**G-H**) PAR1 genes cluster near a=1, indicating their expression increases by a fixed amount for each additional sex chromosome, in contrast to PAR2 genes. (**I-J**) NPY genes also cluster near a=1, indicating their expression increases by a fixed amount for each Y chromosome.

Figure S3.

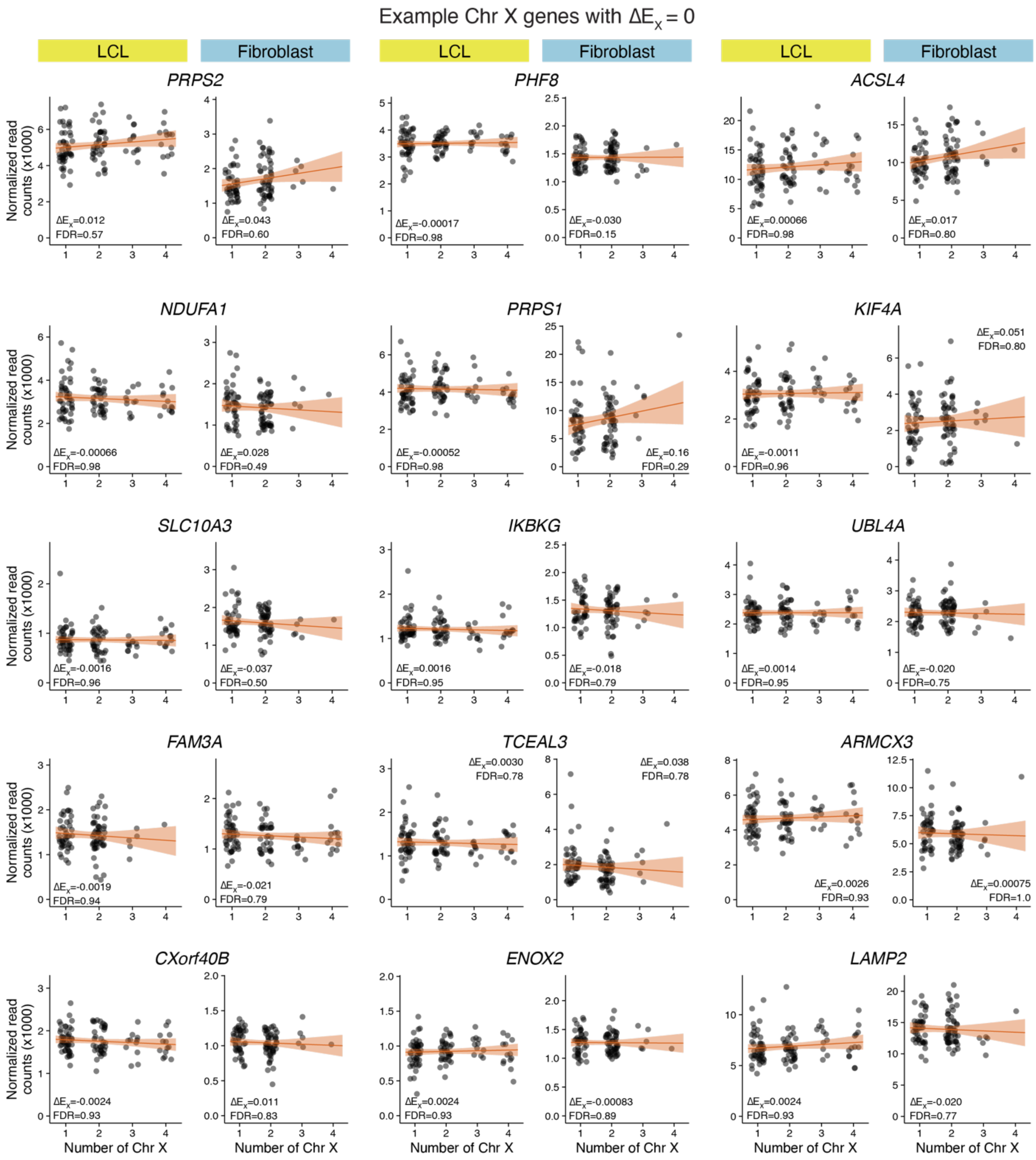

Figure S3. Extended examples of NPX genes with ΔE$_X$=0, related to Figure 2. Scatterplots and regression lines with confidence intervals for fifteen NPX genes with ΔE$_X$=0 in both LCLs and fibroblasts.

Figure S4.

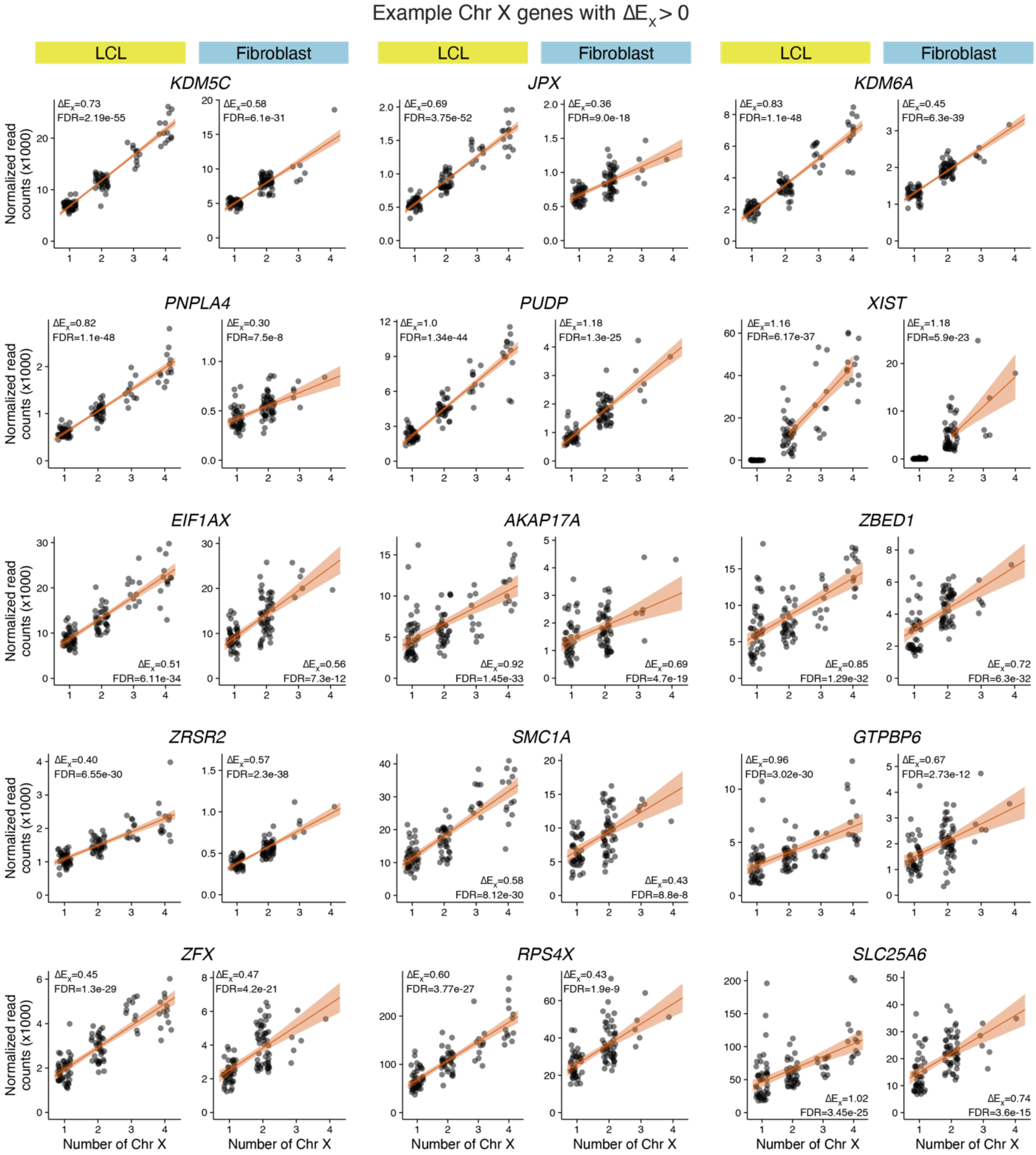

Figure S4. Extended examples of NPX genes with ΔE$_X$>0, related to Figure 2. Scatterplots and regression lines with confidence intervals for fifteen NPX genes with ΔE$_X$>0 in both LCLs and fibroblasts, ordered by increasing FDR in LCLs.

Figure S5.

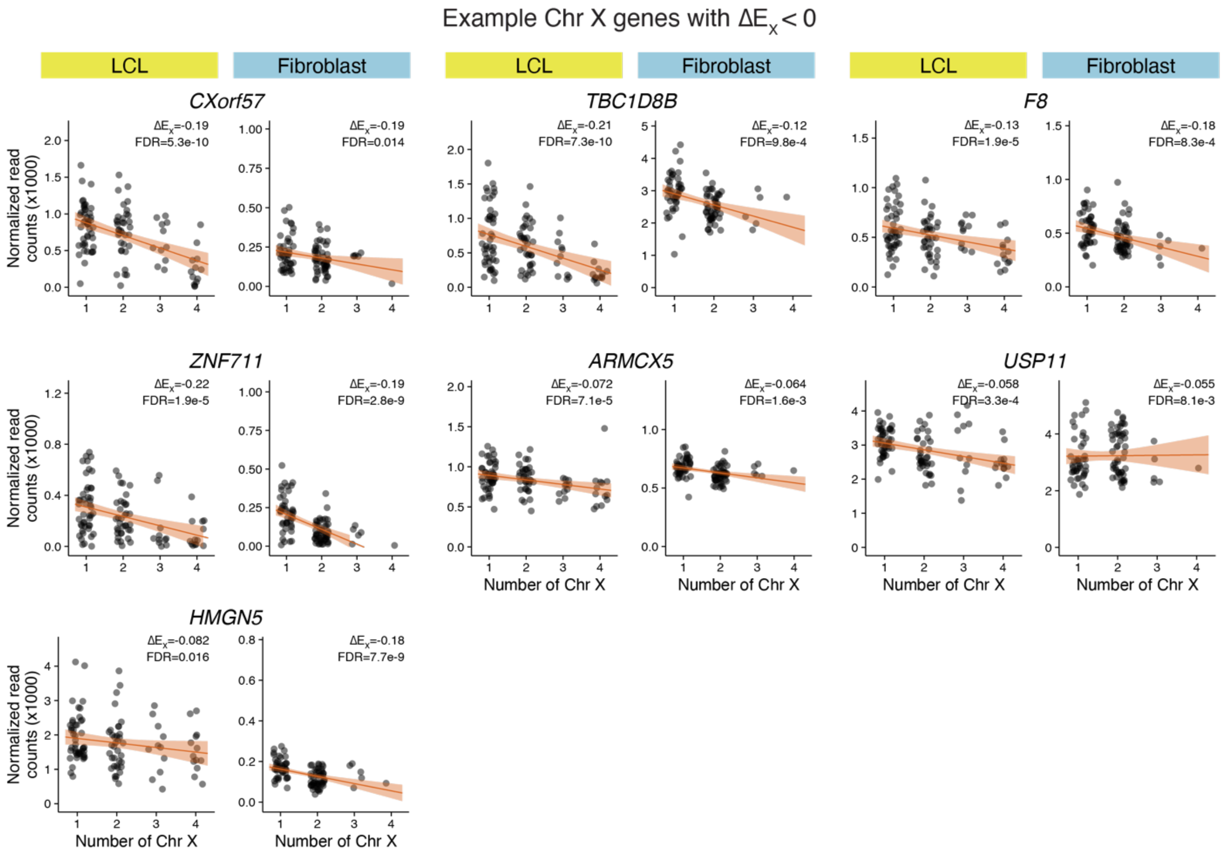

Figure S5. Extended examples of NPX genes with ΔE$_X$<0, related to Figure 2. Scatterplots and regression lines with confidence intervals for seven NPX genes with ΔE$_X$<0 in both LCLs and fibroblasts, ordered by increasing FDR in LCLs.

Figure S6.

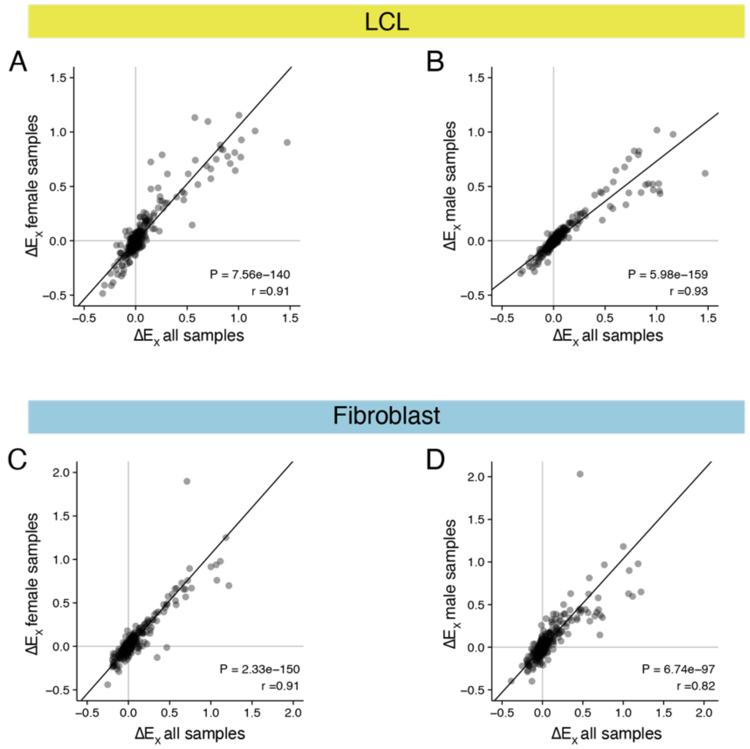

Figure S6. ΔE_X values are similar when calculated with all samples, or with female (0 Y chromosomes) or male (1 Y chromosome) samples, related to Figure 2. Scatterplots of all expressed genes in LCLs (A-B) or fibroblasts (C-D) showing ΔE_X values calculated using all samples (X-axes) or exclusively female samples (A, C Y-axes) or male samples (B, D Y-axes). Deming regression lines, Pearson correlations and P-values are indicated.

Figure S7.

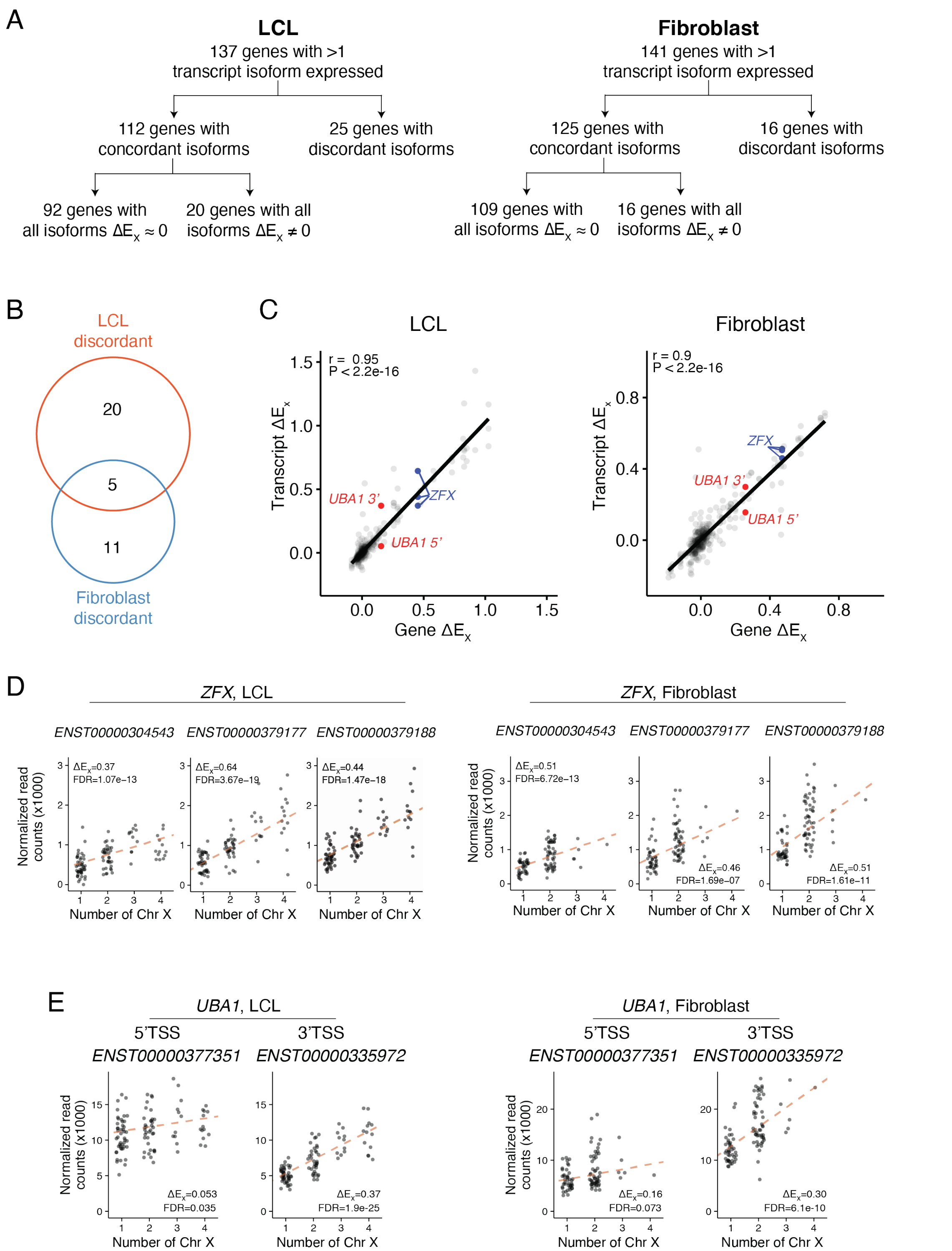

Figure S7. Quantitative analysis of Xi contributions to transcript isoform expression, related to Figure 2. (A) Accounting of Chr X genes with multiple expressed transcript isoforms in LCLs and fibroblasts. Indicated here are the numbers of genes where i) all isoforms show

$\Delta E_X \approx 0$, or ii) all isoforms show ΔE_X values significantly different from zero in the same direction (FDR<0.05), or iii) isoforms show discordant ΔE_X values. For genes with discordant isoforms, two in LCLs (*ARHGEF6, BCOR*) and three in fibroblasts (*SH3KBP1, THOC2, UBA1*) had TSSs >500bp from each other suggesting that differential regulation of the TSS could play a role. **(B)** Venn diagram shows the overlap of genes expressed in both LCL and fibroblasts that had isoforms with discordant ΔE_X values. **(C)** Scatterplots of each transcript isoform's ΔE_X, compared with the gene's ΔE_X in LCLs and fibroblasts. Three *ZFX* isoforms are indicated with blue points; two *UBA1* isoforms indicated with red points. Pearson correlations (r) between isoforms and genes and P-values are indicated. **(D)** Scatterplots of expression of three *ZFX* isoforms across Chr X copy number in LCLs and fibroblasts showing that they consistently have $\Delta E_X > 0$. **(E)** Scatterplots of expression of two *UBA1* isoforms across Chr X copy number in LCLs and fibroblasts showing discordant ΔE_X values. Regression lines, ΔE_X, and FDR are indicated.

Figure S8.

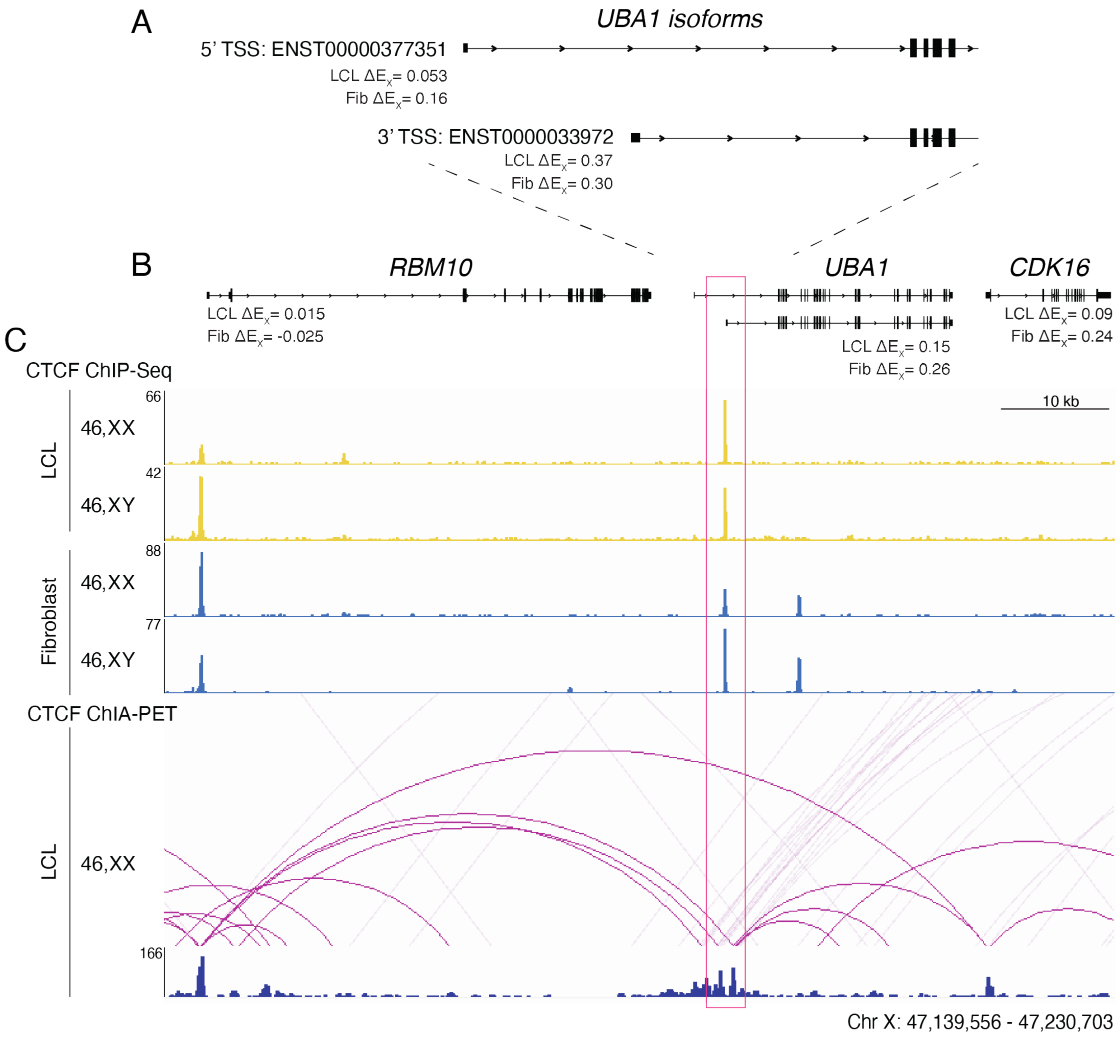

Figure S8. Divergent ΔEX values for transcript isoforms of *UBA1* correlate with domain boundary, related to Figure 2. (A) Transcript models for *UBA1* transcript isoforms. Isoform-level ΔEX values are listed. **(B)** Gene models for *UBA1* and flanking genes *RBM10* and *CDK16.* Gene-level ΔEX values are listed. ΔEX values for these flanking genes are discordant and reflect the ΔEX values of the closest *UBA1* isoform. *UBA1* isoform ΔEX values correlate with those of the flanking genes: the *UBA1* transcript from the 5' start (ENST00000377351) and upstream gene *RBM10* had ΔEX values near zero, while the *UBA1* transcript from the 3' start (ENST0000033972) and downstream gene *CDK16* had significantly positive ΔEX values. **(C)** CTCF ChIP-seq signal tracks (fold-change over background) for two LCL and two fibroblast cell lines show a strong CTCF peak between the two *UBA1* isoform transcription start sites and evidence of divergent three-dimensional interactions, indicating that the two transcription start

sites reside in distinct topologically associated domains along with their neighboring genes. Interaction and signal tracks (fold-change over background) are shown for CTCF ChIA-PET. Red box indicates boundary region. Three previous studies corroborate these results: 1) in human-mouse hybrid cell lines, the 5’ TSS was only active in lines containing Xa, while the 3’ TSS was active in lines containing Xa or Xi [S1]; 2) 3’ TSS transcripts were more abundant in 46,XX than 46,XY cells, while 5’ TSS transcripts showed no such difference [S2] and 3) DNA methylation studies revealed hypermethylation of the 5’ but not the 3’ TSS in XX cells from human, chimp, and horse [S3]. Together, this publicly available data and our own analysis shows that *UBA1* is a unique example of a gene with transcription start sites that are divergently expressed from Xi.

Figure S9.

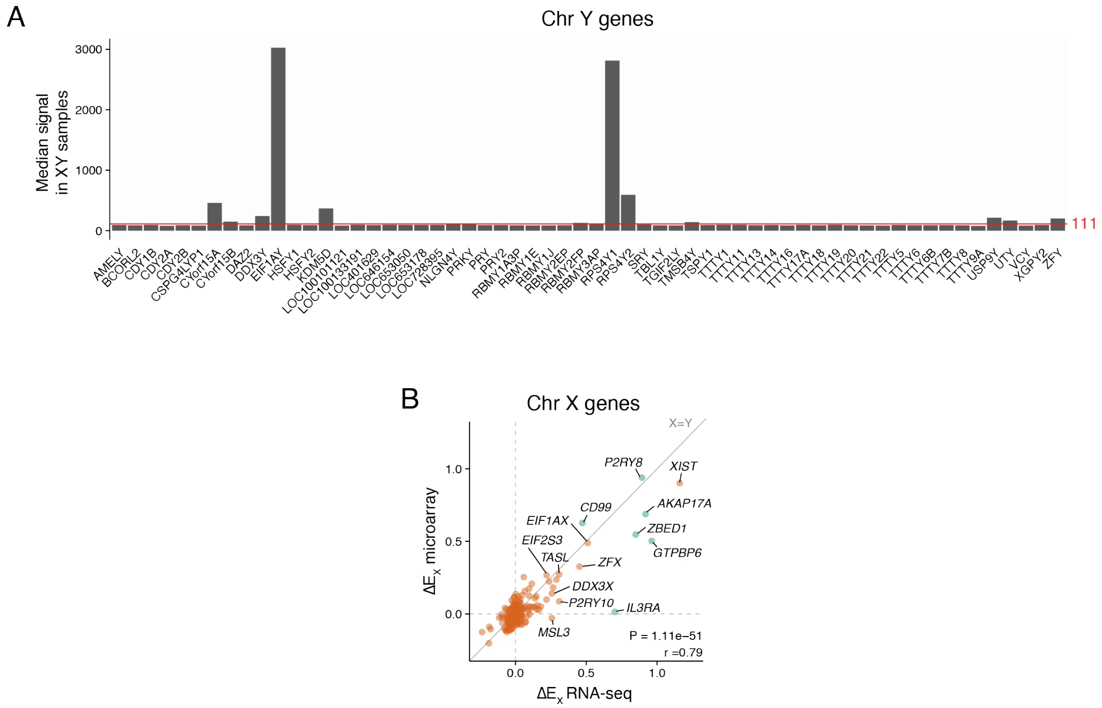

Figure S9. Reanalysis of microarray data from LCLs of individuals with varying sex chromosome constitutions using linear modeling, related to Figure 2. (A) Chr Y gene expression in microarray data from XY samples [S4] was assessed to set a minimum signal threshold of 111 (red line) for expressed genes, based on those expressed in LCL RNA-seq. **(B)** ΔE_X values in RNA-seq and microarray data for expressed Chr X genes were highly correlated. Deming regression line, Pearson correlation and P-value are indicated.

Figure S10.

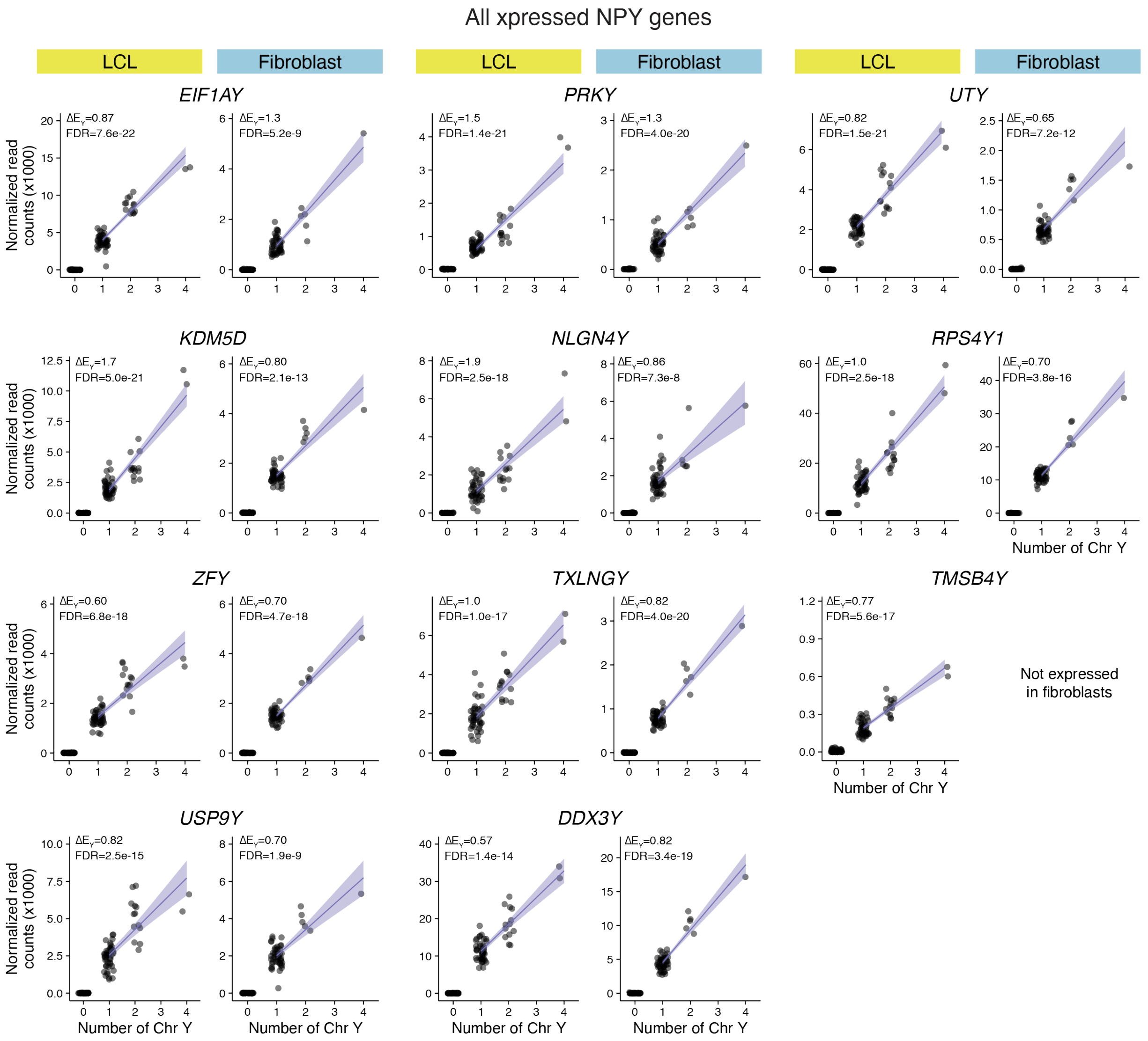

Figure S10. Extended plots of NPY genes, related to Figure 3. Scatterplots and regression lines with confidence intervals for the twelve expressed NPY genes in LCLs or fibroblasts, ordered by increasing FDR in LCLs.

Figure S11.

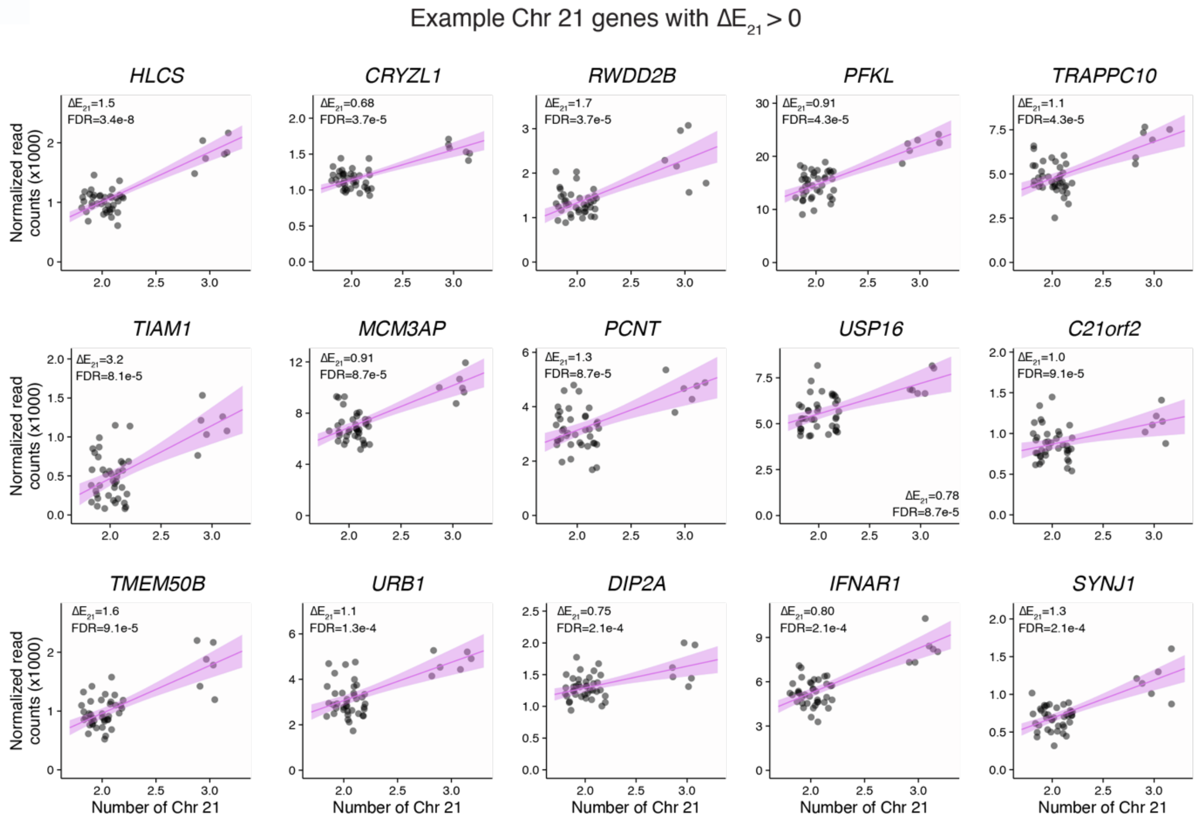

Figure S11. Extended examples of Chr 21 genes with $\Delta E_{21}>0$, related to Figure 3.

Scatterplots and regression lines with confidence intervals for fifteen expressed Chr 21 genes with $\Delta E_{21}>0$ in LCLs, ordered by increasing FDR.

Figure S12.

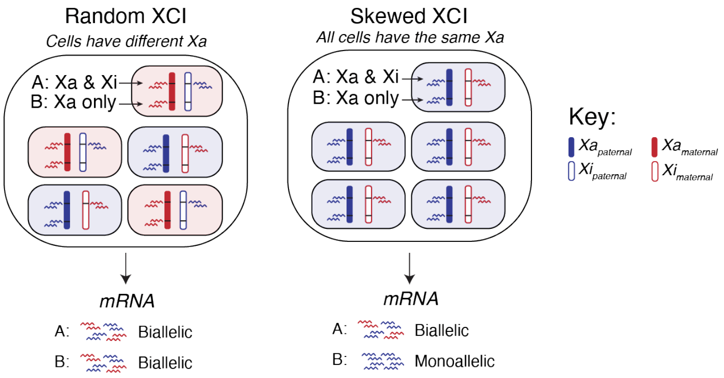

Figure S12. Identifying cell cultures with skewed XCI from RNA-seq data, related to Figure 4. 46,XX individuals typically have a mixture of cells in which either the maternal or paternal X chromosome is active. Because of this, in mRNA extracted from cell cultures with random XCI, all Chr X genes will have biallelic transcripts (*left*). On the other hand, in cell cultures with skewed XCI – where most cells in the culture have the same Xa – genes expressed only from Xa will have monoallelic transcripts, while genes expressed from both Xa and Xi will have biallelic transcripts (*right*).

Figure S13.

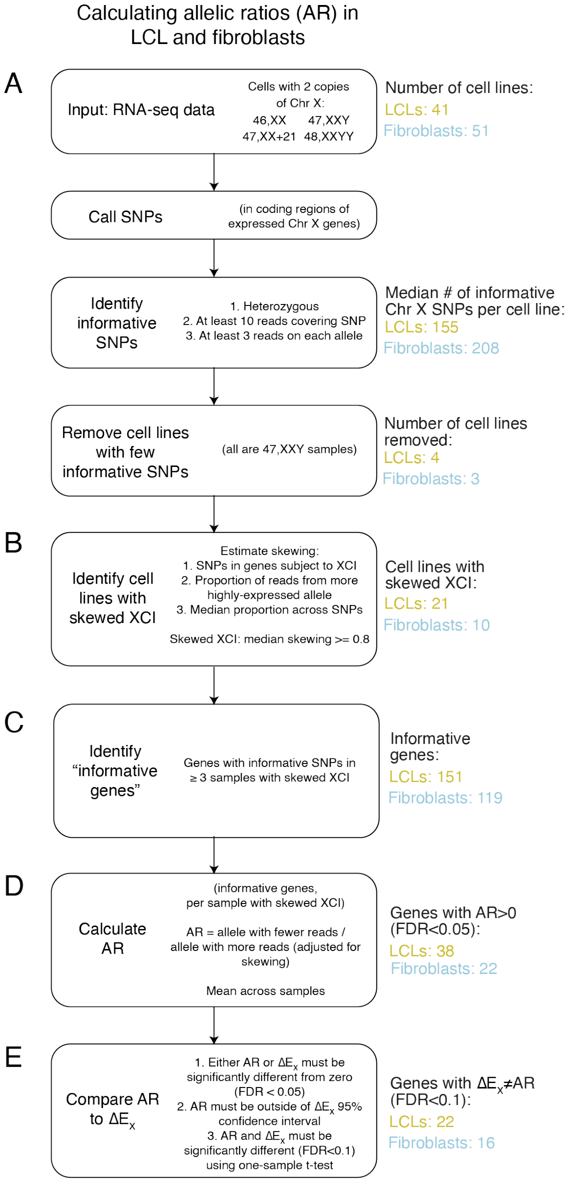

Figure S13. Analytic workflow for identifying cell lines with skewed XCI and calculating allelic ratios from RNA-seq data , related to Figure 4. Each box indicates a step in the analytical pipeline; to the right of each box are summaries of results at each step. **(A)** SNPs were called in samples with two copies of Chr X (46,XX, 46,XX,+21, 47,XXY, 48,XXYY) to identify those that were heterozygous, and could therefore distinguish between two alleles. Of the 41 LCL and 51 fibroblast samples with two copies of Chr X, 7 samples (4 LCLs and 3 fibroblasts; all 47,XXY) were removed because they had few heterozygous SNPs identified on Chr X, indicating that they have two copies of the same Chr X, most likely inherited through a maternal meiotic non-disjunction. **(B)** For samples with sufficient heterozygous Chr X SNPS, the

proportion of reads from the more highly-expressed allele in SNPs from “Xa only” genes (“skewing coefficient”) was calculated. This value can range from 0.5 (equal reads from two alleles) to 1 (all reads are from one allele). Eleven LCL samples and nine fibroblast samples with at least 0.8 median skewing coefficients were considered skewed. Some samples (10 LCL samples and one fibroblast culture) had ≤5 SNPs in “Xa only” genes, but many SNPs in other Chr X genes, indicating that they were “highly skewed.” Together, 21 LCL and 10 fibroblast samples had skewed XCI. **(C)** Following identification of samples with skewed XCI, “informative genes” were classified as those having informative SNPs in at least 3 samples with skewed XCI, resulting in 151 genes in LCLs and 119 in fibroblasts. **(D)** Next, the allelic ratio (AR) was calculated for each SNP by dividing the allele with fewer reads (assumed to represent Xi) by the allele with more reads (assumed to represent Xa), adjusting for the level of skewing in each sample using the median skewing estimate. **(E)** Finally, AR values were compared to ΔE_X values to identify genes that, considering their variance in each metric, were significantly non-equal, and indicated that their expression from Xa was modulated by additional copies of Xi.

Figure S14.

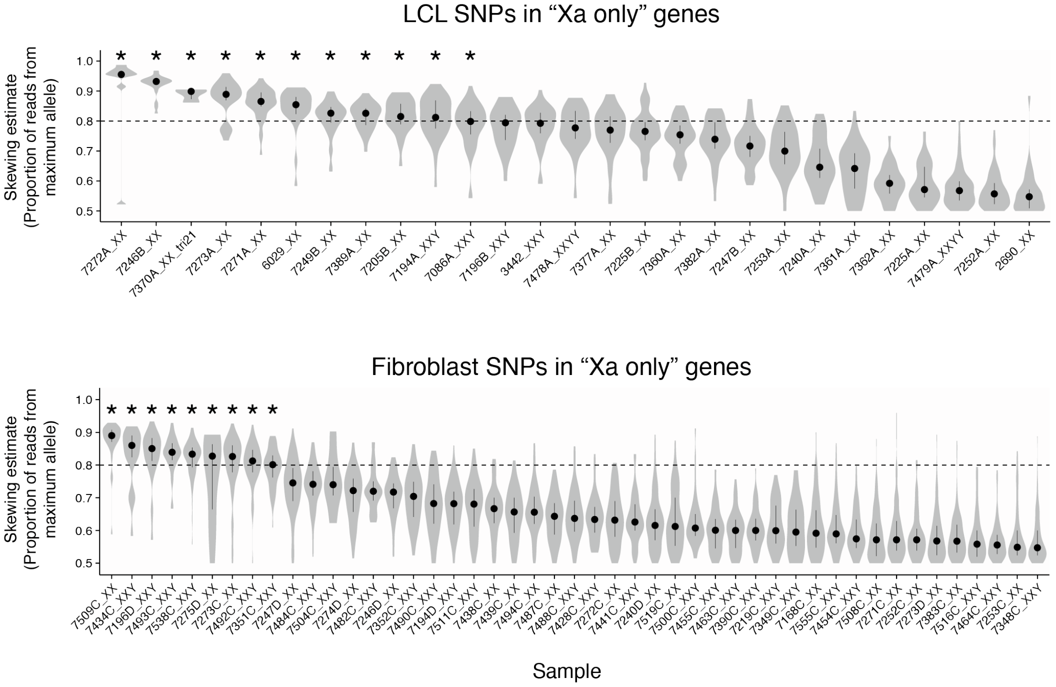

Figure S14. Samples with skewed XCI identified through SNP analysis of Chr X genes expressed from Xa only, related to Figure 4. Samples with >5 heterozygous SNPs in “Xa only” genes were ranked by the median skewing estimate across SNPs in “Xa only” genes. Asterisks, samples with skewing estimates ≥ 0.8 indicating significant skewing of XCI.

Figure S15.

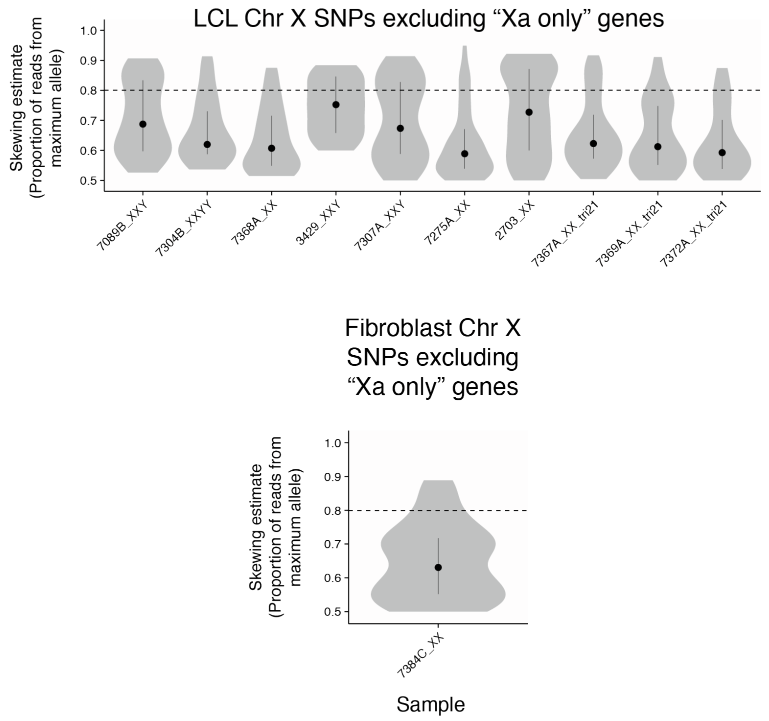

Figure S15. Presence of informative SNPs in Chr X genes not on the “Xa only” list distinguish “highly skewed” samples from those with identical copies of Chr X, related to Figure 4. Samples with ≤ 5 SNPs in “Xa only” genes have informative SNPs in other Chr X genes. This indicates that these X chromosomes are not identical, but that they are so highly skewed that informative SNPs are not identified in “Xa only” genes (due to the requirement for at least 3 reads mapping to the reference and alternative alleles).

Figure S16.

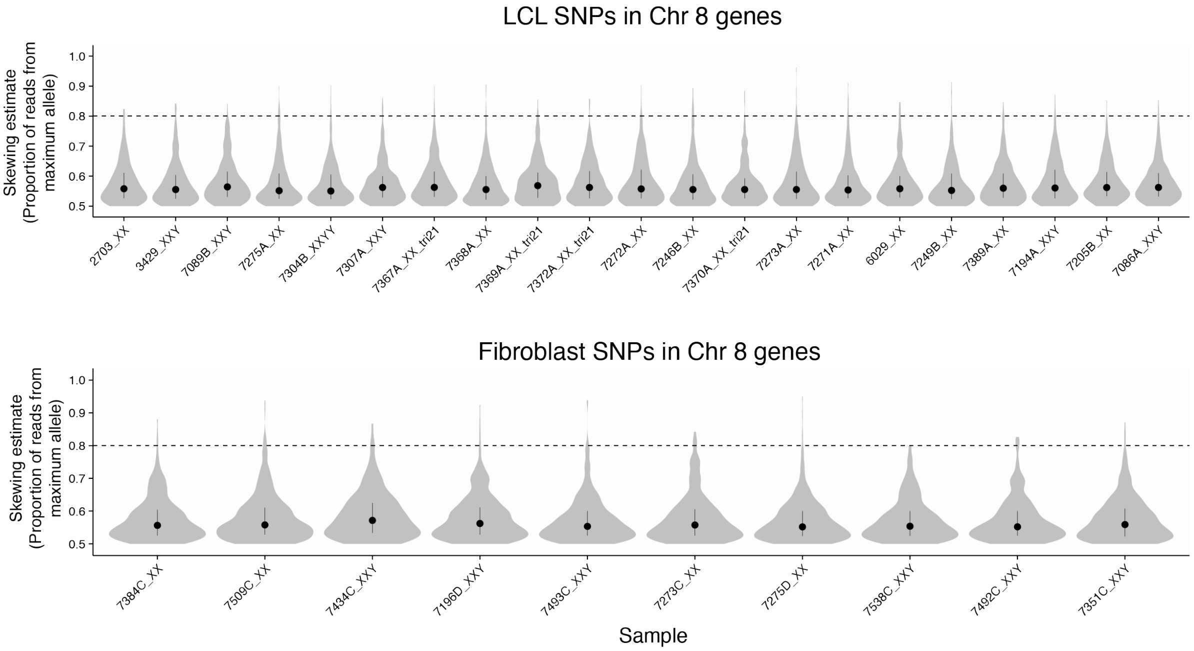

Figure S16. SNPs in Chr 8 genes do not show skewing, indicating that skewing is specific to Chr X and not an artifact observed genome-wide, related to Figure 4. Skewing analysis was performed using SNPs in expressed genes on Chr 8 on samples with skewed XCI. Note uniform skewing estimates of <0.6 across LCL and fibroblast samples, indicating relatively equal expression of each allele.

Figure S17.

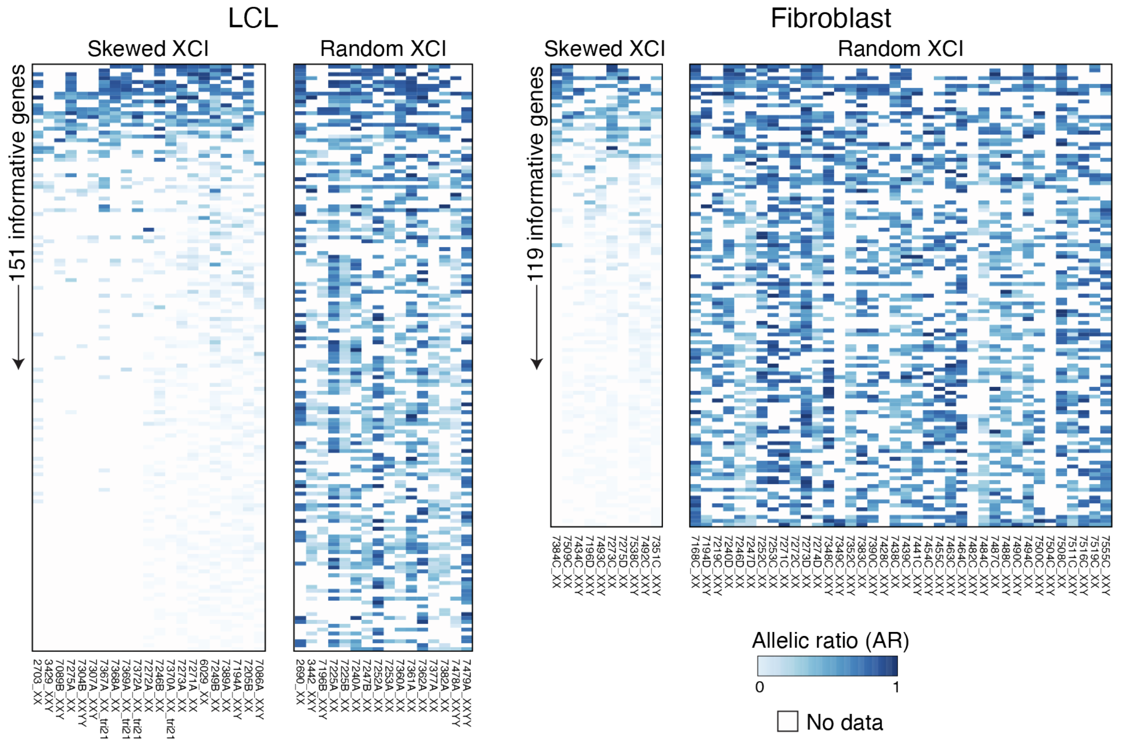

Figure S17. Genes with high and low allelic ratios can be distinguished in samples with skewed XCI, but not in samples with random XCI, related to Figure 4.

Heatmaps showing adjusted allelic ratio data for informative genes (those with data in at least 3 samples with skewed XCI) in LCLs or fibroblasts. Genes (rows) are ordered by the average adjusted AR across samples with skewed XCI. Raw AR values for samples with random XCI (those that did not reach the 0.8 median skewing threshold) are shown for comparison.

Figure S18.

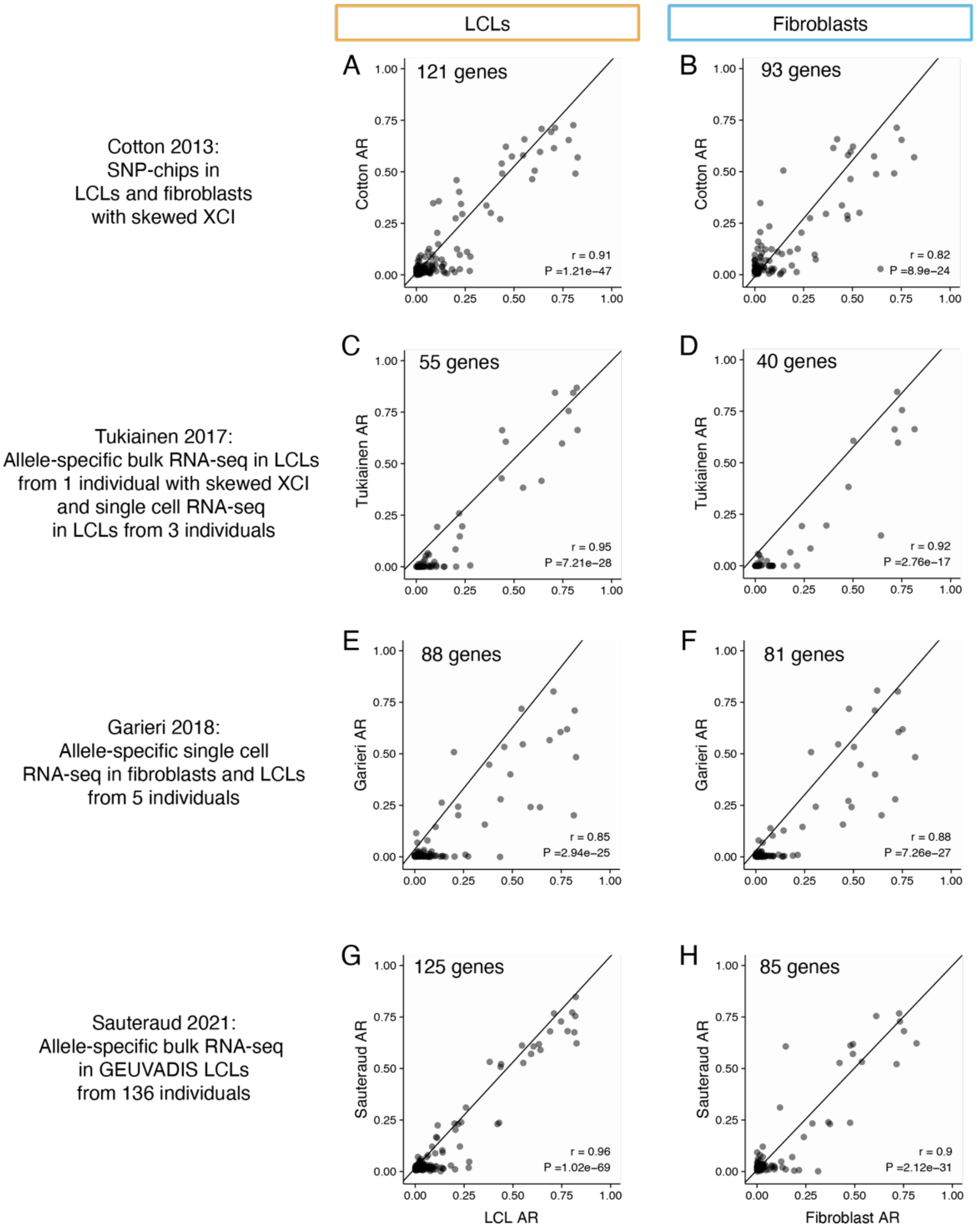

Figure S18. Allelic ratios calculated in this study are correlated with published allelic ratios, related to Figure 4. Scatterplots of the AR values calculated in this study versus AR values from published studies [S5-8]. Genes that were informative in both studies are included on the plots. Deming regression lines, Pearson correlation and P-values are indicated.

Figure S19.

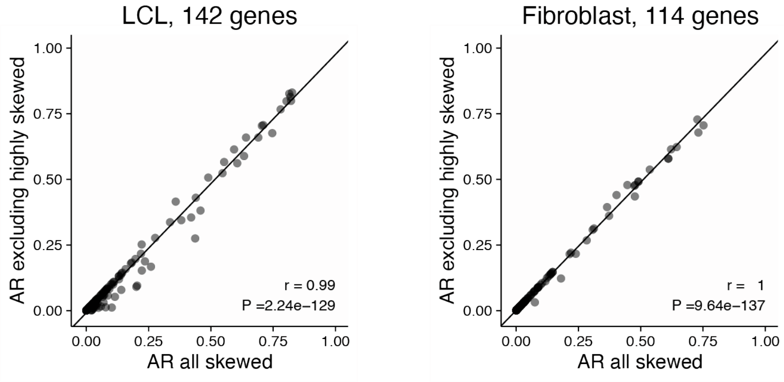

Figure S19. Including highly-skewed samples does not result in overestimation of allelic ratios, related to Figure 4. Scatterplots comparing AR calculated using all skewed samples, or without the "highly skewed" samples. Deming regression lines, Pearson correlation and P-values are indicated.

References

[S1] Goto, Y., and Kimura, H. (2009). Inactive X chromosome-specific histone H3 modifications and CpG hypomethylation flank a chromatin boundary between an X-inactivated and an escape gene. Nucleic Acids Res. *37*, 7416-7428. 10.1093/nar/gkp860.

[S2] Chen, C.Y., Shi, W., Balaton, B.P., Matthews, A.M., Li, Y., Arenillas, D.J., Mathelier, A., Itoh, M., Kawaji, H., Lassmann, T., et al. (2016). YY1 binding association with sex-biased transcription revealed through X-linked transcript levels and allelic binding analyses. Sci. Rep. *6*, 37324. 10.1038/srep37324.

[S3] Balaton, B.P., Fornes, O., Wasserman, W.W., and Brown, C.J. (2021). Cross-species examination of X-chromosome inactivation highlights domains of escape from silencing. Epigenetics Chromatin *14*, 12. 10.1186/s13072-021-00386-8.

[S4] Raznahan, A., Parikshak, N.N., Chandran, V., Blumenthal, J.D., Clasen, L.S., Alexander-Bloch, A.F., Zinn, A.R., Wangsa, D., Wise, J., Murphy, D.G.M., et al. (2018). Sex-chromosome dosage effects on gene expression in humans. Proc. Natl. Acad. Sci. USA *115*, 7398-7403. 10.1073/pnas.1802889115.

[S5] Cotton, A.M., Ge, B., Light, N., Adoue, V., Pastinen, T., and Brown, C.J. (2013). Analysis of expressed SNPs identifies variable extents of expression from the human inactive X chromosome. Genome Biol. *14*, R122. 10.1186/gb-2013-14-11-r122.

[S6] Tukiainen, T., Villani, A.C., Yen, A., Rivas, M.A., Marshall, J.L., Satija, R., Aguirre, M., Gauthier, L., Fleharty, M., Kirby, A., et al. (2017). Landscape of X chromosome inactivation across human tissues. Nature *550*, 244-248. 10.1038/nature24265.

[S7] Garieri, M., Stamoulis, G., Blanc, X., Falconnet, E., Ribaux, P., Borel, C., Santoni, F., and Antonarakis, S.E. (2018). Extensive cellular heterogeneity of X inactivation revealed by single-cell allele-specific expression in human fibroblasts. Proc. Natl. Acad. Sci. USA *115*, 13015-13020. 10.1073/pnas.1806811115.

[S8] Sauteraud, R., Stahl, J.M., James, J., Englebright, M., Chen, F., Zhan, X., Carrel, L., and Liu, D.J. (2021). Inferring genes that escape X-Chromosome inactivation reveals important contribution of variable escape genes to sex-biased diseases. Genome Res. *31*, 1629-1637. 10.1101/gr.275677.121.
