# Supplementary material for: The human inactive X chromosome modulates expression of the active X chromosome
Source: Cell Genom. 2023 Feb 8;3(2):100259. doi: 10.1016/j.xgen.2023.100259 (PMC9932992; doi:10.1016/j.xgen.2023.100259)
Supplement: Document S2. Article plus supplemental information [file mmc9.pdf]

Cell Genomics

Article

The human inactive X chromosome modulates expression of the active X chromosome

Graphical abstract

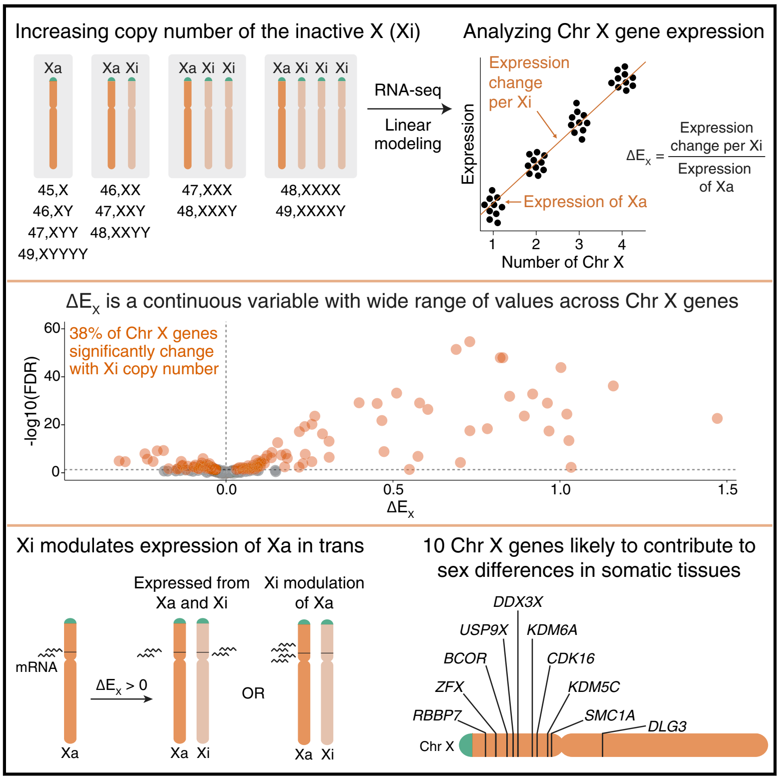

Highlights

- Analyzed gene expression in sex chromosome aneuploidy samples using linear models
- Xi and Xa transcriptomes are modular
- 38% of X chromosome genes are affected by Xi copy number—in *cis* and in *trans*
- 10 X chromosome genes likely contribute to male-female differences in somatic tissues

Authors

Adrianna K. San Roman, Alexander K. Godfrey, Helen Skaletsky, ..., Carole Samango-Sprouse, Maximilian Muenke, David C. Page

Correspondence

dcpage@wi.mit.edu

In brief

Through RNA sequencing of individuals with sex chromosome aneuploidy, San Roman et al. identify modular "active" (Xa) and "inactive" (Xi) X chromosome transcriptomes. Looking beyond classical X inactivation, which acts in *cis*, they find that Xi modulates Xa transcript levels in *trans*. They identify 10 X chromosome genes most likely to contribute to male-female differences in common disease.

San Roman et al., 2023, Cell Genomics *3*, 100259
February 8, 2023 © 2023 The Authors.
https://doi.org/10.1016/j.xgen.2023.100259

CellPress

Cell Genomics

CellPress
OPEN ACCESS

Article

The human inactive X chromosome modulates expression of the active X chromosome

Adrianna K. San Roman,[1] Alexander K. Godfrey,[1,2] Helen Skaletsky,[1,3] Daniel W. Bellott,[1] Abigail F. Groff,[1] Hannah L. Harris,[1,2] Laura V. Blanton,[1] Jennifer F. Hughes,[1] Laura Brown,[1,3] Sidaly Phou,[1,3] Ashley Buscetta,[4] Paul Kruszka,[4,12] Nicole Banks,[4,5] Amalia Dutra,[6] Evgenia Pak,[6] Patricia C. Lasutschinkow,[7] Colleen Keen,[7] Shanlee M. Davis,[8] Nicole R. Tartaglia,[8,9] Carole Samango-Sprouse,[7,10,11] Maximilian Muenke,[4,13] and David C. Page[1,2,3,14,*]

[1]Whitehead Institute, Cambridge, MA 02142, USA
[2]Department of Biology, Massachusetts Institute of Technology, Cambridge, MA 02139, USA
[3]Howard Hughes Medical Institute, Whitehead Institute, Cambridge, MA 02142, USA
[4]Medical Genetics Branch, National Human Genome Research Institute, National Institutes of Health, Bethesda, MD 20892, USA
[5]Eunice Kennedy Shriver National Institute of Child Health and Human Development, National Institutes of Health, Bethesda, MD 20892, USA
[6]Cytogenetics and Microscopy Core, National Human Genome Research Institute, National Institutes of Health, Bethesda, MD 20892, USA
[7]Focus Foundation, Davidsonville, MD 21035, USA
[8]Department of Pediatrics, University of Colorado School of Medicine, Aurora, CO 80045, USA
[9]Developmental Pediatrics, eXtraOrdinarY Kids Program, Children's Hospital Colorado, Aurora, CO 80011, USA
[10]Department of Pediatrics, George Washington University, Washington, DC 20052, USA
[11]Department of Human and Molecular Genetics, Florida International University, Miami, FL 33199, USA
[12]Present addresses: GeneDx, Gaithersburg, MD 20877, USA
[13]Present addresses: American College of Medical Genetics and Genomics, Bethesda, MD 20814, USA
[14]Lead contact
*Correspondence: dcpage@wi.mit.edu
https://doi.org/10.1016/j.xgen.2023.100259

SUMMARY

The "inactive" X chromosome (Xi) has been assumed to have little impact, in *trans*, on the "active" X (Xa). To test this, we quantified Xi and Xa gene expression in individuals with one Xa and zero to three Xis. Our linear modeling revealed modular Xi and Xa transcriptomes and significant Xi-driven expression changes for 38% (162/423) of expressed X chromosome genes. By integrating allele-specific analyses, we found that modulation of Xa transcript levels by Xi contributes to many of these Xi-driven changes (≥121 genes). By incorporating metrics of evolutionary constraint, we identified 10 X chromosome genes most likely to drive sex differences in common disease and sex chromosome aneuploidy syndromes. We conclude that human X chromosomes are regulated both in *cis*, through Xi-wide transcriptional attenuation, and in *trans*, through positive or negative modulation of individual Xa genes by Xi. The sum of these *cis* and *trans* effects differs widely among genes.

INTRODUCTION

The X chromosome of eutherian mammals exists in two distinct epigenetic states that are referred to as "active" (Xa) and "inactive" (Xi).[1–3] The "n−1" rule (where n is the number of X chromosomes per cell) states that all diploid human somatic cells possess one X chromosome in the active state (Xa), while all other (i.e., n−1) copies of chromosome (Chr) X[4] are transcriptionally repressed through a mechanism known as X chromosome inactivation (XCI). Despite the name, Xi is functionally active, making critical contributions to human fitness and viability. For example, 99% of fetuses with only one sex chromosome (45,X) abort spontaneously, suggesting that viability hinges on gene expression from a second sex chromosome—either Xi or Y.[5,6] The rare survivors likely have a mixture of 45,X cells and cells with a second sex chromosome, and they display a constellation of anatomic features known as Turner syndrome.[7,8]

Studies have revealed that as many as a quarter of X-linked genes are expressed from Xi in humans; such genes are said to "escape" X inactivation.[9] Early studies demonstrated the expression of certain Chr X genes on Xi ("escape") in human-rodent hybrid cell lines that had retained human Xi but had lost human Xa (for example, Mohandas et al., 1980; Brown et al., 1997; and Carrel et al., 1999).[10–12] Subsequent allele-specific methods distinguished transcripts from Xa and Xi in human cell lines that exhibited skewed XCI or in single cells.[13–18] While conceptually superior to hybrid cell lines, allele-specific methods yielded sparse data because they require the presence of heterozygous single-nucleotide polymorphisms (SNPs) to differentiate between alleles. Other studies approximated the contributions of Xi to X-linked gene expression by comparing samples with varying Xi copy numbers: in some cases, between 46,XY and 46,XX samples, and in others, between sex chromosome aneuploid and euploid samples.[15,19–26] These studies employed analytic

© 2023 The Authors.
This is an open access article under the CC BY license (http://creativecommons.org/licenses/by/4.0/).

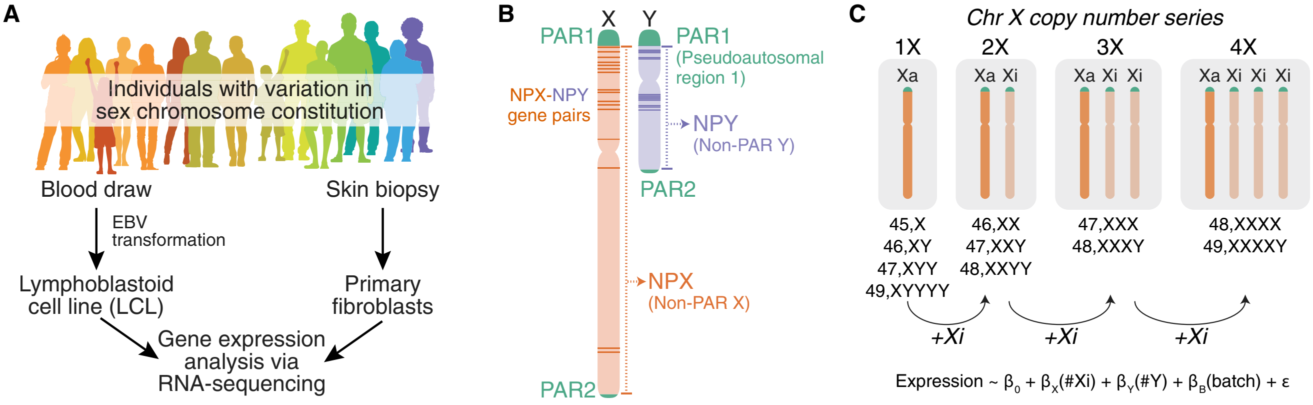

Figure 1. Gene expression analysis of cells from across the spectrum of sex chromosome constitution
(A) Collection and processing of samples from individuals with variation in sex chromosome constitution.
(B) Schematic of the sex chromosomes featuring the X-Y-shared pseudoautosomal regions, PAR1 and PAR2, and the diverged regions, NPX and NPY.
(C) Linear modeling strategy for analyzing RNA-seq data from individuals with one to four copies of Chr X (zero to three copies of Xi).
See also Table S1.

methods that made it difficult to separate the effect of Xi copy number from the potentially confounding effects of correlated factors such as Chr Y copy number, hormonal differences, or tissue composition. More importantly—as underscored by this study—previous work assumed, without directly testing, the independence and additivity of Xi and Xa expression. In particular, these studies assumed that any increase in expression observed with additional copies of Xi was due to expression from Xi, which may not always be the case. Given these limitations, we hypothesized that revisiting Xi gene expression with alternative experimental and analytic methods would reveal new insights.

Here, we used a series of quantitative approaches to investigate gene expression from Xi and Xa. Inspired by previous studies, we took advantage of the natural occurrence of diverse sex chromosome aneuploidies in the human population. We performed RNA sequencing (RNA-seq) on two cell types (lymphoblastoid cell lines and primary skin fibroblasts) from 176 individuals spanning 11 different sex chromosome constitutions—from 45,X (Turner syndrome) to 49,XXXXY. We analyzed the resulting data from these 176 individuals using linear regression models to identify significant changes in Chr X gene expression in identically cultured cells with zero, one, two, or three copies of Xi. 38% of Chr X genes displayed significant Xi-driven expression changes, which we quantified on a gene-by-gene basis using a novel metric that we developed called ΔE_X. By combining ΔE_X findings with allele-specific analyses performed in the same cell lines and comparing our results with published, independent annotations of genes subject to XCI, we found that Xi positively or negatively modulates steady-state levels of transcripts of at least 121 genes on Xa, in *trans*. Thus, Xi and Xa expression are highly interdependent. By combining ΔE_X with published gene-wise metrics of evolutionary constraint, we identified a set of 10 Chr X genes most likely to drive phenotypes that are associated with natural variation in Xi copy number. These 10 candidate "drivers" can now be prioritized in studies of sex differences in common disease and in explorations of sex chromosome aneuploidy syndromes.

RESULTS

Sampling gene expression across sex chromosome constitutions

To conduct a robust, quantitative analysis of Xi's impacts on X-linked gene expression, we recruited individuals with a wide range of sex chromosome constitutions to provide blood samples and/or skin biopsies (Figure 1A). We generated or received Epstein Barr virus-transformed B cell lines (lymphoblastoid cell lines [LCLs]) and/or primary dermal fibroblast cultures from 176 individuals with one to four X chromosomes and zero to four Y chromosomes. After culturing cells under identical conditions, we profiled gene expression by RNA-seq in LCLs from 106 individuals and fibroblast cultures from 99 individuals (some individuals contributed both blood and skin samples; Tables 1 and S1). To enable analysis at both the gene and transcript isoform levels, we generated 100-bp paired-end RNA-seq reads to a median depth of 74 million reads per sample. A resampling (bootstrapping) analysis of our dataset indicated that including more individuals with sex chromosome aneuploidy would only marginally increase the number of differentially expressed genes detected in our analyses (Figure S1; STAR Methods).

A metric for the impact of Xi on gene expression

To leverage the full power of our datasets, we compiled all RNA-seq data for each cell type into a single analysis. We included protein-coding and well-characterized long non-coding RNA (lncRNA) genes with a median expression in either 46,XX or 46,XY samples of at least 1 transcript per million (TPM). This resulted in 357 Chr X genes expressed in LCLs and 393 expressed in fibroblasts. Combining these two gene lists, we analyzed 423 Chr X genes in all. These genes reside within structurally and evolutionarily distinct regions (Figure 1B): two pseudoautosomal regions (PAR1 and PAR2), which are identical in sequence between Chr X and Y, and the non-pseudoautosomal region of the X (NPX), which has diverged in structure and gene content from the non-pseudoautosomal region of the Y (NPY).[27,28]

Table 1. Samples included in sex chromosome aneuploidy analysis

Karyotype	# LCLs	# Fibroblast cultures
45,X	17	23
46,XX	22	20
46,XY	17	14
47,XXX	7	4
47,XXY	11	30
47,XYY	10	5
48,XXXX	1	0
48,XXXY	4	1
48,XXYY	3	0
49,XXXXY	12	1
49,XYYYY	2	1
Total:	*106*	*99*

Despite this divergence, 17 homologous "NPX-NPY pair genes" with varying degrees of X-Y similarity in sequence and function remain.[27,29]

We hypothesized that each copy of Xi would incrementally increase expression of some Chr X genes, and therefore, for each gene, we modeled expression as a linear function of Xi copy number, controlling for Chr Y copy number and batch (Figure 1C; STAR Methods). To assess whether expression of each Chr X gene changed linearly per Xi, we fit non-linear least square regression models to the expression data using power functions (STAR Methods). Most NPX and PAR1 genes previously annotated as escaping XCI were best fit by linear models in which expression increases by a fixed amount per Xi, while most genes previously annotated as subject to XCI were best fit by models with no change in expression per Xi (Figure S2; see STAR Methods for the derivation of XCI status annotations from published studies). These results validate the "n−1" rule at the transcriptomic level, indicating that each cell has a single copy of Xa and n−1 copies of Xi. Moreover, linear modeling revealed that contributions by Xi to Chr X gene expression are strikingly modular, meaning that each Xi is more or less equivalent.

Linear models allowed us to identify genes whose expression changed significantly with additional copies of Xi and to quantify the absolute changes in expression (i.e., changes in read counts per Xi). To compare genes expressed at different levels, we also quantified the relative changes in expression per Xi. Specifically, we divided the change in expression per Xi (slope of regression, β_X) by the expression from the single Xa (average intercept across batches, β_0)—a metric we refer to as ΔE_X (Figure 2A). $\Delta E_X = 0$ indicates that adding one or more copies of Xi does not affect the level of expression (e.g., *PRPS2*; Figures 2B and S3); $\Delta E_X > 0$ indicates that expression increases under these circumstances (e.g., *KDM5C*; Figures 2C and S4), $\Delta E_X = 1$ indicates that Xa and Xi contribute equally; and $\Delta E_X < 0$ indicates that expression decreases (e.g., *F8*; Figures 2D and S5). *XIST,* the lncRNA that acts in *cis* to transcriptionally repress X chromosomes from which it is expressed,[30,31] was the only gene without detectable expression in cells with one copy of Chr X (Xa) that was expressed robustly in cells with one or more inactive copies (Xi). Considering samples with two or more X chromosomes, we found that *XIST* expression increased linearly with each additional copy of Xi (Figures 2E and S4).

ΔE_X values vary widely among Chr X genes but not between cell types

Analyzing ΔE_X values across Chr X genes revealed that Xi contributions to expression varied widely. Of 357 Chr X genes expressed in LCLs, 235 (66%) showed no significant change in expression level with additional copies of Xi ($\Delta E_X \approx 0$), and the same was true for 304 (77%) of 393 Chr X genes expressed in fibroblasts (Figures 2F and 2G; full results in Table S2). This is consistent with these genes being expressed—in the respective cell types—from a cell's first X chromosome (Xa) and silencing on all others (Xi). The remaining 122 (34%) Chr X genes expressed in LCLs and 89 (23%) Chr X genes expressed in fibroblasts had significantly negative or positive ΔE_X values. Combining the results in LCLs and fibroblasts, Xi copy number significantly impacts gene expression levels for 162 of 423 (38%) Chr X genes expressed in one or both cell types. NPX genes' ΔE_X values ranged from −0.39 to 1.2 (Figures 2F and 2G). PAR1 genes had ΔE_X values near one, while PAR2 genes had values near zero (Table S2). The stark difference between PAR1 and PAR2 likely reflects their evolutionary origins: PAR1 was preserved on Chr X and Y through sex chromosome evolution and retains autosome-like features, while PAR2 evolved later through a transposition from Chr X to Chr Y.[32] For nearly all Chr X genes, the change in expression per Xi falls short of that contributed by Xa (ΔE_X<1), similar to previous studies using allelic ratio analysis.[13,14] Only two NPX genes—*XIST* and *PUDP*—and three PAR1 genes—*DHSRX, PLCXD1,* and *PPP2R3B*—showed ΔE_X values approaching or exceeding one in both LCLs and fibroblasts.

We assessed whether these Chr X expression dynamics were influenced by factors apart from Xi count. We found few differences between cell types; genes expressed in both LCLs and fibroblasts displayed concordant ΔE_X values (Figure 2H). This is consistent with studies of differential expression between 46,XY and 46,XX tissues that found correlated expression changes for Chr X genes across diverse tissues.[15] To control for any effects of gonadal sex or Y chromosome copy number on our results, we reanalyzed the data from samples with zero (45,X; 46,XX; 47,XXX; 48,XXXX) or one copy of Chr Y (46,XY; 47,XXY; 48,XXXY; 49,XXXXY), modeling expression as a function of Xi copy number and batch. ΔE_X values were unaffected by the presence or absence of a Y chromosome (Figure S6). Because of its design, our study reveals that these consistent Chr X expression dynamics derive from direct, cell-autonomous contributions of Xi rather than systemic effects of hormones or environmental factors.

For genes with multiple transcript isoforms (alternative transcripts), we asked whether ΔE_X values were consistent between isoforms (STAR Methods). For most genes, transcript isoforms displayed concordant ΔE_X values. However, for genes with multiple transcript isoforms, 33 (19%) in LCLs and 25 (13%) in fibroblasts had discordant ΔE_X values: at least one isoform's ΔE_X differed significantly from zero (false discovery rate [FDR] < 0.05), while another isoform's ΔE_X did not (Figure S7; Table S3). The most striking case is that of *UBA1*, where

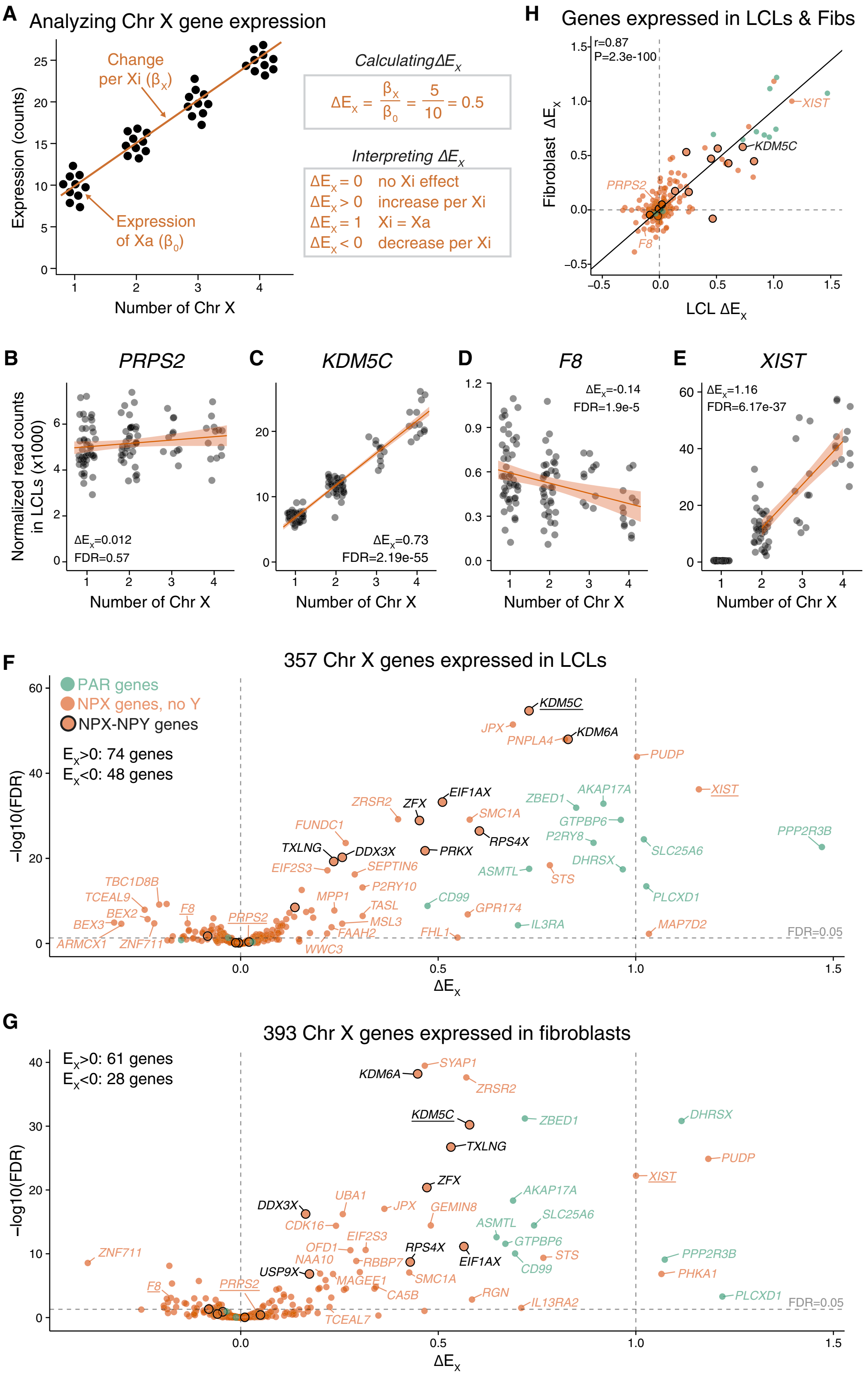

(legend on next page)

CellPress
OPEN ACCESS

alternative transcription start sites, separated by a CTCF binding site, display divergent behaviors (Figure S8).

To assess reproducibility, we compared our results with those from an independent dataset that used microarrays to assay gene expression in LCLs across diverse sex chromosome constitutions.[24] Reanalyzing this dataset using linear models, we found that the resulting microarray ΔE_X values correlated well with the ΔE_X values calculated from our RNA-seq data (Figure S9; STAR Methods).

Supernumerary copies of Chr Y and 21 show little attenuation of gene expression

To determine whether the attenuated expression observed with extra copies of Chr X also occurs with additional copies of other chromosomes, we analyzed cells from individuals with additional copies of Chr Y or with trisomy 21, a common autosomal aneuploidy and the cause of Down syndrome.[33]

For Chr Y, we used the same linear model as for Chr X: modeling expression as a function of Chr Y copy number, Xi copy number, and batch (Figure 3A; STAR Methods). We calculated ΔE_Y values separately for NPY and PAR genes because NPY genes are not expressed in samples with zero Y chromosomes, while PAR genes are expressed in all samples.

For NPY genes, we analyzed samples with one to four Y chromosomes to quantify expression differences, if any, between the first and additional Y chromosomes. Expression of all NPY genes increased significantly, with ΔE_Y values close to 1, consistent with near-equal expression from each copy of Chr Y (e.g., *KDM5D*; Figures 3B–3D and S10; full results in Table S4).

For PAR genes, we analyzed samples with zero to four Y chromosomes. As with Chr X, PAR1 gene expression increased with additional copies of Chr Y, yielding ΔE_Y values close to one, whereas PAR2 genes had ΔE_Y values near zero (Figures 3C and 3D). This implies, first, that PAR1 genes are expressed on each copy of Chr X or Y, while PAR2 genes are only expressed on the first copy of Chr X (Xa), and, second, that PAR1 gene expression from each additional Chr X or Y is roughly equal to expression from the first.

Finally, we examined Chr 21 gene expression as a function of Chr 21 copy number (Figures 3E and S11; STAR Methods). Nearly three-quarters of expressed Chr 21 genes significantly (FDR < 0.05) increased in expression with an additional copy of Chr 21 (e.g., *CCT8*; Figure 3F), and none significantly decreased (Figure 3G; Table S5). These results align well with independent studies of Chr 21 gene expression.[34]

In sum, unlike genes on Chr X, our analysis reveals that most genes on Chr Y and Chr 21 are expressed similarly on each copy of their respective chromosomes. The median ΔE values for Chr Y (including NPY and PAR1) and Chr 21 genes range from 0.74 to 1. By comparison, NPX genes without NPY homologs had median $\Delta E_X \approx 0$, while NPX genes with NPY homologs had modestly higher median ΔE_X values (LCLs: 0.26, fibroblasts: 0.17; Figure 3H). Even PAR1 genes, which as a group had the highest median ΔE_X values, were modestly attenuated on Xi compared with Chr Y, especially in LCLs (Figure 3H). This Y-vs.-X effect was most pronounced for *CD99*, located near the PAR1-NPX/Y boundary (Tables S2 and S4), consistent with suggestions that PAR1 gene expression on Xi is modestly attenuated by spreading of heterochromatin.[15] These differences highlight the absence of a chromosome-wide mechanism attenuating (or otherwise altering) gene expression on supernumerary copies of Chr Y and Chr 21, in contrast to Chr X.

Xi modulation of Xa transcript levels revealed by divergence of ΔE_X and allelic ratio

ΔE_X conveys the change in a gene's expression due to an additional Xi regardless of the mechanism(s) responsible for this change. We hypothesized that a Chr X gene's ΔE_X value could reflect the combined effects of two mechanisms: (1) transcription of Xi allele(s) and (2) modulation of the Xa allele by Xi in *trans*.

Seeking evidence of these mechanisms, we searched gene by gene for agreements and disagreements between our calculated ΔE_X values and published descriptions of the genes as "escaping" XCI (being expressed from Xi) or being subject to it (silenced on Xi). For this purpose, we curated annotations of each expressed gene's XCI status from studies of allele-specific expression[13–16,18] (STAR Methods; Table S6). Many genes with $\Delta E_X > 0$ were classified as expressed from Xi (50/74 in LCLs and 43/61 in fibroblasts), indicating that transcription from Xi alleles underlies their ΔE_X values (Figure 4A; Table S6). Genes with these characteristics overlapped significantly between fibroblasts and LCLs (Figure 4B).

For 102 (24%) of the 423 Chr X genes we evaluated in LCLs or fibroblasts, our calculated ΔE_X values were at odds with expectations arising from the published annotations of XCI status (Figures 4A and 4C; Table S6). For example, among genes with $\Delta E_X > 0$, 22 in LCLs and 14 in fibroblasts were described as silenced on Xi. Additionally, previous models offer no explanation for the 48 genes in LCLs and 28 genes in fibroblasts with $\Delta E_X < 0$, most of which were described as silenced on Xi (Figures 4A and 4C; Table S6). Genes with these characteristics did not overlap significantly between LCLs and fibroblasts, even though most are expressed in both cell types, indicating that this regulation is largely cell-type specific (Figure 4C). These

Figure 2. Quantitative assessment of Xi contributions to X chromosome gene expression

(A) Schematic scatterplot, linear regression line, and ΔE_X calculation for a hypothetical Chr X gene. Each point represents the expression level for an individual sample with the indicated number of copies of Chr X. The calculated coefficients from the linear model in Figure 1C are used to derive ΔE_X.

(B–E) Actual scatterplots and regression lines with confidence intervals for selected Chr X genes in LCLs, representing a range of ΔE_X values. Adjusted p values (FDR) < 0.05 indicate that ΔE_X values are significantly different from 0.

(F and G) Scatterplots of ΔE_X versus significance for all Chr X genes expressed in LCLs (F) and fibroblasts (G) illustrate variation in Xi contributions to Chr X gene expression; genes with FDR < 0.05 and $|\Delta E_X| \geq 0.2$ are labeled; genes depicted in (B)–(E) are underlined.

(H) Scatterplot comparing ΔE_X in LCLs and fibroblasts for 327 Chr X genes expressed in both cell types. Colors as in (F) and (G). Deming regression line and Pearson correlation are indicated.

See also Table S2.

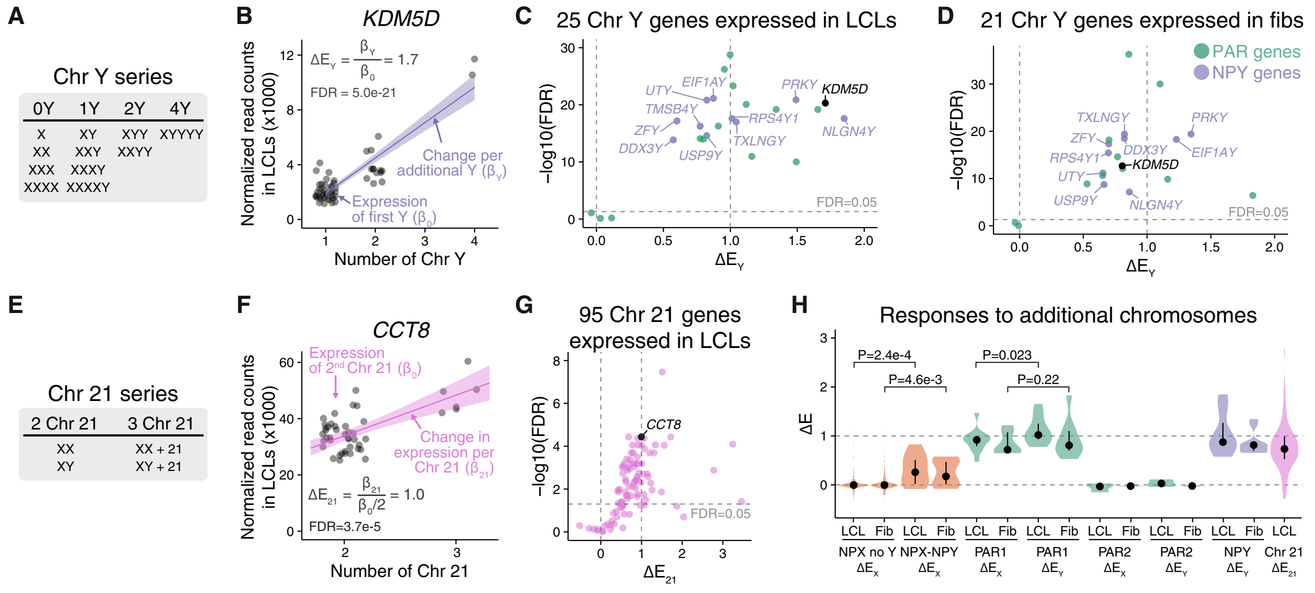

Figure 3. Contributions of Chr Y or 21 copy number to gene expression

(A) Chr Y copy number series with zero to four copies.

(B) Each point shows the expression of NPY gene *KDM5D* in one LCL sample across the Chr Y copy-number series, with the regression line and its confidence interval plotted. The formula for calculating ΔE_Y from the regression coefficients is indicated.

(C and D) Scatterplot of ΔE_Y versus significance for all Chr Y genes expressed in LCLs (C) or fibroblasts (D); all NPY genes are labeled; *KDM5D,* depicted in (B), is shown in black.

(E) Chr 21 copy-number series with two to three copies.

(F) Each point shows the expression of *CCT8* in one LCL sample across the Chr 21 copy-number series, with the regression line and its confidence interval plotted. The formula for calculating ΔE_{21} from the regression coefficients is indicated.

(G) Scatterplot of ΔE_{21} versus significance for all Chr 21 genes expressed in LCLs. *CCT8*, depicted in (F), is shown in black.

(H) Violin plots with median and interquartile range for ΔE values of NPX (without or with an NPY homolog), PAR, NPY, and Chr 21 genes. p values are listed for comparisons referenced in the text. ΔE_X values for NPX genes with and without a Y homolog were compared using Wilcoxon rank-sum test. ΔE_X and ΔE_Y values for PAR1 genes were compared using paired t test.

See also Tables S4 and S5.

unanticipated findings are unlikely to reflect experimental error in the previous or current studies. Instead, they suggest that, for many Chr X genes whose Xi allele(s) are silent, the Xa allele is nonetheless upregulated ($\Delta E_X > 0$) or downregulated ($\Delta E_X < 0$) by Xi.

To corroborate these findings in our own dataset, we performed an allele-specific analysis in our LCL and fibroblast samples with two X chromosomes. To distinguish between Chr X alleles, we identified heterozygous SNPs in expressed genes (STAR Methods). We then identified samples in our dataset with skewed XCI (21 LCL and 10 fibroblast samples; Figures S12–S16) and used these samples to compute the average ratio of Xi to Xa expression (the allelic ratio [AR]) for each gene. To calculate the AR, we required heterozygous SNPs in at least three samples, resulting in AR values for 151 genes in LCLs and 119 in fibroblasts (Table S6; Figure S17). In LCLs and fibroblasts, respectively, 38 (25%) and 22 (18%) of these genes had AR values significantly greater than zero, indicating that they are expressed from Xi (Figures 4D and 4E); these results agreed well with published AR values (Figure S18).

We next compared each gene's AR and ΔE_X values. If Xi and Xa expression are fully independent of each other, and therefore additive, we would expect the AR for a given gene to approximate its ΔE_X value. However, if Xi modulates the gene's Xa transcript levels in *trans*, then independence and additivity will not be observed, and instead the gene's ΔE_X and AR values will differ. Most X-linked genes, e.g., *EIF2S3*, had AR values that approximate their ΔE_X values, and AR and ΔE_X were highly correlated among many informative genes in both LCLs and fibroblasts (Figures 4F and 4G). For these genes, the ΔE_X value may directly reflect the level of transcription from Xi.

However, for 33 informative genes in LCLs or fibroblasts, AR and ΔE_X were significantly different, indicating that Xi modulated Xa transcript levels upward or downward in *trans* (Figures 4F–4H; STAR Methods). Some genes, like *MPP1,* were not expressed from Xi (AR ≈ 0) but nonetheless had ΔE_X values significantly different from zero: 0.24 in LCLs and 0.21 in fibroblasts, indicating that levels of Xa-derived transcripts are positively regulated by Xi. Other genes, like *DDX3X* and *PUDP*, had significant expression from Xi (AR > 0, FDR < 0.05) and evidence of Xi regulation of steady-state expression levels. *DDX3X* had an AR (LCLs: 0.55, fibroblasts: 0.42) that is significantly higher than its ΔE_X value (LCLs: 0.26, fibroblasts: 0.16) in both LCLs and fibroblasts, indicating both that *DDX3X* is expressed on Xi and that its steady-state transcript levels are negatively regulated by Xi. Conversely, *PUDP* had an AR (LCLs: 0.71, fibroblasts: 0.73)

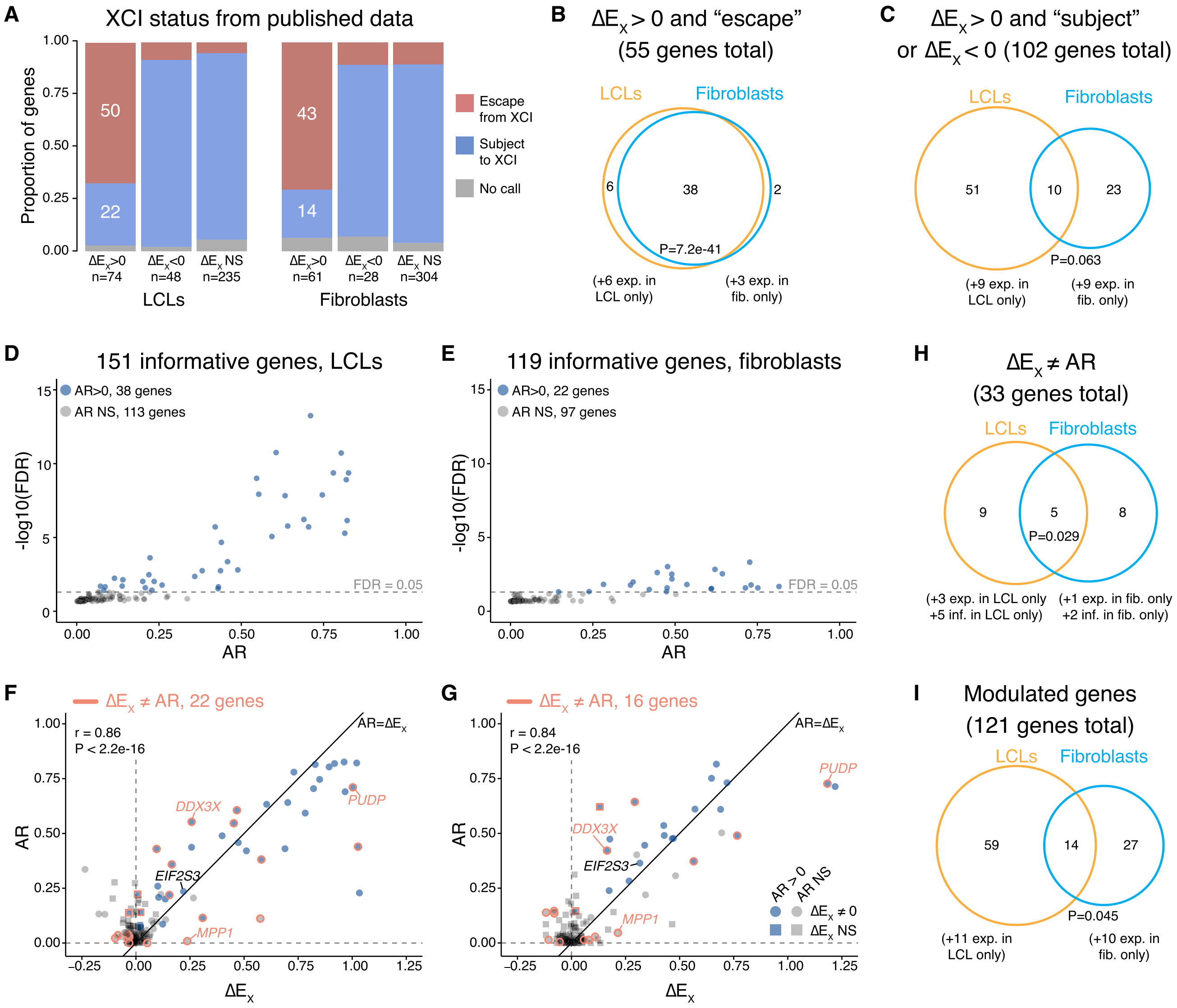

Figure 4. Comparison of ΔE_X values with allelic ratios (ARs) reveals that Xi modulates Xa expression

(A) Stacked barplots for genes with ΔE_X values greater than, less than, or approximately equal to zero, apportioned by their annotated XCI status from published studies (see STAR Methods and Table S6 for newly compiled XCI status calls).

(B and C) Venn diagrams comparing LCLs and fibroblasts for genes with ΔE_X values that are either (B) explained or (C) not explained by published XCI status. Genes expressed in both cell types were included in the Venn diagrams, and genes with cell-type-specific expression are noted below.

(D and E) Each point shows the mean adjusted AR for an informative gene (with heterozygous SNPs in at least 3 samples with skewed XCI) and whether AR is significantly greater than zero in (D) LCLs or (E) fibroblasts.

(F and G) Each point denotes AR and ΔE_X values for an AR-informative gene in (F) LCLs or (G) fibroblasts. The color of the point indicates whether the gene's AR value is significantly greater than zero (blue) or not (gray); the shape indicates whether the gene's ΔE_X value is significantly different from zero (circles) or not (squares); and an orange outline indicates that ΔE_X differs significantly from AR. Black diagonal line, AR = ΔE_X. Pearson correlation coefficients (r) and p values are indicated.

(H) Venn diagram comparing LCLs and fibroblasts for genes with ΔE_X values not equal to their AR values. Genes expressed and informative in both cell types are depicted in the Venn diagram, with genes that are cell-type specific or informative in only one cell type indicated below.

(I) Venn diagram comparing all modulated genes in LCLs and fibroblasts (the union of figures, C and H). All Venn diagram p values, hypergeometric test.

See also Table S6.

that is significantly lower than its ΔE_X value (LCLs: 1, fibroblasts: 1.2), indicating both that *PUDP* is expressed on Xi and that the gene's steady-state transcript levels are positively regulated by Xi.

These analyses, combining ΔE_X with published or newly derived AR data, provide a rich portrait of X-linked gene regulation. They show that Xi can impact expression levels of an X-linked gene through two mechanisms: transcription of the Xi

allele and modulation of steady-state transcript levels by Xi in *trans*. These mechanisms can operate independently of each other, or together, on a gene-by-gene basis, and each of the two mechanisms affects a sizable fraction of all X chromosome genes. Of 423 X chromosome genes expressed in LCLs and/or fibroblasts, at least 121 genes (29%) are modulated on Xa by Xi in one or both cell types (Figure 4I). This represents the union of the 102 genes for which the public AR data cannot explain the ΔE_X values (Figure 4C) and the 33 genes with AR values significantly different from ΔE_X (Figure 4H). The observed modulation of steady-state transcript levels suggests that Xi regulates the expression of genes on Xa in *trans*.

Combining ΔE_X and expression constraint metrics identifies likely drivers of Xi-associated phenotypes

While the somatic cells of all diploid individuals have one Xa, the number of Xis varies in the human population from zero to four. This variation is associated with many important differences in phenotypes and disease predispositions, for example those observed between 45,X (Turner syndrome) and 46,XX individuals, between 47,XXY (Klinefelter syndrome) and 46,XY individuals, or even between 46,XY males and 46,XX females. We hypothesized that phenotypes and predispositions associated with Xi copy number are due to changes in the copy numbers of some of the Chr X genes where we found positive or negative ΔE_X values. We reasoned that phenotypically critical genes would be "dosage sensitive", i.e., their expression levels would be tightly constrained by natural selection, while the expression levels of genes whose dosage is not phenotypically critical could vary with little consequence.

To gauge the constraints that selection has imposed on each gene's expression level, we turned to metrics derived from population and evolutionary genetic studies. We assessed tolerance of under-expression using (1) loss of function observed/expected upper fraction (LOEUF), the ratio of observed to expected loss-of-function (LoF) variants in human populations,[35] (2) RVIS, the residual variation intolerance score,[36] and (3) pHI, the probability of haploinsufficiency.[37] Both LOEUF and RVIS use large-scale human genomic sequencing data to evaluate selection against LoF variants, while pHI is based on evolutionary and functional metrics. LoF variants should be culled from the population in genes whose under-expression is deleterious, while they may accumulate in genes whose under-expression has little effect on fitness.

To assess tolerance of over-expression, we examined conservation of targeting by microRNAs (miRNAs; P_{CT} score[38]), which repress expression by binding to a gene's 3′ untranslated region.[39] Genes sensitive to over-expression have maintained their miRNA binding sites across vertebrate evolution, while genes whose over-expression has little or no effect on fitness show less conservation of these sites.[40]

To weigh these four metrics simultaneously, we calculated each gene's percentile rank for each metric, from most constrained (high percentile) to least constrained (low percentile). We calculated percentiles separately for autosomal (including PAR1) and NPX genes and then, for each gene, averaged percentile rankings across the four metrics.

We first examined expression constraints for PAR1 genes whose high ΔE_X values suggested that they may drive phenotypes associated with Xi copy number. Compared with autosomal genes, PAR1 genes are less constrained on average ($p = 5.5e{-}5$, Wilcoxon rank-sum test), with most ranking in the least-constrained quartile (Figures 5A and 5B; Table S7). This indicates that altering their expression levels has little impact on human fitness. Indeed, homozygous LoF mutations have been reported for 3 of 15 PAR1 genes, demonstrating dispensability.[35] Only two PAR1 genes, *SHOX* and *SLC25A6*, rank in the more constrained half of the comparison group (Table 2). *SHOX* copy number contributes to variation in height in individuals with sex chromosome anomalies,[41–45] while *SLC25A6* has not yet been linked to any phenotype. Apart from these two genes, the high tolerance of under- and over-expression for most PAR1 genes argues against prominent roles in phenotypes associated with Chr X (or X + Y) copy number.

Turning to the much larger set of NPX genes, we found that their widely ranging constraint metrics correlated poorly with their ΔE_X values (Figures 5C and 5D; Table S7). Thus, ΔE_X alone does not predict dosage sensitivity among NPX genes. To identify the NPX genes most likely to drive Xi-copy-number-dependent phenotypes, we selected those with $|\Delta E_X| \geq 0.1$ (FDR < 0.05) in LCLs or fibroblasts and ranked these by their average constraint metrics. Five of the top 10 genes by these criteria (Table 2) had NPY homologs, a significant enrichment ($p = 7.0e{-}4$, hypergeometric test), and all five had ΔE_X values > 0.1 in both cell types. Of the five genes without NPY homologs, only two had ΔE_X values significantly greater than zero in both cell types: *SMC1A* and *CDK16.* The remaining three genes had ΔE_X values significantly different from zero in only one of the two cell types analyzed, and they tended to have lower absolute ΔE_X values.

If these 10 genes are dosage-sensitive drivers of Xi-dependent phenotypes—even when harboring no mutations—then one might expect mutant phenotypes to be pronounced and to display distinctive modes of inheritance. Accordingly, we searched OMIM for disease annotations. Germline mutations in seven of the 10 genes are reported to cause severe developmental disorders, including well-characterized childhood syndromes for five of the genes (Tables S2 Table S7). Indeed, five of the seven mutation-bearing genes are reported to display dominant inheritance (affected heterozygous females)—a significant enrichment among X-linked genes ($p = 0.0037$, hypergeometric test) and consistent with extraordinary dosage sensitivity. Extrapolating from these findings, we speculate that some of the genes without annotated OMIM phenotypes may have important roles in disease; in the case of *ZFX*, no LoF mutations are reported in gnomAD even though the gene's roles in regulating stem cell self-renewal and cancer cell proliferation are well documented.[46–48] Taken together, these 10 genes represent good candidates for driving Xi-dependent phenotypes characteristic of individuals with sex chromosome aneuploidies—as well as differences in disease risks between ordinary (euploid) females and males.

DISCUSSION

We analyzed Chr X gene expression quantitatively in two types of cells cultured from individuals with one to four X chromosomes, e.g., 45,X to 49,XXXXY (Figure 1). Folding this diversity of sex

CellPress
OPEN ACCESS

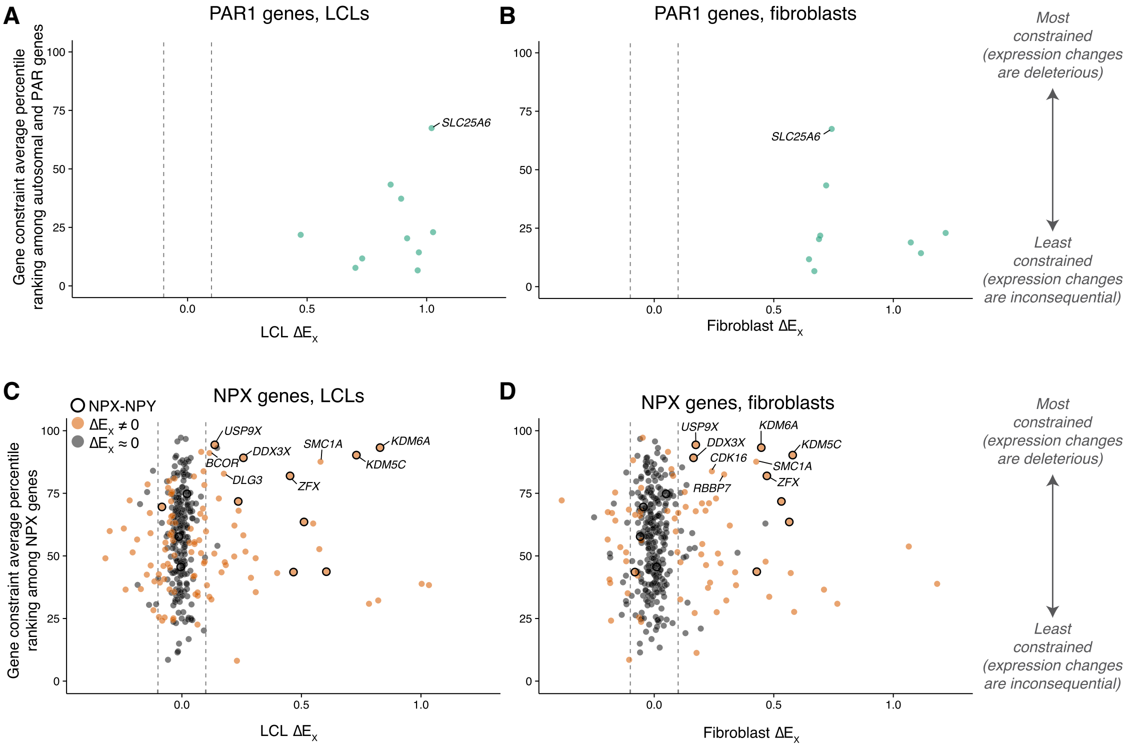

Figure 5. Combining ΔE_X with metrics of constraint on expression levels identifies genes likely to contribute to phenotypes associated with Xi copy number

Scatterplots of ΔE_X versus gene constraint percentile ranking for PAR1 (A and B) or NPX (C and D) genes. Each point represents an expressed gene with scores for at least two of the four expression constraint metrics evaluated, excluding ampliconic genes. Dashed lines indicate $|\Delta E_X|$ thresholds of 0.1 for genes to be considered likely contributors to phenotypes driven by Xi copy number; labeled genes include (A and B) *SLC25A6*, the only PAR1 gene to score above the 50th percentile for autosomal and PAR genes, and (C and D) among NPX genes with $|\Delta E_X| > 0.1$, the 10 genes with the highest constraint percentile rankings in LCLs or fibroblasts.

See also Table S7.

chromosome constitutions into a single linear model (Figure 2) yielded advantages over previous studies, which compared sex chromosome constitutions in pairwise fashion, most frequently 45,X vs. 46,XX; 46,XY vs. 47,XXY; or 46,XX vs. 46,XY. First, our linear model embodied, tested, and confirmed—at the level of the X transcriptome—the "n−1" rule,[4] whereby diploid somatic cells with a given number (n = 1, 2, 3, 4) of X chromosomes have a single Xa and n−1 Xi's. Second, linear modeling provided the power needed to detect and precisely quantify increases or decreases in expression of individual Chr X genes as a function of Chr X copy number. Third, linear modeling revealed that the expression contributions made by each copy of Xi are modular, indicating that each copy of Xi is equivalent, or nearly so, even among unrelated individuals. Fourth, by comparing samples that vary in Xi copy number with and without a Y chromosome, we found that expression from Xa is quantitatively indistinguishable in phenotypic males and females—as is expression from Xi (Figure S6). Thus, both Xi and Xa make modular contributions to Chr X gene expression—contributions independent of and unaffected by the presence of the NPY or the gonadal sex of the individual.

Finally, linear modeling of gene expression as a function of Chr X copy number yielded the metric ΔE_X, which captures the positive or negative impact of Xi(s) on steady-state transcript levels for each gene, normalized to account for gene-to-gene variation in expression level (Figure 2A). Fully 38% (162/423) of expressed Chr X genes in LCLs or fibroblasts displayed a statistically significant positive or negative ΔE_X value, indicating that their expression is impacted by the presence of one or more copies of Xi. This is nearly double what would be expected based on the prior literature's estimates of escape, which—based upon our reanalysis using the broadest definition of escape—includes only 20% (86/423) of expressed Chr X genes in these cell types (Table S6). ΔE_X values varied widely among Chr X genes, from −0.39 to 1.2 (Figures 2F and 2G), but showed much less variation between the two cell types studied (Figure 2H), suggesting the possibility that the ΔE_X "settings" for each gene were established prior to the embryonic divergence of the hematopoietic and skin fibroblast lineages and subsequently maintained through development.

We extended the utility of the ΔE_X metric by cross-referencing and comparing it, one gene at a time, with an orthogonal metric: the AR of Xi and Xa transcripts in cells with skewed XCI and SNP heterozygosity. AR values significantly greater than zero unambiguously identify Chr X genes that are expressed from both Xi and Xa (and therefore "escape" XCI).[49] By comparing ΔE_X and AR values, we discovered that Xi up- or downmodulates Xa

Table 2. X chromosome genes that may drive the phenotypic impacts of variation in Xi copy number

Region	Gene symbol	Gene name	NPY gene symbol	ΔE_X		Gene constraint (average % ranking)[a]	Disease associations		
				LCL	Fib.		Phenotype	Inheritance[b]	MIM #
NPX	*KDM6A*	lysine demethylase 6A	*UTY*	0.83	0.45	93.3	Kabuki syndrome	XLD	300867
	KDM5C	lysine demethylase 5C	*KDM5D*	0.73	0.58	90.3	Claes-Jensen syndrome	XLR	300534
	SMC1A	structural maintenance of chromosomes 1A	–	0.58	0.43	87.6	Cornelia de Lange syndrome; developmental and epileptic encephalopathy	XLD	300590, 301044
	ZFX	zinc finger protein X-linked	*ZFY*	0.45	0.47	83.0	–	–	–
	RBBP7	RB-binding protein 7, chromatin remodeling factor	–	0.01	0.29	82.5	–	–	–
	DDX3X	DEAD-box helicase 3 X-linked	*DDX3Y*	0.26	0.16	89.2	syndromic IDD,[c] Snijders Blok type	XLD, XLR	300958
	CDK16	Cyclin dependent kinase 16	–	0.09	0.24	83.8	–	–	–
	DLG3	discs large MAGUK scaffold protein 3	–	0.18	0.07	82.8	IDD	XLR	300580
	USP9X	ubiquitin-specific protease 9 X-linked	*USP9Y*	0.14	0.17	94.4	IDD	XLR, XLD	300919, 300968
	BCOR	BCL6 corepressor	–	0.12	0.01	91.1	oculofaciocardiodental syndrome	XLD	300166
PAR1	*SLC25A6*	solute carrier family 25 member 6	N/A	1.0	0.74	67.4	–	–	–
	SHOX	short stature homeobox	N/A	N/A[d]	N/A	58.4	Leri-Weill dyschondrosteosis; Langer mesomelic dysplasia; short stature idiopathic familial	PD, PR	127300, 249700, 300582

[a]Gene constraint percentile ranking is calculated for NPX genes relative to all annotated NPX genes and for PAR1 genes relative to all PAR and autosomal genes.
[b]XLD, X-linked dominant; XLR, X-linked recessive; PD, pseudoautosomal dominant; PR, pseudoautosomal recessive.
[c]IDD, intellectual developmental disorder.
[d]*SHOX* is not expressed in fibroblasts or LCLs but is included because its dosage has been conclusively linked to height in individuals with sex chromosome aneuploidy.

CellPress
OPEN ACCESS

expression of at least 121 genes, or nearly 29% of the 423 Chr X genes that are demonstrably expressed in either LCLs or fibroblasts. This modulation is manifest whenever a gene's AR and ΔE_X values differ significantly, and it is most starkly apparent when the gene is not expressed from Xi (i.e., when AR approximates zero) but nonetheless displays a significantly positive or negative ΔE_X value. While "escape" from XCI has been well documented over the past four decades,[12–15] the novel combination of the AR and ΔE_X metrics reported here was required to observe modulation, explaining why it has previously been unappreciated.

Thus, combined analysis of ΔE_X and AR reveals a nuanced, gene-by-gene tapestry of Xi-driven changes in expression of Chr X genes. For some genes, ΔE_X was explained entirely by expression from the Xi allele, while for others, ΔE_X was explained entirely by modulation—positive or negative—of steady-state RNA levels derived from the Xa allele. For a third set of genes, ΔE_X was explained by the combined effects of expression from Xi and modulation of steady-state RNA levels. Proposals of uniform, chromosome-wide "X chromosome upregulation" (XCU) during mammalian development or evolution[50,51] will need to be revisited in light of this unforeseen diversity of gene-by-gene responses to variation in Chr X copy number.

Finally, we paired the ΔE_X metric with population and evolutionary measures of constraint on expression levels to identify 10 NPX genes that are most likely (among the 423 Chr X genes expressed in LCLs and/or fibroblasts) to drive Xi-associated phenotypes (Figures 5C and 5D; Table 2). Despite their high ΔE_X values, most PAR genes did not exhibit the constraints on expression levels that we required for inclusion in this select group of candidate drivers (Figures 5A and 5B). We propose the 10 NPX genes—five of which have divergent NPY homologs—as potential drivers of (1) differences in health and disease between 46,XY and 46,XX cohorts and (2) the distinctive phenotypes associated with sex chromosome aneuploidies, including Turner syndrome (45,X) and Klinefelter syndrome (47,XXY). We speculate that one or more of these 10 NPX genes, which include transcriptional and epigenetic regulators, may also drive the modulation of Xa genes by Xi.

Limitations of the study

The human individuals sampled here are mostly of European ancestry; it will be important to validate these findings in a more ancestrally diverse set of individuals. Our findings in LCLs and fibroblasts were largely concordant, but they may not generalize to all somatic tissues and cell types. Our study focused on 423 Chr X genes that are expressed in LCLs and/or fibroblasts; our conclusions may not generalize to Chr X genes that are not expressed in these cell types. Our list of Chr X genes likely to drive Xi-dependent phenotypes is incomplete, as it is biased toward genes expressed in LCLs and fibroblasts and toward genes with long open reading frames well suited to expression constraint analysis; future studies will add to this list. In addition to these caveats regarding our current findings, several topics remain unexplored in this article and should be addressed in future studies; these include the molecular mechanisms by which Xi modulates gene expression on Xa, whether these mechanisms are direct or indirect, and whether these mechanisms also affect gene expression on autosomes.

STAR★METHODS

Detailed methods are provided in the online version of this paper and include the following:

- KEY RESOURCES TABLE
- RESOURCE AVAILABILITY
  - Lead contact
  - Materials availability
  - Data and code availability
- EXPERIMENTAL MODEL AND SUBJECT DETAILS
  - Human subjects
- METHOD DETAILS
  - Cell culture
  - Primary fibroblast cultures
  - Cell collection for subsequent analysis
  - RNA extraction, library preparation, and sequencing
  - RNA-seq data processing and analysis
  - Identifying genes affected by changes in chr X, Y, or 21 copy number
  - Saturation analysis for sex chromosome-encoded genes
  - Assessing linearity of sex-chromosome gene expression changes
  - ΔE_X calculations in samples with 0 Y chromosomes (females) and 1 Y chromosome (males)
  - Reanalysis of array data and comparison to RNA-seq data
  - Isoform-specific analysis of RNA-seq data
  - Gene constraint analysis
  - Comparisons to published annotations of X-inactivation status
  - Allele-specific expression analysis
- QUANTIFICATION AND STATISTICAL ANALYSES

SUPPLEMENTAL INFORMATION

Supplemental information can be found online at https://doi.org/10.1016/j.xgen.2023.100259.

ACKNOWLEDGMENTS

We thank members of the Page laboratory, especially Lukas Chmatal, for helpful comments on the manuscript, Sahin Naqvi for advice on RNA-seq and analysis, and Jorge Adarme and Susan Tocio for laboratory support. We thank the Whitehead Institute Genome Technology Core facility for library preparation and sequencing. This work was funded by the following: National Institutes of Health grants F32HD091966 (A.K.S.R.), U01HG0007587 (D.C.P. and M.M.), and K23HD092588 (S.M.D.); Schmidt Science Fellows (A.F.G.); Lallage Feazel Wall Damon Runyon Cancer Research Foundation Fellowship (L.V.B.); Howard Hughes Medical Institute (D.C.P.); National Human Genome Research Institute Intramural Research Program (M.M.); and NIH/NCATS Colorado CTSA grant UL1 TR002535 (N.R.T.). The contents are the authors' sole responsibility and do not necessarily represent official NIH views. We are also thankful for philanthropic gifts from Brit and Alexander d'Arbeloff, Arthur W. and Carol Tobin Brill, Matthew Brill, and Charles Ellis.

AUTHOR CONTRIBUTIONS

Conceptualization, A.K.S.R. and D.C.P.; data curation, A.K.S.R. and H.S.; formal analysis, A.K.S.R., A.K.G., H.S., D.W.B., A.F.G., H.L.H., L.V.B., and

J.F.H.; funding acquisition, A.K.S.R., A.F.G., L.V.B., S.M.D., N.R.T., M.M., and D.C.P.; investigation, A.K.S.R., S.P., L.B., A.D., and E.P.; methodology, A.K.S.R., A.K.G., H.S., A.F.G., and H.L.H.; project administration, A.K.S.R., J.F.H., L.B., A.B., P.K., N.B., P.C.L., C.K., and S.M.D.; resources, A.B., P.K., N.B., P.C.L., C.K., S.M.D., N.R.T., C.S.-S., and M.M.; software, A.K.S.R., A.K.G., H.S., A.F.G., H.L.H., and L.V.B.; supervision, A.K.S.R., J.F.H., N.R.T., C.S.-S., M.M., and D.C.P.; validation, A.K.G., H.S., D.W.B., and L.V.B.; visualization, A.K.S.R., A.K.G., and H.L.H.; writing – original draft preparation, A.K.S.R. and D.C.P.; writing – review and editing, A.K.S.R., A.K.G., D.W.B., A.F.G., H.L.H., L.V.B., J.F.H., S.M.D., and D.C.P.

DECLARATION OF INTERESTS

The authors declare no competing interests.

INCLUSION AND DIVERSITY

We support inclusive, diverse, and equitable conduct of research.

Received: March 2, 2022
Revised: August 12, 2022
Accepted: January 6, 2023
Published: February 8, 2023

REFERENCES

1. Ohno, S., and Hauschka, T.S. (1960). Allocycly of the X-chromosome in tumors and normal tissues. Cancer Res. *20*, 541–545.
2. Lyon, M.F. (1961). Gene action in the X-chromosome of the mouse (Mus musculus L.). Nature *190*, 372–373. https://doi.org/10.1038/190372a0.
3. Lyon, M.F. (1962). Sex chromatin and gene action in the mammalian X-chromosome. Am. J. Hum. Genet. *14*, 135–148.
4. Harnden, D.G. (1961). Nuclear sex in triploid XXY human cells. Lancet *2*, 488. https://doi.org/10.1016/s0140-6736(61)92457-6.
5. Hook, E.B., and Warburton, D. (1983). The distribution of chromosomal genotypes associated with Turner's syndrome: livebirth prevalence rates and evidence for diminished fetal mortality and severity in genotypes associated with structural X abnormalities or mosaicism. Hum. Genet. *64*, 24–27. https://doi.org/10.1007/BF00289473.
6. Hook, E.B., and Warburton, D. (2014). Turner syndrome revisited: review of new data supports the hypothesis that all viable 45,X cases are cryptic mosaics with a rescue cell line, implying an origin by mitotic loss. Hum. Genet. *133*, 417–424. https://doi.org/10.1007/s00439-014-1420-x.
7. Turner, H.H. (1938). A syndrome of infantilism, congenital webbed neck, and cubitus valgus. Endocrinology *23*, 566–574. https://doi.org/10.1210/endo-23-5-566.
8. Ford, C.E., Jones, K.W., Polani, P.E., Dealmeida, J.C., and Briggs, J.H. (1959). A sex-chromosome anomaly in a case of gonadal dysgenesis (turners syndrome). Lancet *1*, 711–713. https://doi.org/10.1016/s0140-6736(59)91893-8.
9. Balaton, B.P., Cotton, A.M., and Brown, C.J. (2015). Derivation of consensus inactivation status for X-linked genes from genome-wide studies. Biol. Sex Differ. *6*, 35. https://doi.org/10.1186/s13293-015-0053-7.
10. Mohandas, T., Sparkes, R.S., Hellkuhl, B., Grzeschik, K.H., and Shapiro, L.J. (1980). Expression of an X-linked gene from an inactive human X chromosome in mouse-human hybrid cells: further evidence for the noninactivation of the steroid sulfatase locus in man. Proc. Natl. Acad. Sci. USA *77*, 6759–6763. https://doi.org/10.1073/pnas.77.11.6759.
11. Brown, C.J., Carrel, L., and Willard, H.F. (1997). Expression of genes from the human active and inactive X chromosomes. Am. J. Hum. Genet. *60*, 1333–1343. https://doi.org/10.1086/515488.
12. Carrel, L., Cottle, A.A., Goglin, K.C., and Willard, H.F. (1999). A first-generation X-inactivation profile of the human X chromosome. Proc. Natl. Acad. Sci. USA *96*, 14440–14444. https://doi.org/10.1073/pnas.96.25.14440.
13. Carrel, L., and Willard, H.F. (2005). X-inactivation profile reveals extensive variability in X-linked gene expression in females. Nature *434*, 400–404. https://doi.org/10.1038/nature03479.
14. Cotton, A.M., Ge, B., Light, N., Adoue, V., Pastinen, T., and Brown, C.J. (2013). Analysis of expressed SNPs identifies variable extents of expression from the human inactive X chromosome. Genome Biol. *14*, R122. https://doi.org/10.1186/gb-2013-14-11-r122.
15. Tukiainen, T., Villani, A.C., Yen, A., Rivas, M.A., Marshall, J.L., Satija, R., Aguirre, M., Gauthier, L., Fleharty, M., Kirby, A., et al. (2017). Landscape of X chromosome inactivation across human tissues. Nature *550*, 244–248. https://doi.org/10.1038/nature24265.
16. Garieri, M., Stamoulis, G., Blanc, X., Falconnet, E., Ribaux, P., Borel, C., Santoni, F., and Antonarakis, S.E. (2018). Extensive cellular heterogeneity of X inactivation revealed by single-cell allele-specific expression in human fibroblasts. Proc. Natl. Acad. Sci. USA *115*, 13015–13020. https://doi.org/10.1073/pnas.1806811115.
17. Wainer Katsir, K., and Linial, M. (2019). Human genes escaping X-inactivation revealed by single cell expression data. BMC Genom. *20*, 201. https://doi.org/10.1186/s12864-019-5507-6.
18. Sauteraud, R., Stahl, J.M., James, J., Englebright, M., Chen, F., Zhan, X., Carrel, L., and Liu, D.J. (2021). Inferring genes that escape X-Chromosome inactivation reveals important contribution of variable escape genes to sex-biased diseases. Genome Res. *31*, 1629–1637. https://doi.org/10.1101/gr.275677.121.
19. Sudbrak, R., Wieczorek, G., Nuber, U.A., Mann, W., Kirchner, R., Erdogan, F., Brown, C.J., Wöhrle, D., Sterk, P., Kalscheuer, V.M., et al. (2001). X chromosome-specific cDNA arrays: identification of genes that escape from X-inactivation and other applications. Hum. Mol. Genet. *10*, 77–83.
20. Craig, I.W., Mill, J., Craig, G.M., Loat, C., and Schalkwyk, L.C. (2004). Application of microarrays to the analysis of the inactivation status of human X-linked genes expressed in lymphocytes. Eur. J. Hum. Genet. *12*, 639–646. https://doi.org/10.1038/sj.ejhg.5201212.
21. Talebizadeh, Z., Simon, S.D., and Butler, M.G. (2006). X chromosome gene expression in human tissues: male and female comparisons. Genomics *88*, 675–681. https://doi.org/10.1016/j.ygeno.2006.07.016.
22. Johnston, C.M., Lovell, F.L., Leongamornlert, D.A., Stranger, B.E., Dermitzakis, E.T., and Ross, M.T. (2008). Large-scale population study of human cell lines indicates that dosage compensation is virtually complete. PLoS Genet. *4*, e9. https://doi.org/10.1371/journal.pgen.0040009.
23. Trolle, C., Nielsen, M.M., Skakkebæk, A., Lamy, P., Vang, S., Hedegaard, J., Nordentoft, I., Ørntoft, T.F., Pedersen, J.S., and Gravholt, C.H. (2016). Widespread DNA hypomethylation and differential gene expression in Turner syndrome. Sci. Rep. *6*, 34220–34214. https://doi.org/10.1038/srep34220.
24. Raznahan, A., Parikshak, N.N., Chandran, V., Blumenthal, J.D., Clasen, L.S., Alexander-Bloch, A.F., Zinn, A.R., Wangsa, D., Wise, J., Murphy, D.G.M., et al. (2018). Sex-chromosome dosage effects on gene expression in humans. Proc. Natl. Acad. Sci. USA *115*, 7398–7403. https://doi.org/10.1073/pnas.1802889115.
25. Zhang, X., Hong, D., Ma, S., Ward, T., Ho, M., Pattni, R., Duren, Z., Stankov, A., Bade Shrestha, S., Hallmayer, J., et al. (2020). Integrated functional genomic analyses of Klinefelter and Turner syndromes reveal global network effects of altered X chromosome dosage. Proc. Natl. Acad. Sci. USA *117*, 4864–4873. https://doi.org/10.1073/pnas.1910003117.
26. Nielsen, M.M., Trolle, C., Vang, S., Hornshøj, H., Skakkebaek, A., Hedegaard, J., Nordentoft, I., Pedersen, J.S., and Gravholt, C.H. (2020). Epigenetic and transcriptomic consequences of excess X-chromosome material in 47,XXX syndrome-A comparison with Turner syndrome and 46,XX females. Am. J. Med. Genet. C Semin. Med. Genet. *184*, 279–293. https://doi.org/10.1002/ajmg.c.31799.

CellPress
OPEN ACCESS

27. Skaletsky, H., Kuroda-Kawaguchi, T., Minx, P.J., Cordum, H.S., Hillier, L., Brown, L.G., Repping, S., Pyntikova, T., Ali, J., Bieri, T., et al. (2003). The male-specific region of the human Y chromosome is a mosaic of discrete sequence classes. Nature *423*, 825–837. https://doi.org/10.1038/nature01722.

28. Ross, M.T., Grafham, D.V., Coffey, A.J., Scherer, S., McLay, K., Muzny, D., Platzer, M., Howell, G.R., Burrows, C., Bird, C.P., et al. (2005). The DNA sequence of the human X chromosome. Nature *434*, 325–337. https://doi.org/10.1038/nature03440.

29. Bellott, D.W., Hughes, J.F., Skaletsky, H., Brown, L.G., Pyntikova, T., Cho, T.-J., Koutseva, N., Zaghlul, S., Graves, T., Rock, S., et al. (2014). Mammalian Y chromosomes retain widely expressed dosage-sensitive regulators. Nature *508*, 494–499. https://doi.org/10.1038/nature13206.

30. Brown, C.J., Ballabio, A., Rupert, J.L., Lafreniere, R.G., Grompe, M., Tonlorenzi, R., and Willard, H.F. (1991). A gene from the region of the human X inactivation centre is expressed exclusively from the inactive X chromosome. Nature *349*, 38–44. https://doi.org/10.1038/349038a0.

31. Penny, G.D., Kay, G.F., Sheardown, S.A., Rastan, S., and Brockdorff, N. (1996). Requirement for Xist in X chromosome inactivation. Nature *379*, 131–137. https://doi.org/10.1038/379131a0.

32. Ciccodicola, A., D'Esposito, M., Esposito, T., Gianfrancesco, F., Migliaccio, C., Miano, M.G., Matarazzo, M.R., Vacca, M., Franzè, A., Cuccurese, M., et al. (2000). Differentially regulated and evolved genes in the fully sequenced Xq/Yq pseudoautosomal region. Hum. Mol. Genet. *9*, 395–401. https://doi.org/10.1093/hmg/9.3.395.

33. Mégarbané, A., Ravel, A., Mircher, C., Sturtz, F., Grattau, Y., Rethoré, M.O., Delabar, J.M., and Mobley, W.C. (2009). The 50th anniversary of the discovery of trisomy 21: the past, present, and future of research and treatment of Down syndrome. Genet. Med. *11*, 611–616. https://doi.org/10.1097/GIM.0b013e3181b2e34c.

34. Sullivan, K.D., Lewis, H.C., Hill, A.A., Pandey, A., Jackson, L.P., Cabral, J.M., Smith, K.P., Liggett, L.A., Gomez, E.B., Galbraith, M.D., et al. (2016). Trisomy 21 consistently activates the interferon response. Elife *5*, e16220. https://doi.org/10.7554/eLife.16220.

35. Karczewski, K.J., Francioli, L.C., Tiao, G., Cummings, B.B., Alföldi, J., Wang, Q., Collins, R.L., Laricchia, K.M., Ganna, A., Birnbaum, D.P., et al. (2020). The mutational constraint spectrum quantified from variation in 141,456 humans. Nature *581*, 434–443. https://doi.org/10.1038/s41586-020-2308-7.

36. Petrovski, S., Wang, Q., Heinzen, E.L., Allen, A.S., and Goldstein, D.B. (2013). Genic intolerance to functional variation and the interpretation of personal genomes. PLoS Genet. *9*, e1003709. https://doi.org/10.1371/journal.pgen.1003709.

37. Huang, N., Lee, I., Marcotte, E.M., and Hurles, M.E. (2010). Characterising and predicting haploinsufficiency in the human genome. PLoS Genet. *6*, e1001154. https://doi.org/10.1371/journal.pgen.1001154.

38. Friedman, R.C., Farh, K.K.H., Burge, C.B., and Bartel, D.P. (2009). Most mammalian mRNAs are conserved targets of microRNAs. Genome Res. *19*, 92–105. https://doi.org/10.1101/gr.082701.108.

39. Bartel, D.P. (2009). MicroRNAs: target recognition and regulatory functions. Cell *136*, 215–233. https://doi.org/10.1016/j.cell.2009.01.002.

40. Naqvi, S., Bellott, D.W., Lin, K.S., and Page, D.C. (2018). Conserved microRNA targeting reveals preexisting gene dosage sensitivities that shaped amniote sex chromosome evolution. Genome Res. *28*, 474–483. https://doi.org/10.1101/gr.230433.117.

41. Ogata, T., and Matsuo, N. (1993). Sex chromosome aberrations and stature: deduction of the principal factors involved in the determination of adult height. Hum. Genet. *91*, 551–562. https://doi.org/10.1007/BF00205079.

42. Rao, E., Weiss, B., Fukami, M., Rump, A., Niesler, B., Mertz, A., Muroya, K., Binder, G., Kirsch, S., Winkelmann, M., et al. (1997). Pseudoautosomal deletions encompassing a novel homeobox gene cause growth failure in idiopathic short stature and Turner syndrome. Nat. Genet. *16*, 54–63. https://doi.org/10.1038/ng0597-54.

43. Clement-Jones, M., Schiller, S., Rao, E., Blaschke, R.J., Zuniga, A., Zeller, R., Robson, S.C., Binder, G., Glass, I., Strachan, T., et al. (2000). The short stature homeobox gene SHOX is involved in skeletal abnormalities in Turner syndrome. Hum. Mol. Genet. *9*, 695–702. https://doi.org/10.1093/hmg/9.5.695.

44. Ottesen, A.M., Aksglaede, L., Garn, I., Tartaglia, N., Tassone, F., Gravholt, C.H., Bojesen, A., Sørensen, K., Jørgensen, N., Rajpert-De Meyts, E., et al. (2010). Increased number of sex chromosomes affects height in a nonlinear fashion: a study of 305 patients with sex chromosome aneuploidy. Am. J. Med. Genet. *152A*, 1206–1212. https://doi.org/10.1002/ajmg.a.33334.

45. Fukami, M., Seki, A., and Ogata, T. (2016). SHOX haploinsufficiency as a cause of syndromic and nonsyndromic short stature. Mol. Syndromol. *7*, 3–11. https://doi.org/10.1159/000444596.

46. Galan-Caridad, J.M., Harel, S., Arenzana, T.L., Hou, Z.E., Doetsch, F.K., Mirny, L.A., and Reizis, B. (2007). Zfx controls the self-renewal of embryonic and hematopoietic stem cells. Cell *129*, 345–357. https://doi.org/10.1016/j.cell.2007.03.014.

47. Zhou, Y., Su, Z., Huang, Y., Sun, T., Chen, S., Wu, T., Chen, G., Xie, X., Li, B., and Du, Z. (2011). The Zfx gene is expressed in human gliomas and is important in the proliferation and apoptosis of the human malignant glioma cell line U251. J. Exp. Clin. Cancer Res. *30*, 114. https://doi.org/10.1186/1756-9966-30-114.

48. Fang, Q., Fu, W.H., Yang, J., Li, X., Zhou, Z.S., Chen, Z.W., and Pan, J.H. (2014). Knockdown of ZFX suppresses renal carcinoma cell growth and induces apoptosis. Cancer Genet. *207*, 461–466. https://doi.org/10.1016/j.cancergen.2014.08.007.

49. Carrel, L., and Willard, H.F. (1999). Heterogeneous gene expression from the inactive X chromosome: an X-linked gene that escapes X inactivation in some human cell lines but is inactivated in others. Proc. Natl. Acad. Sci. USA *96*, 7364–7369. https://doi.org/10.1073/pnas.96.13.7364.

50. Okamoto, I., Nakamura, T., Sasaki, K., Yabuta, Y., Iwatani, C., Tsuchiya, H., Nakamura, S.I., Ema, M., Yamamoto, T., and Saitou, M. (2021). The X chromosome dosage compensation program during the development of cynomolgus monkeys. Science *374*, eabd8887. https://doi.org/10.1126/science.abd8887.

51. Ohno, S. (1967). Sex Chromosomes and Sex-Linked Genes (Springer Berlin Heidelberg). https://doi.org/10.1007/978-3-642-88178-7.

52. Godfrey, A.K., Naqvi, S., Chmátal, L., Chick, J.M., Mitchell, R.N., Gygi, S.P., Skaletsky, H., and Page, D.C. (2020). Quantitative analysis of Y-Chromosome gene expression across 36 human tissues. Genome Res. *30*, 860–873. https://doi.org/10.1101/gr.261248.120.

53. GTEx Consortium; Laboratory, Data Analysis &Coordinating Center LDACC—Analysis Working Group; Statistical Methods groups—Analysis Working Group; Enhancing GTEx eGTEx groups; NIH Common Fund; NIH/NCI; NIH/NHGRI; NIH/NIMH; NIH/NIDA; Biospecimen Collection Source Site—NDRI (2017). Genetic effects on gene expression across human tissues. Nature *550*, 204–213. https://doi.org/10.1038/nature24277.

54. Bray, N.L., Pimentel, H., Melsted, P., and Pachter, L. (2016). Near-optimal probabilistic RNA-seq quantification. Nat. Biotechnol. *34*, 525–527. https://doi.org/10.1038/nbt.3519.

55. Love, M.I., Huber, W., and Anders, S. (2014). Moderated estimation of fold change and dispersion for RNA-seq data with DESeq2. Genome Biol. *15*, 550. https://doi.org/10.1186/s13059-014-0550-8.

56. Pimentel, H., Bray, N.L., Puente, S., Melsted, P., and Pachter, L. (2017). Differential analysis of RNA-seq incorporating quantification uncertainty. Nat. Methods *14*, 687–690. https://doi.org/10.1038/nmeth.4324.

57. Quinlan, A.R., and Hall, I.M. (2010). BEDTools: a flexible suite of utilities for comparing genomic features. Bioinformatics *26*, 841–842. https://doi.org/10.1093/bioinformatics/btq033.

58. Robinson, J.T., Thorvaldsdóttir, H., Winckler, W., Guttman, M., Lander, E.S., Getz, G., and Mesirov, J.P. (2011). Integrative genomics viewer. Nat. Biotechnol. *29*, 24–26. https://doi.org/10.1038/nbt.1754.

59. Vangipuram, M., Ting, D., Kim, S., Diaz, R., and Schüle, B. (2013). Skin punch biopsy explant culture for derivation of primary human fibroblasts. JoVE, e3779. https://doi.org/10.3791/3779.

60. Naqvi, S., Godfrey, A.K., Hughes, J.F., Goodheart, M.L., Mitchell, R.N., and Page, D.C. (2019). Conservation, acquisition, and functional impact of sex-biased gene expression in mammals. Science *365*, eaaw7317. https://doi.org/10.1126/science.aaw7317.

61. Pruitt, K.D., Harrow, J., Harte, R.A., Wallin, C., Diekhans, M., Maglott, D.R., Searle, S., Farrell, C.M., Loveland, J.E., Ruef, B.J., et al. (2009). The consensus coding sequence (CCDS) project: identifying a common protein-coding gene set for the human and mouse genomes. Genome Res. *19*, 1316–1323. https://doi.org/10.1101/gr.080531.108.

62. Soneson, C., Love, M.I., and Robinson, M.D. (2015). Differential analyses for RNA-seq: transcript-level estimates improve gene-level inferences. F1000Res. *4*, 1521. https://doi.org/10.12688/f1000research.7563.2.

63. Bellott, D.W., and Page, D.C. (2021). Dosage-sensitive functions in embryonic development drove the survival of genes on sex-specific chromosomes in snakes, birds, and mammals. Genome Res. *31*, 198–210. https://doi.org/10.1101/gr.268516.120.

64. Mueller, J.L., Skaletsky, H., Brown, L.G., Zaghlul, S., Rock, S., Graves, T., Auger, K., Warren, W.C., Wilson, R.K., and Page, D.C. (2013). Independent specialization of the human and mouse X chromosomes for the male germ line. Nat. Genet. *45*, 1083–1087. https://doi.org/10.1038/ng.2705.

65. Jackson, E.K., Bellott, D.W., Cho, T.J., Skaletsky, H., Hughes, J.F., Pyntikova, T., and Page, D.C. (2021). Large palindromes on the primate X Chromosome are preserved by natural selection. Genome Res. *31*, 1337–1352. https://doi.org/10.1101/gr.275188.120.

66. McKusick-Nathans Institute of Genetic Medicine. (2022). Online Mendelian Inheritance in Man (OMIM). https://omim.org/.

67. R Development Core Team. (2020). R: A Language and Environment for Statistical Computing (R Foundation for Statistical Computing).

STAR★METHODS

KEY RESOURCES TABLE

REAGENT or RESOURCE	SOURCE	IDENTIFIER
**Experimental models: Cell lines**		
Lymphoblastoid cell lines and primary fibroblast cell cultures	This paper	N/A
Lymphoblastoid cell lines	Colorado Children's Hospital Biobank	N/A
Lymphoblastoid cell lines and primary fibroblast cell cultures	Coriell Cell Repository	See Table S1
EBV-producing lymphoblasts	Coriell Cell Repository	B95-8 (RRID:CVCL_1953)
**Chemicals, peptides, and recombinant proteins**		
Percoll	Cytiva	Cat# 17-0891-01
RPMI 1640	Gibco	Cat# 31800-089
HEPES	SAFC	Cat# RES6008H-A702X
FBS	Hyclone	Cat# SH30071
Amphotericin B	Gibco	Cat# 15290-018
Gentamicin	Gibco	Cat# 15710-072
Penicillin-Streptomycin	Lonza	Cat# 11140-076
Cyclosporine	LC Laboratories	Cat# C-6000
DMEM/F12	Gibco	Cat# 11320-033
DMEM High Glucose	Gibco	Cat# 11960-069
L-Glutamine	MP Biomedicals	Cat# IC10180683
MEM Non-essential amino acids	Gibco	Cat# 15140-163
Gelatin	Sigma	Cat# G2500
TRIzol	ThermoFisher	Cat# 15596026
RNAprotect Cell Reagent	Qiagen	Cat# 76526
**Critical commercial assays**		
Vacutainer ACD Tubes	BD Biosciences	Cat# 364606
MycoAlert Kit	Lonza	Cat# LT07-318
SapphireAmp Fast PCR Master Mix	Takara	Cat# RR350A
RNeasy Plus Mini Kit	Qiagen	Cat# 74134
QIAshredder Columns	Qiagen	Cat# 79654
Qubit RNA HS Assay Kit	ThermoFisher	Cat# Q32855
Fragment Analyzer RNA Kit	Agilent	Cat# DNF-471
HS NGS Fragment Kit	Agilent	Cat # DNF-474
TruSeq RNA Library Preparation Kit v2	Illumina	Cat# RS-122-2001
KAPA mRNA Hyper-Prep Kit	Roche	Cat# KK8581
PippinHT 2% Agarose Gel Cassettes	Sage Science	Cat# HTC2010
**Deposited data**		
Raw, de-identified RNA-seq data	This paper	dbGaP: phs002481.v2.p1
Processed data	This paper	https://doi.org/10.5281/zenodo.7504743
Custom GENCODE v24 transcriptome annotation	Godfrey et al., 2020[52]	https://doi.org/10.5281/zenodo.3627233
GTEx Expression Data	GTEx Consortium et al., 2017[53]	https://gtexportal.org
CTCF ChIP-seq GM12878	ENCODE	ENCFF852CRG
CTCF ChIP-seq GM12864	ENCODE	ENCFF593YIG
CTCF ChIP-seq AG09309	ENCODE	ENCFF640EZJ
CTCF ChIP-seq AG10803	ENCODE	ENCFF694SQA

(Continued on next page)

REAGENT or RESOURCE	SOURCE	IDENTIFIER
Continued		
CTCF ChIA-PET GM12878	ENCODE	ENCFF80PGS, ENCFF847QOE
LOEUF scores and homozygous LoF gene list	Karczewski et al., 2020[35]	https://gnomad.broadinstitute.org/
RVIS scores	Petrovski et al., 2013[36]	http://genic-intolerance.org/data/RVIS_Unpublished_ExAC_May2015.txt
P_{ct} scores	Friedman et al., 2009[38]	N/A
pHI scores	Huang et al., 2010[37]	N/A
X chromosome inactivation consensus calls	Balaton et al., 2015[9]	N/A
Microarray dataset of sex chromosome aneuploidy samples	Raznahan et al., 2018[24]	N/A
Allelic expression data in fibroblasts and hybrid cell lines	Carrel et al., 2005[13]	N/A
Allelic expression data in LCLs and fibroblasts from paired genomic and cDNA SNP-chips	Cotton et al., 2013[14]	N/A
Allelic expression data from bulk and single LCLs in GTEx	Tukiainen et al., 2017[15]	N/A
Allelic expression data in single fibroblasts	Garieri et al., 2018[16]	N/A
Allelic expression data in bulk LCLs	Sauteraud et al., 2021[18]	N/A
Phenotype associations with Chr X genes	OMIM	https://www.omim.org/
**Oligonucleotides**		
ERCC RNA Sike-In Mix	Invitrogen	Cat#: 4456740
Mycoplasma primer, F: CTT CWT CGA CTT YCA GAC CCA AGG CAT	This paper	N/A
Mycoplasma primer, R: ACA CCA TGG GAG YTG GTA AT	This paper	N/A
hGAPDH primer, F: TGT CGC TGT TGA AGT CAG AGG AGA	This paper	N/A
hGAPDH primer, R: AGA ACA TCA TCC CTG CCT CTA CTG	This paper	N/A
**Software and algorithms**		
Custom code to process RNA-seq data and generate figures	This paper	https://doi.org/10.5281/zenodo.7504743
R v3.6.3	The R Foundation	https://www.r-project.org
Kallisto v0.42.5	Bray et al., 2016[54]	https://pachterlab.github.io/kallisto/
DESeq2 v1.26.0	Love et al., 2014[55]	https://bioconductor.org/packages/release/bioc/html/DESeq2.html
Sleuth v0.30.0	Pimentel et al., 2017[56]	https://pachterlab.github.io/sleuth/
BEDTools v2.26.0	Quinlan et al., 2010[57]	https://bedtools.readthedocs.io/
IGV	Robinson et al., 2011[58]	https://software.broadinstitute.org/software/igv/
Best Practices workflow for identifying short variants in RNA-seq data	Broad Institute	https://gatk.broadinstitute.org/hc/en-us/articles/360035531192-RNAseq-short-variant-discovery-SNPs-Indels-
Illustrator	Adobe	https://www.adobe.com/products/illustrator.html
**Other**		
QuBit 4 Fluorometer	ThermoFisher	N/A
5200 Fragment Analyzer System	Agilent	N/A
PippinHT system	Sage Sciences	N/A
HiSeq 2500	Illumina	N/A
NovaSeq 6000	Illumina	N/A

RESOURCE AVAILABILITY

Lead contact

Further information and request for resources and reagents should be directed to and will be fulfilled by lead contact, David C. Page (dcpage@wi.mit.edu).

Materials availability

Cell lines are available upon request to the lead contact.

Data and code availability

- Raw, de-identified RNA-sequencing data from human cell cultures has been deposited to dbGaP under accession number phs002481.v2.p1, and processed data has been deposited at Zenodo under accession number https://doi.org/10.5281/zenodo.7504743.
- This paper analyzes existing, publicly available data. Accession numbers for these datasets are listed in the key resources table.
- Original code has been deposited at Zenodo under accession number https://doi.org/10.5281/zenodo.7504743 and is publicly available as of the date of publication.
- Any additional information required to reanalyze the data reported in this paper is available from the lead contact upon request.

EXPERIMENTAL MODEL AND SUBJECT DETAILS

Human subjects

Adults (18+ years of age) with sex chromosome aneuploidies or euploid controls were recruited through an IRB-approved study at the NIH Clinical Center (12-HG-0181) and Whitehead Institute/MIT (Protocol #1706013503). Informed consent was obtained from all study participants. Individuals with a previous karyotype showing non-mosaic sex chromosome aneuploidy were included in the study. From these individuals, blood samples and skin biopsies were collected at the NIH Clinical Center and shipped to the Page lab for derivation of cell lines. In addition, blood samples from individuals with sex chromosome aneuploidies, and euploid family members, ranging in age from 4-44 years were contributed by the Focus Foundation. Additional LCLs and fibroblast cultures were obtained from the Colorado Children's Hospital Biobank and Coriell Research Institute, and cultured in the Page laboratory for at least two passages prior to collection for RNA-sequencing. Karyotyping of peripheral blood and fibroblast cell cultures was performed at the National Human Genome Research Institute Cytogenetics and Microscopy Core. To reduce the impact of sex chromosome mosaicism on our sex chromosome aneuploidy analysis, we excluded individuals with >15% mosaicism for other karyotypes. Metadata for cell lines represented in the RNA-sequencing dataset are provided in Table S1.

METHOD DETAILS

Cell culture

Lymphoblastoid cell lines

Blood was collected in BD Vacutainer ACD tubes and shipped at room temperature to the Page Lab for processing 1-3 days after collection. The buffy coat was resolved by centrifuging blood at 3300 rpm for 10 min, transferred to a new tube with PBS, and subjected to density gradient centrifugation in 50% Percoll (Cytiva) at 3300 rpm for 10 min. Lymphocytes were transferred to a new tube and washed twice with PBS. Lymphocytes were resuspended in 3 mL complete RPMI medium (RPMI 1640 (Gibco), 25mM HEPES (SAFC), 15% FBS (Hyclone), Fungizone (Amphotericin B, Gibco), Gentamicin (Gibco), Penicillin/Streptomycin (Lonza), pH 7.2) per tube of blood and transferred to a T25 flask, supplemented with 0.25mL EBV (produced by B95-8 marmoset lymphoblasts), and 0.2 mL of 1 mg/mL cyclosporine (LC Laboratories). They were incubated for one week at 37°C, fed 1-2 mL complete RPMI, and incubated for another week at 37°C. Once the media began to turn yellow (acidified), cultures were "half-fed" by removing half of the media and replacing it with double the volume. When cultures reached 15 mL, they were transferred to T75 flasks, and gradually expanded to 30 mL, while maintaining a concentration of <1 million cells/mL to ensure viability. Cells were viably frozen for future use by mixing with freezing media (LCL culture media + 5% DMSO), 1 million cells per vial. Cells were also preserved for RNA, DNA, and protein extraction (see below).

Primary fibroblast cultures

Our protocol for generating primary skin fibroblast cultures from a skin biopsy is based on Vangipuram et al.[59] From adults (18+ years of age) at the NIH Clinical Center we obtained two 4-mm skin punch biopsies from the upper arm, which were immediately placed into a 15 mL conical tube with 10 mL of media (DMEM/F12 (Gibco), 20% FBS, and 100 IU/mL Penicillin-Streptomycin. Tubes were shipped to the Page lab overnight on ice for processing. Each biopsy was used to generate a separate skin fibroblast culture. Biopsies were cut into 18 pieces of equal size and placed 3/well in gelatinized 6-well plates with 1 mL media (High Glucose DMEM (Gibco),

20% FBS, L-Glutamine (MP Biomedicals), MEM Non-Essential Amino Acids (Gibco), 100 IU/mL Penicillin/Streptomycin (Lonza)). Plates were gelatinized by incubating 1 mL sterile 0.1% gelatin (Sigma) solution per well for 30 min at room temperature.

Plates were incubated for 1 week at 37°C without disturbance to allow biopsies to attach to the plate and begin to grow out. During week 2, we added 200 μL of fresh media per well every 2-3 days, being careful not to disturb the biopsies. The following week (week 3), we aspirated the media and replaced with 1 mL fresh media per well every 2-3 days. During week 4, we aspirated the media and replaced with 2 mL fresh media per well every 2-3 days. At this point, the fibroblasts generally reached the edges of the wells and were expanded to two T75 gelatinized flasks per 6 well plate. After two days, we combined the cells from the two T75 flasks and split them to three T175 gelatinized flasks. After two days, cells were viably frozen with 1 million cells per vial in freezing media (fibroblast culture media + 5% DMSO). Cells were also preserved for RNA extraction (see below). During optimization of the protocol, cell culture purity was confirmed by immunofluorescence of SERPINH1, a fibroblast marker.

Cell collection for subsequent analysis

Cells were collected when LCL cultures reached 30mL, and fibroblasts were ~80% confluent in three T175 plates. All cell counting was performed using the Countess II cell counter (Life Technologies) and Trypan Blue exclusion. Cultures with >85% cell viability were used in subsequent experiments. To preserve cells for subsequent RNA extraction, 1 million cells were washed in PBS, pelleted, and resuspended in 500 μL TRIzol (Invitrogen) or 200 μL RNAprotect Cell Reagent (Qiagen). Cell suspensions were then frozen at −80°C. Cell cultures were maintained at low passage number; RNA-sequencing experiments were performed on samples at or below passage 4.

Periodically, and on each passage used for experiments, cell cultures were confirmed negative for mycoplasma contamination using either the MycoAlert Kit (Lonza) following the manufacturer's instructions, or PCR using SapphireAmp Fast PCR Master Mix (Takara) and the following primers:

Myco2(cb): 5′ CTTCWTCGACTTYCAGACCCAAGGCAT-3′

Myco11(cb): 5′ ACACCATGGGAGYTGGTAAT-3′

PCR for *GAPDH* was performed on the same sample, using the following primers:

hGAPDH-F: TGT CGC TGT TGA AGT CAG AGG AGA

hGAPDH-R: AGA ACA TCA TCC CTG CCT CTA CTG.

Known mycoplasma positive and negative samples were used as a reference.

RNA extraction, library preparation, and sequencing

RNA was extracted from 1 million cells per experiment using the RNeasy Plus Mini Kit (Qiagen) following the manufacturer's instructions, with the following modifications: Cells in RNAprotect Cell Reagent were thawed on ice, pelleted, and lysed in buffer RLT supplemented with 10μL β-mercaptoethanol per mL. For most samples, ERCC control RNAs were added to the lysate based on the number of cells: 10μL of 1:100 dilution of ERCC control RNAs was added per 1 million cells. The lysate was then homogenized using QIAshredder columns (Qiagen), and transferred to a gDNA eliminator column. All subsequent optional steps in the protocol were performed, and RNA was eluted in 30 μL RNase-free water. RNA levels were measured using a Qubit fluorometer and the Qubit RNA HS Assay Kit (ThermoFisher). Before we switched to the per-cell spike-in protocol, we prepared 18 samples in which ERCC control RNAs were added based on amount of RNA after isolation: 2 μL of a 1:100 dilution of ERCC control RNAs was added per 1 μg of RNA. These samples are: #2237, 2245, 6312, 711, 4032, 706, 3429, 3430, 3442, 2690, 2703, 3107, 5297, 5566, 5755, 6029, 2547, and 525. RNA quality control was performed using the 5200 Fragment Analyzer System (Agilent); we consistently purified high-quality RNA with RNA integrity numbers (RIN) near 10. We randomized the samples by karyotype into batches for RNA extraction, library preparation, and sequencing.

RNA sequencing libraries were prepared using the TruSeq RNA Library Preparation Kit v2 (Illumina) with modifications as detailed in Naqvi et al,[60] or using the KAPA mRNA Hyper-Prep Kit V2 (Roche). In both cases, libraries were size selected using the PippinHT system (Sage Science) and 2% agarose gels with a capture window of 300-600 bp. Paired-end 100x100 bp sequencing was performed on a HiSeq 2500 or NovaSeq 6000 (Illumina). Table S1 lists the library preparation kit and sequencing platform for each sample.

RNA-seq data processing and analysis

All analyses were performed using human genome build hg38, and a custom version of the comprehensive GENCODE v24 transcriptome annotation.[52] This annotation represents the union of the "GENCODE Basic" annotation *and* transcripts recognized by the Consensus Coding Sequence project.[61] Importantly, the GENCODE annotation lists the PAR gene annotations twice – once on Chr X and once on Chr Y– which complicates analysis. We removed these annotations from Chr Y so the PAR genes are only listed once in our annotation, on Chr X. To analyze samples in which ERCC spike-ins were added, we merged our custom transcript annotation with the ERCC Control annotation.

Reads were pseudoaligned to the transcriptome annotation, and expression levels of each transcript were estimated using kallisto software v0.42.5.[54] We included the "–bias" flag to correct for sequence bias. The resulting count data (abundance.tsv file) were imported into R with the tximport package v1.14.0[62] for normalization using DESeq2 v1.26.0.[55] For downstream analysis, we used only protein-coding genes (as annotated in ensembl v104) with the following exceptions: we included genes annotated as pseudogenes on Chr Y that are members of X-Y pairs (*TXLNGY, PRKY*) and well-characterized long non-coding RNAs (lncRNAs) involved in X-inactivation or other processes (*XIST, JPX, FTX, XACT, FIRRE, TSIX).* We annotated genes distal to *XG*, which spans the

pseudoautosomal boundary on Xp and is truncated on Chr Y, as part of PAR1 - 15 genes in total. PAR2 comprised the four most distal genes on Xq and Yq. Annotations of non-pseudoautosomal region of the X (NPX) genes with homologs on the non-pseudoautosomal region of the Y (NPY) were derived from Bellott et al.[63] 224 protein-coding genes on Chr 21 (ensembl v104) were used as a starting point for our analyses. We excluded 21 annotated genes in several regions with high homology between the long and short arms of Chr 21 because the assembly was not fully validated in these regions (https://www.ncbi.nlm.nih.gov/grc/human/issues?filters=chr:21).

Identifying genes affected by changes in chr X, Y, or 21 copy number

We first defined lists of expressed NPX, NPY, PAR, or Chr 21 genes as those with median TPM of at least 1 in 46,XX or 46,XY samples. To ensure that no genes with robust expression were excluded, we also analyzed LCL and fibroblast expression data from GTEx,[53] and included several genes that were just below our TPM cutoff but had median TPM of at least 1 in those datasets.

For each expressed NPX, NPY, or PAR gene we performed linear modeling using the lm() function in R. These calculations suppose that each additional chromosome adds a consistent and equal increment to the total expression level of the gene in question.

For NPX and PAR genes we used the following equation:

$$E = \beta_0 + \beta_X(\#chrXi) + \beta_Y(\#chrY) + \beta_B(batch) + \varepsilon$$

E represents the expression (read counts) per gene, β_0 represents the intercept, β_X and β_Y are the coefficients of the effect of additional copies of Chr Xi or Y, respectively, and $\in$ is an error term. For this equation, the intercept represents the 45,X samples.

For NPY genes we employed the following equation, analyzing only those samples with one or more copies of Chr Y:

$$E = \beta_0 + \beta_X(\#chrXi) + \beta_Y(\#chrY - 1) + \beta_B(batch) + \varepsilon$$

For this equation, the intercept represents the 46,XY samples.

For Chr 21 genes we employed the following equation, analyzing only those samples with 46,XX; 46,XX; 47,XY,+21; or 47,XX,+21 karyotypes:

$$E = \beta_0 + \beta_{21}(\#chr21 - 2) + \beta_{Sex}(Sex) + \beta_B(batch) + \varepsilon$$

β_{21} and β_{Sex} are the coefficients of the effect of an additional copy of Chr 21 and sex (XY vs XX), respectively. For this equation, the intercept represents the 46,XX samples.

The resulting p values were adjusted for multiple hypothesis testing using the p.adjust() function in R, specifying the Benjamini Hochberg method. Genes with a false discovery rate (FDR) < 0.05 were considered significant. To compute the normalized expression change per Chr Xi (ΔE_X) or Y (ΔE_Y), we divided the coefficient of interest (β_X or β_Y) by the average intercept across batches, which corresponds to the baseline expression of the gene in samples with only one X chromosome (for NPX and PAR genes) or one Y chromosome (in the case of NPY genes). For Chr 21, we computed ΔE_{21} by dividing the coefficient (β_{21}) by the average intercept across batches divided by two to obtain the average expression from one copy of Chr 21.

$$\Delta E_X = \frac{\beta_X}{\beta_0} \quad \Delta E_Y = \frac{\beta_Y}{\beta_0} \quad \Delta E_{21} = \frac{\beta_{21}}{\beta_0/2}$$

In the case of *XIST*, which is only expressed when two or more copies of Chr X are present, we used the following equations:

$$\Delta E_X = \frac{\beta_X}{\beta_0 + \beta_X} \quad \Delta E_Y = \frac{\beta_Y}{\beta_0 + \beta_X}$$

We calculated the standard error (SE) of ΔE_X, ΔE_Y, and ΔE_{21} using the following equations:

$$S_{\Delta E_X} = \sqrt{\frac{\beta_X{}^2}{\beta_0{}^2}\left[\frac{S_{\beta_X}{}^2}{\beta_X{}^2} + \frac{S_{\beta_0}{}^2}{\beta_0{}^2}\right]} \quad S_{\Delta E_Y} = \sqrt{\frac{\beta_Y{}^2}{\beta_0{}^2}\left[\frac{S_{\beta_Y}{}^2}{\beta_Y{}^2} + \frac{S_{\beta_0}{}^2}{\beta_0{}^2}\right]} \quad S_{\Delta 21} = \sqrt{\frac{\beta_{21}{}^2}{(\beta_0/2)^2}\left[\frac{S_{\beta_{21}}{}^2}{\beta_{21}{}^2} + \frac{S_{\beta_0}{}^2}{(\beta_0/2)^2}\right]}$$

To confirm the validity of our approach, we used bootstrapping to sample our dataset with replacement 1000 times and obtained similar results. *BEX1* was removed from downstream analyses in fibroblasts because two samples (one 45,X and one 49,XXXXY) had high expression values for this gene resulting in >25 times higher error values for ΔE_X and ΔE_Y compared to all other genes.

Saturation analysis for sex chromosome-encoded genes

For LCLs and fibroblasts, size-*n* subsets of available RNA-seq libraries were sampled randomly without replacement, 100 times for each sample size, *n*. After confirming that the model matrix would be full rank in each sampling (for example, that samples would not all be of the same karyotype or batch), we performed linear modeling on NPX, PAR, NPY, and Chr 21 genes as described above to identify genes whose expression changes significantly (FDR < 0.05) with copy number of Chr X, Y or 21.

Assessing linearity of sex-chromosome gene expression changes

To assess whether sex-chromosome gene expression changed linearly (i.e., by a fixed amount) with additional X or Y chromosomes, their expression levels across the LCL or fibroblast samples were fit by non-linear least squares to the power curves shown below, using the "nlsLM" function from the R package "minpack.lm".

NPX genes:

$$y_j^* = 1 + b(xcount_j - 1)^a, \text{where } y_j^* = \frac{y_j}{y_k}$$

PAR genes:

$$y_j^* = 1 + b(xcount_j + ycount_j - 1)^a, \text{where } y_j^* = \frac{y_j}{y_k}$$

NPY genes:

$$y_j^* = 1 + b(ycount_j - 1)^a, \text{where } y_j^* = \frac{y_j}{y_k}$$

In each of the equations above, y_j^* is the normalized RNA-seq read count for a given gene in sample j, given by the raw read count in sample j divided by the average read count in the set of samples $S_{\{\cdot\}}$ with only one chromosome of the relevant type: for NPX genes, 1 copy of Chr X (and any number of Y chromosomes); for PAR genes, 45,X samples; for NPY genes, 1 copy of Chr Y (and any number of X chromosomes). $b = 0.5$ and $a = 1$ were used as initial parameter values. Fitted values of $a \approx 1$ indicate a linear relationship between expression and sex-chromosome count. Fitted values of $a \approx 0$ or $b \approx 0$ indicate no change in expression with X or Y count.

ΔE_X calculations in samples with 0 Y chromosomes (females) and 1 Y chromosome (males)

We took subsets of the samples with either zero Y chromosomes (females) and with one Y chromosome (males) and performed the same linear modeling and ΔE_X calculations as above. We removed *MAP7D2* in female LCLs, *IL13RA2* in female fibroblasts, and *FHL1* in male fibroblasts because their error values (likely due to smaller sample size) were much higher than those of other genes. To compare the linear modeling results, we performed Pearson correlations between the results using all samples, and those from male-only or female-only samples.

Reanalysis of array data and comparison to RNA-seq data

A previous study performed gene expression analysis, using Illumina oligonucleotide BeadArrays, of LCLs from 68 individuals of the following karyotypes: 45,X; 46,XX; 46,XY; 47,XXX; 47,XXY; 47,XYY; and 48,XXYY.[24] Since this microarray dataset was generated from an independent set of samples, we sought to validate our results through a reanalysis of the data.

The raw data from the microarrays was not publicly available, but the authors provided us pre-processed data upon request, which we used to perform our analysis. To identify genes that cleared a minimum signal threshold to be considered expressed in the microarray data, we assessed the median signal in 46,XY samples for all Chr Y genes annotated on the microarray. We focused on Chr Y genes in this analysis because many are known to be expressed exclusively in testes, and therefore could provide us with an appropriate sense of the background signal expected for genes not expressed in LCLs. From this analysis, we concluded that a signal threshold of 111 would be appropriate for identifying expressed genes (Figure S9A). We used this threshold to identify 278 expressed Chr X genes (including PAR and NPX genes) in the microarray dataset. This was fewer than the 341 expressed Chr X genes identified in our LCL RNA-seq data, but more than double the 121 expressed Chr X genes reported in Raznahan et al. (Table S4 in Raznahan et al.).[24] This discrepancy could not be resolved by simply increasing the signal threshold in our analysis, as *TMSB4X,* one of the most highly expressed genes in LCLs, was excluded from the previously reported list of expressed genes.

Using our list of 278 expressed genes from the Raznahan et al. dataset, we analyzed the microarray signal values (in place of RNA-seq read counts) using linear models as a function of Xi copy number, controlling for Chr Y copy number. We calculated ΔE_X values from the microarray data and compared these to our RNA-seq dataset using a Pearson correlation, which revealed that the results were generally concordant (Figure S9B). For genes that were lowly expressed, however, ΔE_X values tended to be much lower in the microarray dataset, consistent with the higher sensitivity of RNA-seq data.

Isoform-specific analysis of RNA-seq data

After estimating counts for each transcript using kallisto software (as described above, with 100 bootstraps), we used sleuth v0.30.0 to normalize those transcript counts.[56] X chromosome transcripts were called expressed if their corresponding gene was on the list of expressed genes (above) and median transcript counts were >200. Linear regressions and ΔE_X calculations for transcripts were performed as for genes (above) to identify transcripts whose abundance changes significantly with additional copies of Chr X.

The following ENCODE datasets were used for visualization in IGV software[58] at the *UBA1* locus.

Assay	Cell type	Cell line	Karyotype	Accession
CTCF ChIP-seq	LCL	GM12878	46,XX	ENCFF644EEX
		GM12864	46,XY	ENCFF070FTG
	Fibroblast	AG09309	46,XX	ENCFF233THH
		AG10803	46,XY	ENCFF080HIA
CTCF ChIA-PET	LCL	GM12878	46,XX	ENCFF80PGS<br>ENCFF847QOE

Gene constraint analysis

To investigate sensitivity to a reduction in gene dosage, we used three metrics: LOEUF, RVIS, and pHI. We downloaded LOEUF (loss-of-function observed/expected upper fraction) scores from gnomAD (v2.1.1.lof_metris.by_gene.txt; https://gnomad.broadinstitute.org/), and only used scores with a minimum of 10 expected LoF variants. Updated RVIS (residual variation intolerance scores)[36] including the ExAC dataset were downloaded from http://genic-intolerance.org/data/RVIS_Unpublished_ExAC_May2015.txt. Updated probability of haploinsufficiency (pHI) scores[37] were downloaded from https://www.deciphergenomics.org/files/downloads/HI_Predictions_Version3.bed.gz. To complement these data, we obtained a list of genes with observed homozygous loss-of-function variants.[35] For sensitivity to an increase in gene dosage, we used the per-gene average probability of conserved miRNA targeting scores (P_{CT}).[38]

For each metric, we computed a percentile rank score, ranking from most-to least-constrained. Because several of the metrics calculated scores separately for autosomal (including PAR genes) and NPX genes, we ranked autosomal (and PAR) genes separately from NPX genes. All annotated genes, regardless of expression status in LCLs or fibroblasts, were included in the rankings, with the following exceptions: 1) NPX genes previously annotated as "ampliconic",[64,65] since constraint metrics cannot be accurately applied to these highly similar genes, and 2) genes with <2 annotations across all metrics.

To obtain an aggregate sense of a gene's expression constraint across multiple metrics, we calculated the average. Among NPX genes, we considered those with $|\Delta E_X \geq 0.1|$ (FDR < 0.05) to be most likely to contribute to phenotypes mediated by Xi copy number, prioritizing the top ten genes by the average gene-constraint metric. For PAR1 genes, we prioritized genes with an average gene constraint percentile ranking of at least 50%. To assess the phenotypic roles of highly constrained genes, we annotated them for disease phenotypes with known molecular basis from Online Mendelian Inheritance in Man (OMIM).[66]

Comparisons to published annotations of X-inactivation status

We re-compiled XCI status annotations of individual genes from four studies of Chr X allelic ratios.[14–16,18] Previous XCI status compilations[9] incorporated DNA methylation data, which we excluded because it does not directly measure Xi transcription. Previous compilations also incorporated information about expression in human-rodent hybrid cell lines carrying a human Xi[13]; we incorporated this information only where allelic ratios in human cells were not available. Our final XCI status annotations are listed in Table S6, with the workflow for generating these annotations explained below.

The first dataset that we incorporated was derived from paired genomic and cDNA SNP-chips in skewed LCL and fibroblast cell cultures (Additional file 7 from Cotton et al.).[14] We used the AR values provided (average Xi expression column) for genes informative in at least 5 samples, resulting in AR values for 424 genes. Using the provided numbers of informative samples and standard deviations of AR values, we computed 95% confidence intervals for the AR values. We considered a gene "Subject" to XCI if the AR 95% confidence interval included zero or the AR value was <0.1; otherwise we considered the gene to "Escape".

The second dataset that we incorporated was derived from bulk or single cell RNA-seq of LCLs.[15] The bulk RNA-seq was from an individual in the GTEx dataset with 100% skewed XCI across the body (Table S5 from Tukiainen et al.).[15] The single-cell RNA-seq in LCLs was from three individuals (Table S8 from Tukiainen et al.; we excluded data from one dendritic cell sample).[15] For each dataset, we calculated an AR for each gene using read counts from the more lowly and highly expressed alleles in each sample, and used the provided adjusted p-values to identify genes with significant Xi expression (padj < 0.05). For a gene to be considered informative, we required data from at least two individuals in the single cell dataset, or one individual in the single cell dataset and informative in the bulk RNA-seq dataset, resulting in 82 informative genes. We called a gene as "Subject" to XCI if there was no significant expression from Xi in either the bulk or single-cell datasets, and "Escape" if one or both of the datasets showed evidence of Xi expression.

The third dataset that we incorporated was derived from single-cell allelic expression in fibroblasts.[16] The dataset includes five individuals (Dataset 3 from Garieri et al.)[16] and we required data from at least two samples to be considered informative for a given gene, resulting in 203 genes. We converted their reported values (Xa reads/total reads) to AR values using the following formula: $AR = \frac{1}{\frac{Xa\ reads}{total\ reads}} - 1$. We used the AR threshold calculated in the previous study[16] to consider a gene significantly expressed from Xi in each sample (AR > 0.0526). If a gene had no samples with significant expression from Xi or a mean AR value < 0.1 across samples, we considered it "Subject" to XCI; otherwise, it was judged to "Escape" XCI.

The fourth dataset that we incorporated was derived from allele-specific bulk RNA-seq performed on 136 samples with skewed XCI from the set of GEUVADIS LCLs (Tables S4 and S5 from Sauteraud et al.).[18] For a gene to be considered scorable, we required

that it be informative in at least 10 samples, resulting in 215 genes. We calculated an AR for each gene in each sample using the read counts from the more lowly and highly expressed alleles in each sample, adjusting for the level of skewing in each sample. To identify genes that were significantly expressed from Xi across samples, we performed paired, two-sample, one-sided t tests using the t.test function in R, asking whether the raw (pre-adjusted for skewing) AR values were greater than the baseline AR given the level of skewing in each sample ($baseline\ AR = \frac{1 - skewing\ coefficient}{skewing\ coefficient}$); we corrected the resulting p values for multiple comparisons with the p.adjust function in R using the Benjamini-Hochberg method. Genes were considered "Escape" if they had padj < 0.01, and "Subject" otherwise.

Next, we synthesized the calls from these four datasets. We assigned a gene as "Subject" if: 1) all studies were "Subject" or 2) most (>50%) studies were "Subject" and the average AR across all studies was <0.1. We assigned "Escape" if 1) most (>50%) studies were "Escape" or 2) 50% or fewer (but more than 0) studies were "Escape" and either i) there was more than one study with evidence of escape or ii) the average AR across all studies was ≥ 1. Finally, we assigned "No call" if the gene was not informative in any of the four datasets. For these genes, we investigated whether there were any calls using hybrids from Carrel et al.[13] as compiled in Balaton et al.[9] If a gene had no call in any of the four AR datasets, but had a proportion of expression in Xi hybrids <0.22, we considered the gene "Subject"; genes with a greater proportion were called "Escape."

To compare our calls with previous XCI consensus calls, we made the following modifications to the Balaton list: *XG* had been listed as a PAR gene, but we excluded it from our list of PAR genes because it is located at the PAR boundary and truncated on the Y chromosome. We updated its annotation to escape ("E") since the Balaton table lists evidence for escape. The Balaton table lists *XIST* as "mostly subject" to XCI, but given its exclusive expression from Xi, we updated its status to escape ("E"). We manually examined all genes on our list that were not found in the Balaton list to make sure that genes were not misclassified due to differences in official gene names. For those genes still not present in the Balaton list after this correction, we list "No call". To compare with our annotations, we grouped the Balaton calls into "Escape" if they were annotated as "PAR", "Escape", "Mostly escape", "Variable Escape", "Mostly Variable Escape", or "Discordant". We grouped Balaton calls into "Subject" if they were annotated as "Mostly subject" or "Subject".

We compared our new calls with the Balaton calls for the 423 genes expressed in fibroblasts or LCLs, finding 48 where they differed. Of these, nine had a call in Balaton, but no call in the newer datasets. For two genes (*TCEAL3*, *TMSB4X*), there was no call in Balaton, but newer data enabled a call to be made (both "Subject"). Nineteen genes were called "Subject" in Balaton, but new data indicates that they have expression from Xi and we categorize them as "Escape." The final eighteen genes were called "Escape" in Balaton, but new data suggested they have no expression from Xi. In total, our classification found 86 genes that "Escape", 315 genes that are "Subject" to XCI, and 22 genes with "No call."

Allele-specific expression analysis

This workflow is diagrammed in Figure S13.

SNP calling

We called SNPs in each RNA-seq sample with two X chromosomes (46,XX, 46,XX,+21, 47,XXY, 48,XXYY) following the Broad Institute's "Best Practices" workflow for identifying short variants in RNA-seq data (https://gatk.broadinstitute.org/hc/en-us/articles/360035531192-RNAseq-short-variant-discovery-SNPs-Indels-). To perform our skewing analysis, we filtered for SNPs with the following properties: 1) annotated in the dbSNP database, 2) located in an exon of an expressed gene, 3) displaying a minimum coverage of 10 reads, and 4) heterozygous with at least three reads mapping to each of the reference and alternative alleles. We excluded SNPs where the presence of two alleles likely represented technical artifacts rather than biallelic expression, including in *WASH6P* (SNPs map to multiple near-identical autosomal paralogs), *ATRX* (SNP in a mutation-prone stretch of Ts), and *APOOL* (SNPs within an inverted repeat). For samples with a copy of Chr Y, we excluded SNPs mapping to PAR genes, to avoid measuring allelic contributions of Chr Y.

Identifying cell lines with skewed X chromosome inactivation

We classified genes as "Xa only" (only expressed from Xa) if previously characterized as "silenced" *and* found here to have $\Delta E_X < 0.05$ (FDR > 0.5); see Table S6. We expect that in skewed cell lines, reads from Xa-only genes should be near or completely monoallelic (Figure S12). For each SNP in Xa-only genes, we calculated the "skewing coefficient" by dividing the number of reads from the dominant allele by the total number of reads covering the SNP (Figure S14). These coefficients range from 0.5 (equal expression of two alleles) to 1 (expression from a single allele). For each sample, we computed the median skewing coefficient across all SNPs in Xa-only genes, requiring a threshold of 0.8 to classify as skewed. Using simulations, we find that this level of skewing is unlikely to occur by chance ($P < 1x10^{-6}$), and we do not find evidence of such skewing for SNPs on Chr 8, an autosome with a similar number of expressed genes (Figure S16).

Several samples had few (≤ 5) informative SNPs in Xa-only genes, but many SNPs in other genes (Figure S15). We interpret this to mean that these samples are highly skewed and that we do not observe enough RNA reads covering both alleles to count SNPs in Xa-only genes as informative. Between these highly-skewed samples and the samples with skewing coefficients of at least 0.8, we identified 21 LCLs and 10 fibroblast cultures with skewed XCI.

Determining allelic ratios for X chromosome genes

After identifying the skewed cell lines, we identified genes with informative SNPs values in at least three skewed samples of a given cell type. We then computed the allelic ratio (AR) at each informative SNP by dividing the number of reads from the more lowly

expressed allele by the number of reads from the more highly expressed allele. In cell cultures that are partially skewed, genes will appear more biallelic than in completely skewed cell cultures since there are two populations of cells with different active X chromosomes present – the "major" and "minor" cell populations. Using our skewing estimates, we adjusted the AR on a per-sample basis using the following formula:

$$AR = \frac{AR - AR * t - t}{1 - t - AR * t}$$

Where t is the estimated percentage of cells in the "minor" population (i.e., with the other X chromosome active compared to the "major" cell population), calculated by: 1 – skewing coefficient. For highly-skewed samples, we were unable to calculate a stringent skewing coefficient due to too few SNPs, so we set skewing coefficient = 1. As a result, it is possible that allelic ratios in these samples may be slightly overestimated if the skewing coefficients are in fact <1. Within each sample, we obtained the average AR for each gene by averaging across all informative SNPs in that gene's exons (Figure S17; Table S6) and then calculated the mean AR across skewed samples to obtain a final per-gene AR estimate (Table S6).

To assess whether AR values for each gene were significantly greater than zero, we performed one-sided t tests using the t.test function in R, asking whether the AR values were greater than zero; we corrected the resulting p values for multiple comparisons with the p.adjust function in R using the Benjamini-Hochberg method (Table S6). We also repeated this analysis excluding highly skewed samples, since the skewing coefficients cannot be stringently determined. This removed some informative genes but did not significantly affect the AR values (Figure S19; Table S6).

To identify genes whose AR and ΔE_X values differ significantly, we performed one-sample, two-sided t tests for the AR values across samples, setting mu = ΔE_X (Table S6). We selected genes with Benjamini-Hochberg adjusted p values < 0.1 as having significantly different AR and ΔE_X values. From this list we excluded genes for which the 95% confidence interval of ΔE_X values (1.96*SE) included the mean AR value, and those for which both ΔE_X and AR were not significantly different from zero (FDR $\geq$ 0.05).

We compared our AR values derived from LCLs or fibroblasts with the four published allelic-ratio datasets described in the above methods on generating XCI status calls (Figure S18).

QUANTIFICATION AND STATISTICAL ANALYSES

Various statistical tests were used to calculate p values as indicated in the methods section, figure legend, or text, where appropriate. Results were considered statistically significant when $p < 0.05$ or FDR<0.05 when multiple hypothesis correction was applied, unless stated otherwise. Data are shown as median and interquartile range, unless stated otherwise. All statistics were calculated using R software, version 3.6.3.[67]

Cell Genomics, Volume *3*

Supplemental information

The human inactive X chromosome modulates expression of the active X chromosome

Adrianna K. San Roman, Alexander K. Godfrey, Helen Skaletsky, Daniel W. Bellott, Abigail F. Groff, Hannah L. Harris, Laura V. Blanton, Jennifer F. Hughes, Laura Brown, Sidaly Phou, Ashley Buscetta, Paul Kruszka, Nicole Banks, Amalia Dutra, Evgenia Pak, Patricia C. Lasutschinkow, Colleen Keen, Shanlee M. Davis, Nicole R. Tartaglia, Carole Samango-Sprouse, Maximilian Muenke, and David C. Page

Supplemental Information

Figures S1-S19

Figure S1

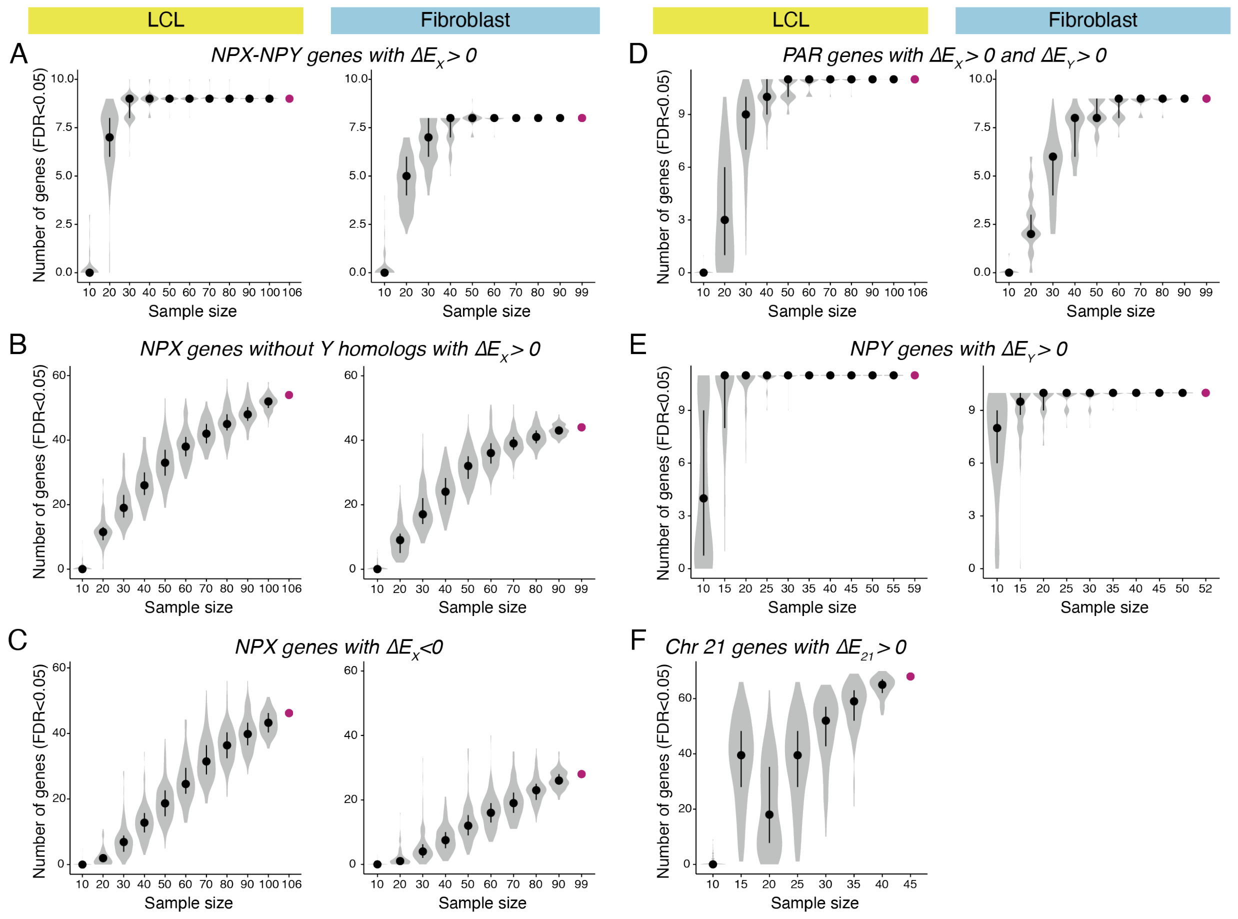

Figure S1. Bootstrapping analyses reveal that few additional significant genes would be observed with a larger sample size, related to Figures 2 and 3. Each analysis was performed by randomly choosing the indicated number of samples and performing the linear regression analysis, repeated 100 times for each sample size. The magenta point in each figure represents the number of significant genes in the final analysis using all of the samples. **(A)** NPX genes with Y homologs with $\Delta E_X>0$ reach saturation by a sample size of 40. (**B)** For NPX genes without Y homologs, the number of genes with $\Delta E_X>0$ increases rapidly at low sample sizes, and then levels off. **(C)** For NPX genes with $\Delta E_X<0$, more samples are required to see an initial increase in the number of significant genes. In LCLs, 50-60 samples are needed to identify 20 genes with $\Delta E_X<0$, whereas only 30-40 samples are needed to identify 20 genes with $\Delta E_X>0$. For LCLs, the maximum increase was observed between 60-70 samples where a median of 7 genes were added, with subsequent increases being less (5 genes from 70-80, 3.5 from 80-90 and 3.5 from 90-100). In fibroblasts, the maximum increase was observed between 40-50 samples where

a median of 4.5 genes were added, with only 3 or 4 additional genes with each additional 10 samples. If this trajectory continues, these results indicate that additional samples would only result in a few additional genes being identified as significant. **(D)** PAR1 genes with both $\Delta E_X>0$ and $\Delta E_Y>0$ reach saturation by sample size of 50. **(E)** NPY genes with $\Delta E_Y>0$ reach saturation after 25 samples containing at least one copy of Chr Y. **(F)** The trajectory for Chr 21 genes with $\Delta E_{21}>0$ increases rapidly between 10-30 samples, and then slows.

Figure S2.

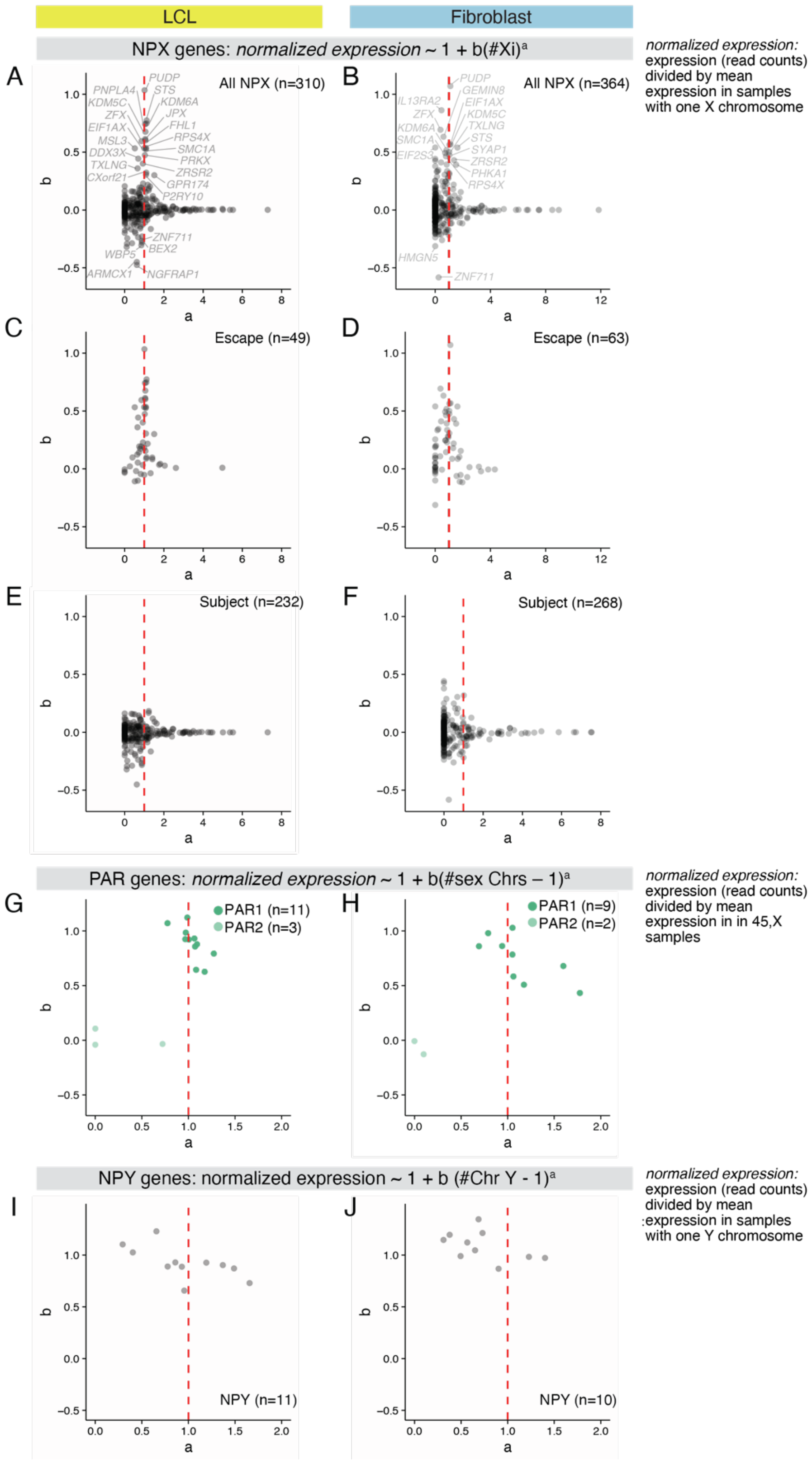
LCL
Fibroblast
NPX genes: normalized expression ~ 1 + b(#Xi)a
normalized expression: expression (read counts) divided by mean expression in samples with one X chromosome
A
All NPX (n=310)
PUDP
PNPLA4
STS
KDM5C
KDM6A
ZFX
JPX
EIF1AX
FHL1
MSL3
RPS4X
DDX3X
SMC1A
PRKX
TXLNG
ZRSR2
CXorf21
GPR174
P2RY10
ZNF711
BEX2
WBP5
ARMCX1
NGFRAP1
B
All NPX (n=364)
PUDP
GEMIN8
IL13RA2
EIF1AX
ZFX
KDM5C
KDM6A
TXLNG
SMC1A
STS
EIF2S3
SYAP1
ZRSR2
PHKA1
RPS4X
HMGN5
ZNF711
C
Escape (n=49)
D
Escape (n=63)
E
Subject (n=232)
F
Subject (n=268)
PAR genes: normalized expression ~ 1 + b(#sex Chrs – 1)a
normalized expression: expression (read counts) divided by mean expression in in 45,X samples
G
PAR1 (n=11)
PAR2 (n=3)
H
PAR1 (n=9)
PAR2 (n=2)
NPY genes: normalized expression ~ 1 + b (#Chr Y - 1)a
normalized expression: expression (read counts) divided by mean :expression in samples with one Y chromosome
I
NPY (n=11)
J
NPY (n=10)
a
b

Figure S2. Linear functions are a good fit for modeling sex chromosome aneuploidy RNA-seq data, related to Figures 2 and 3. For sex chromosome genes in LCLs and fibroblasts, power functions were fit (gray boxes) by non-linear least squares regression. Scatterplots show fitted values for the exponent parameter, *a*, and the coefficient, *b*, for each gene. *XIST* and genes for which the regression model did not converge are excluded from the plots. **(A-B)** Some NPX genes cluster near *a*=1, indicating their expression increases by a fixed amount for each additional X or Y chromosome. These correspond to genes previously annotated as escaping XCI **(C-D)**. Other NPX genes cluster near *a*=0 or *b*=0, indicating that they do not change in expression with additional copies of Xi, and correspond to genes previously annotated as subject to XCI (**E-F**). (**G-H**) PAR1 genes cluster near a=1, indicating their expression increases by a fixed amount for each additional sex chromosome, in contrast to PAR2 genes. (**I-J**) NPY genes also cluster near a=1, indicating their expression increases by a fixed amount for each Y chromosome.

Figure S3.

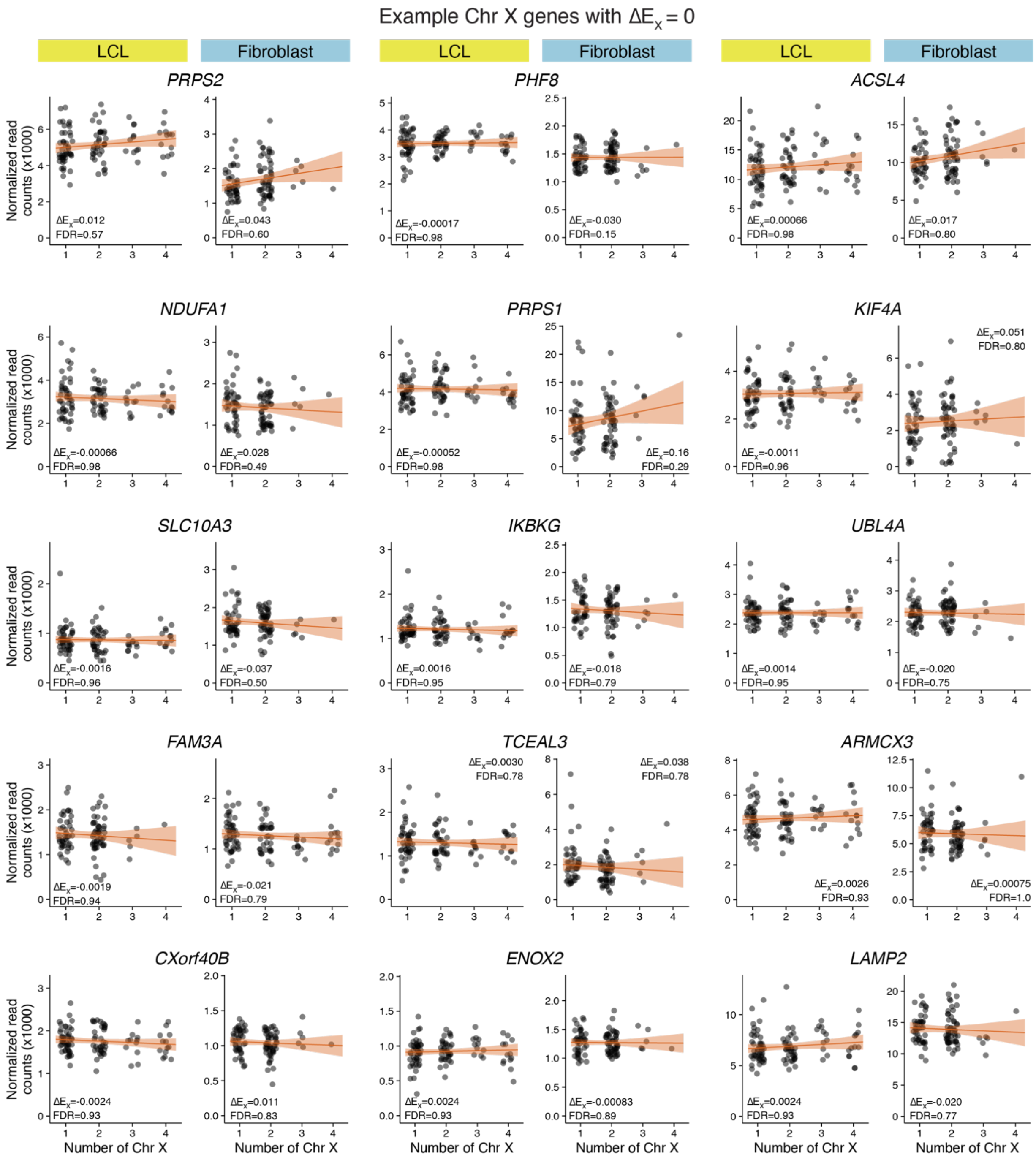

Figure S3. Extended examples of NPX genes with ΔE_X=0, related to Figure 2. Scatterplots and regression lines with confidence intervals for fifteen NPX genes with ΔE_X=0 in both LCLs and fibroblasts.

Figure S4.

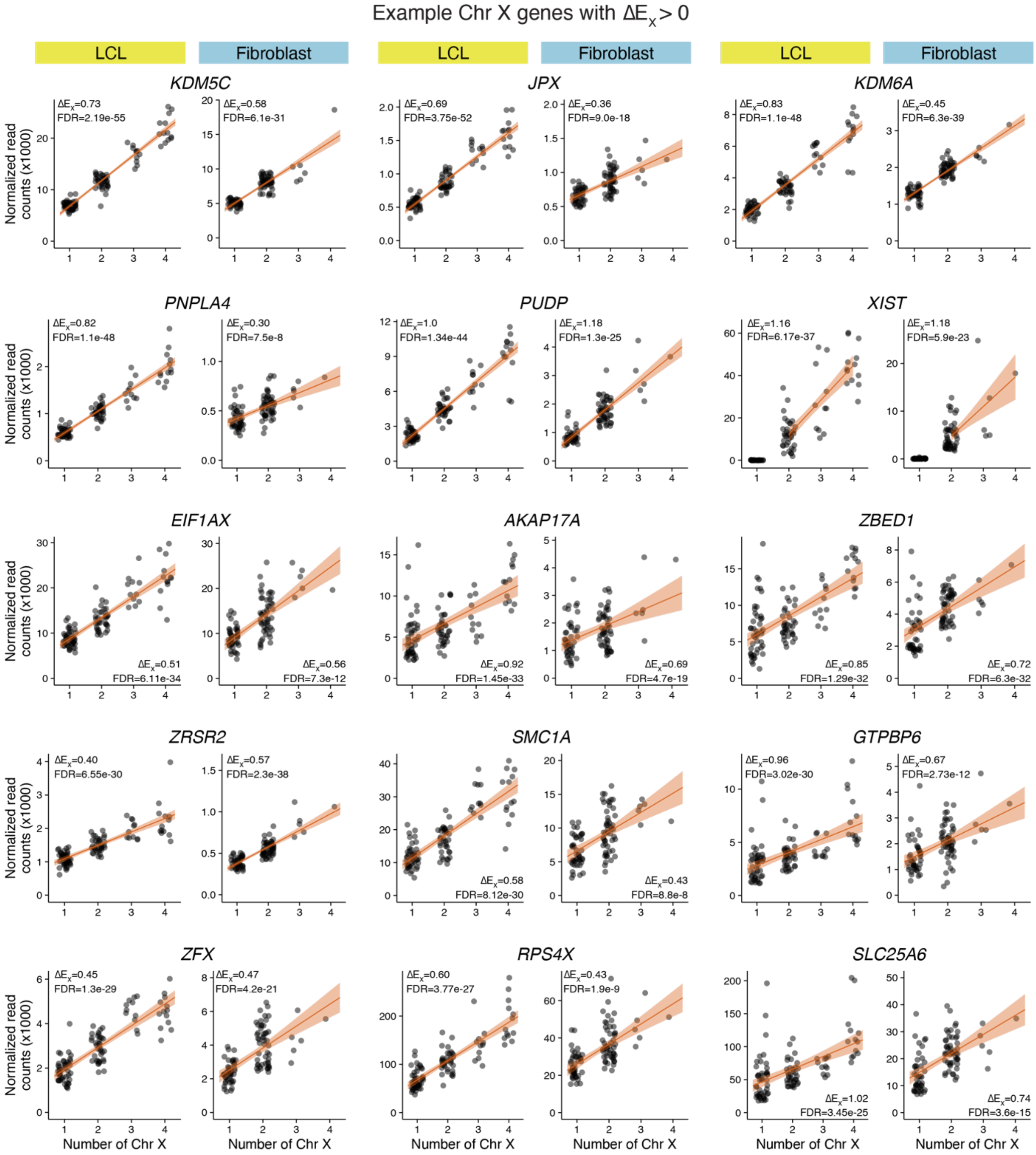

Figure S4. Extended examples of NPX genes with ΔE$_X$>0, related to Figure 2. Scatterplots and regression lines with confidence intervals for fifteen NPX genes with ΔE$_X$>0 in both LCLs and fibroblasts, ordered by increasing FDR in LCLs.

Figure S5.

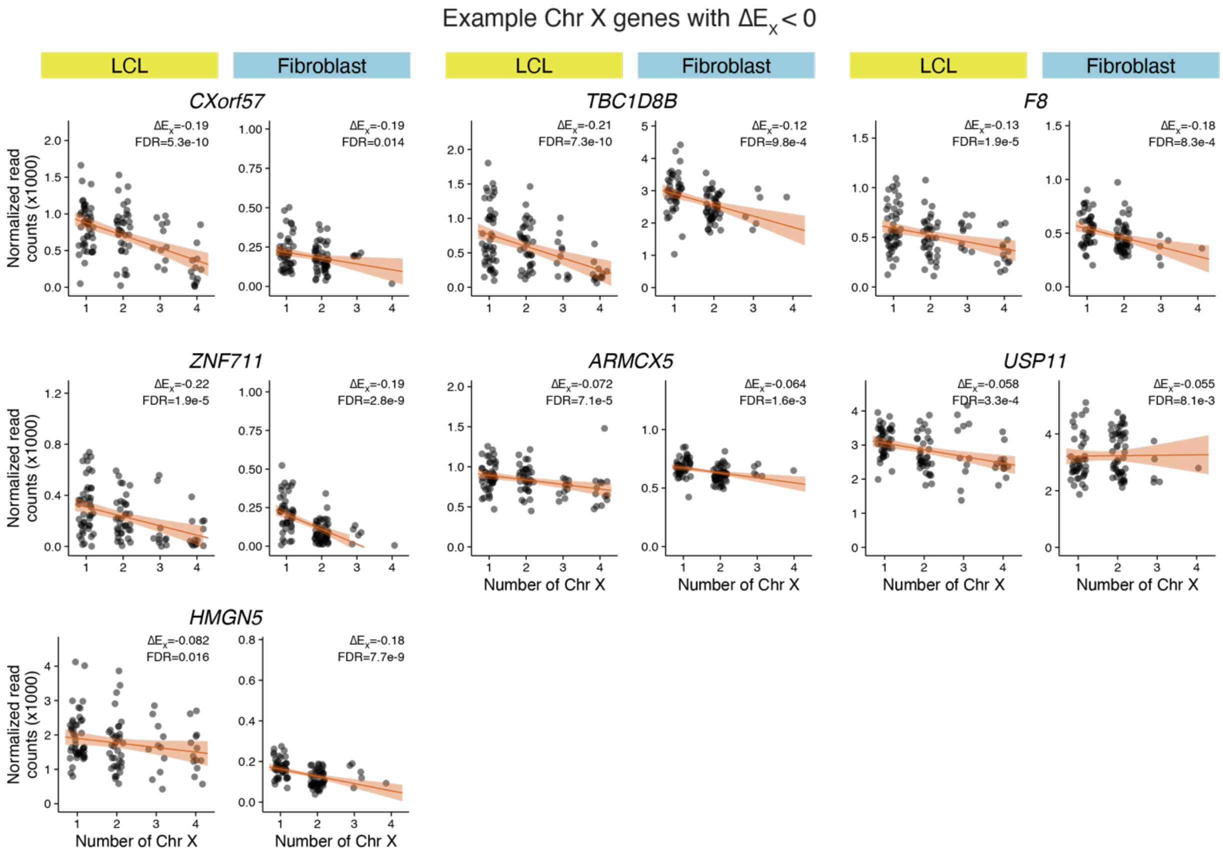

Figure S5. Extended examples of NPX genes with ΔEX<0, related to Figure 2. Scatterplots and regression lines with confidence intervals for seven NPX genes with ΔEX<0 in both LCLs and fibroblasts, ordered by increasing FDR in LCLs.

Figure S6.

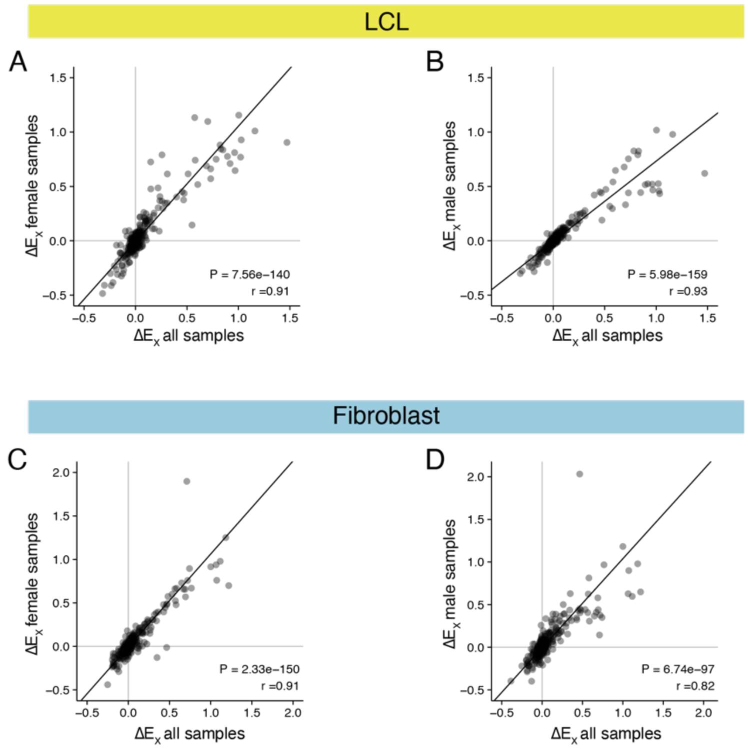

Figure S6. ΔE_X values are similar when calculated with all samples, or with female (0 Y chromosomes) or male (1 Y chromosome) samples, related to Figure 2. Scatterplots of all expressed genes in LCLs (A-B) or fibroblasts (C-D) showing ΔE_X values calculated using all samples (X-axes) or exclusively female samples (A, C Y-axes) or male samples (B, D Y-axes). Deming regression lines, Pearson correlations and P-values are indicated.

Figure S7.

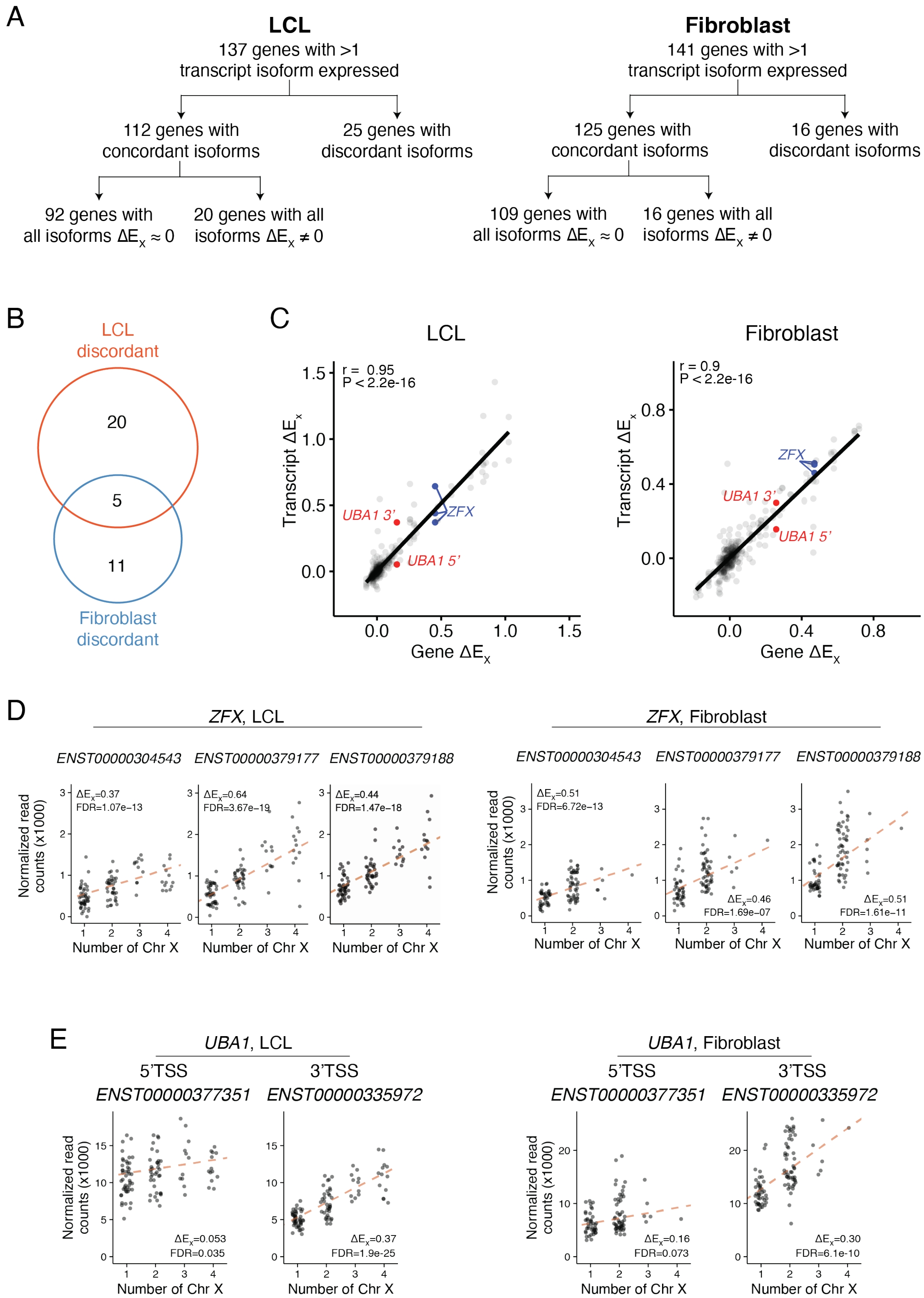

Figure S7. Quantitative analysis of Xi contributions to transcript isoform expression, related to Figure 2. (A) Accounting of Chr X genes with multiple expressed transcript isoforms in LCLs and fibroblasts. Indicated here are the numbers of genes where i) all isoforms show

$\Delta E_X \approx 0$, or ii) all isoforms show ΔE_X values significantly different from zero in the same direction (FDR<0.05), or iii) isoforms show discordant ΔE_X values. For genes with discordant isoforms, two in LCLs (*ARHGEF6, BCOR*) and three in fibroblasts (*SH3KBP1, THOC2, UBA1*) had TSSs >500bp from each other suggesting that differential regulation of the TSS could play a role. **(B)** Venn diagram shows the overlap of genes expressed in both LCL and fibroblasts that had isoforms with discordant ΔE_X values. **(C)** Scatterplots of each transcript isoform's ΔE_X, compared with the gene's ΔE_X in LCLs and fibroblasts. Three *ZFX* isoforms are indicated with blue points; two *UBA1* isoforms indicated with red points. Pearson correlations (r) between isoforms and genes and P-values are indicated. **(D)** Scatterplots of expression of three *ZFX* isoforms across Chr X copy number in LCLs and fibroblasts showing that they consistently have $\Delta E_X > 0$. **(E)** Scatterplots of expression of two *UBA1* isoforms across Chr X copy number in LCLs and fibroblasts showing discordant ΔE_X values. Regression lines, ΔE_X, and FDR are indicated.

Figure S8.

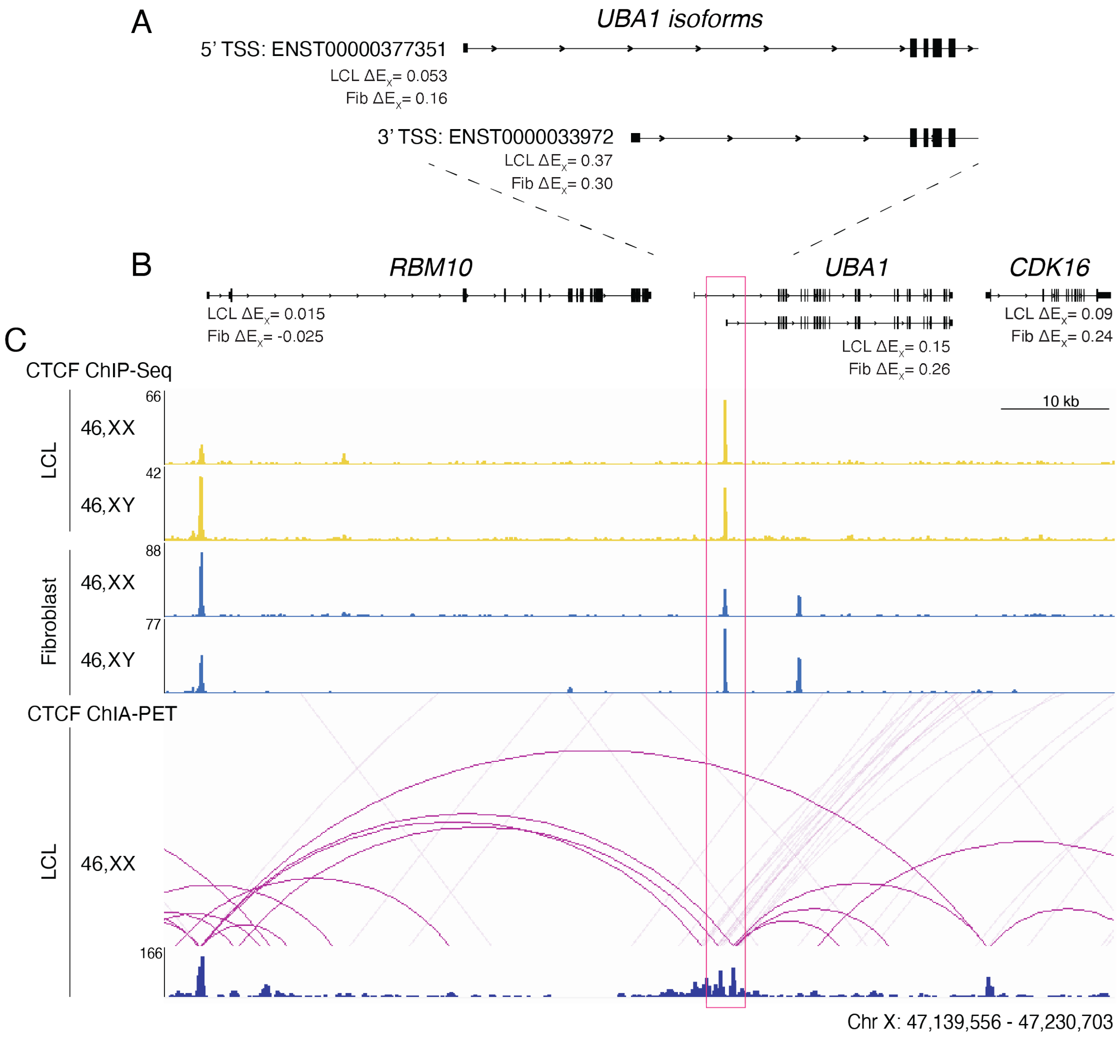

Figure S8. Divergent ΔE_X values for transcript isoforms of *UBA1* correlate with domain boundary, related to Figure 2. (A) Transcript models for *UBA1* transcript isoforms. Isoform-level ΔE_X values are listed. **(B)** Gene models for *UBA1* and flanking genes *RBM10* and *CDK16.* Gene-level ΔE_X values are listed. ΔE_X values for these flanking genes are discordant and reflect the ΔE_X values of the closest *UBA1* isoform. *UBA1* isoform ΔE_X values correlate with those of the flanking genes: the *UBA1* transcript from the 5' start (ENST00000377351) and upstream gene *RBM10* had ΔE_X values near zero, while the *UBA1* transcript from the 3' start (ENST0000033972) and downstream gene *CDK16* had significantly positive ΔE_X values. **(C)** CTCF ChIP-seq signal tracks (fold-change over background) for two LCL and two fibroblast cell lines show a strong CTCF peak between the two *UBA1* isoform transcription start sites and evidence of divergent three-dimensional interactions, indicating that the two transcription start

sites reside in distinct topologically associated domains along with their neighboring genes. Interaction and signal tracks (fold-change over background) are shown for CTCF ChIA-PET. Red box indicates boundary region. Three previous studies corroborate these results: 1) in human-mouse hybrid cell lines, the 5' TSS was only active in lines containing Xa, while the 3' TSS was active in lines containing Xa or Xi [S1]; 2) 3' TSS transcripts were more abundant in 46,XX than 46,XY cells, while 5' TSS transcripts showed no such difference [S2] and 3) DNA methylation studies revealed hypermethylation of the 5' but not the 3' TSS in XX cells from human, chimp, and horse [S3]. Together, this publicly available data and our own analysis shows that *UBA1* is a unique example of a gene with transcription start sites that are divergently expressed from Xi.

Figure S9.

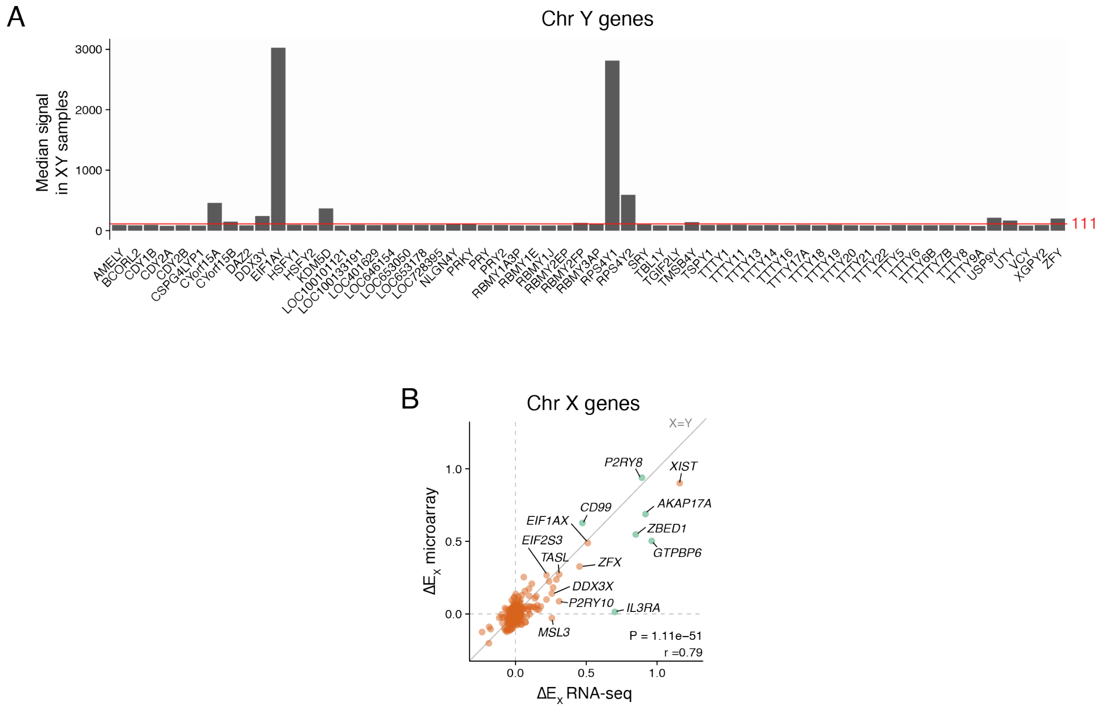

Figure S9. Reanalysis of microarray data from LCLs of individuals with varying sex chromosome constitutions using linear modeling, related to Figure 2. (A) Chr Y gene expression in microarray data from XY samples [S4] was assessed to set a minimum signal threshold of 111 (red line) for expressed genes, based on those expressed in LCL RNA-seq. **(B)** ΔE_X values in RNA-seq and microarray data for expressed Chr X genes were highly correlated. Deming regression line, Pearson correlation and P-value are indicated.

Figure S10.

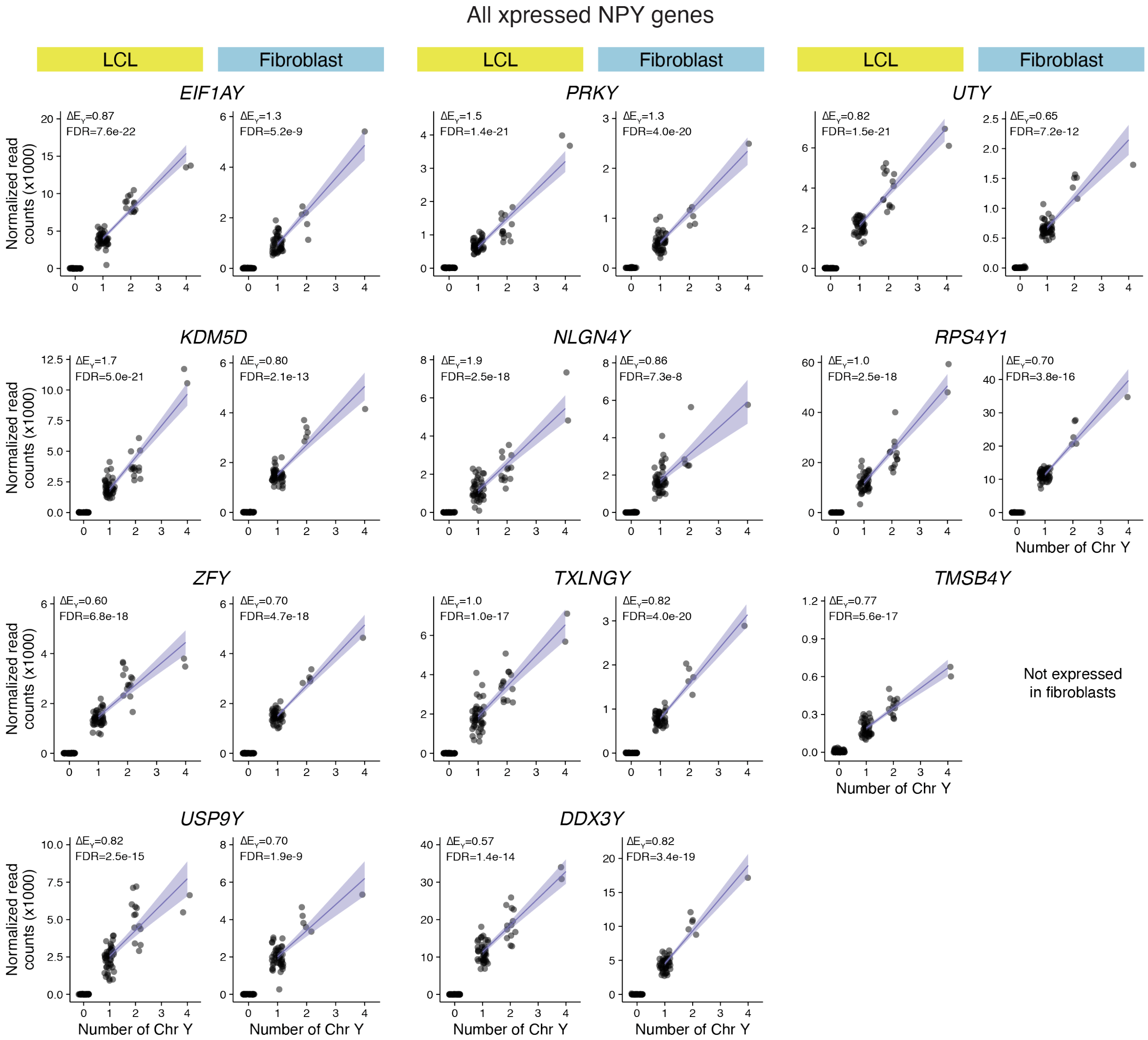

Figure S10. Extended plots of NPY genes, related to Figure 3. Scatterplots and regression lines with confidence intervals for the twelve expressed NPY genes in LCLs or fibroblasts, ordered by increasing FDR in LCLs.

Figure S11.

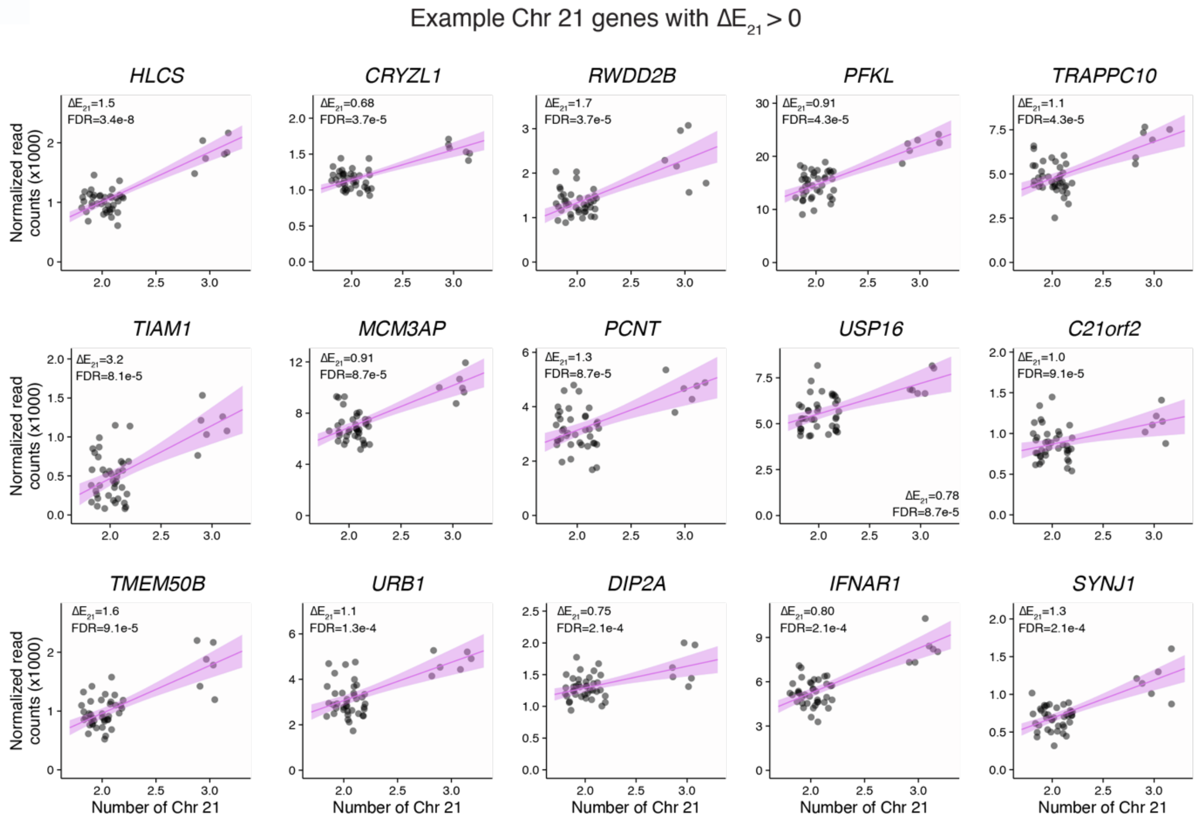

Figure S11. Extended examples of Chr 21 genes with ΔE_{21}>0, related to Figure 3.

Scatterplots and regression lines with confidence intervals for fifteen expressed Chr 21 genes with ΔE_{21}>0 in LCLs, ordered by increasing FDR.

Figure S12.

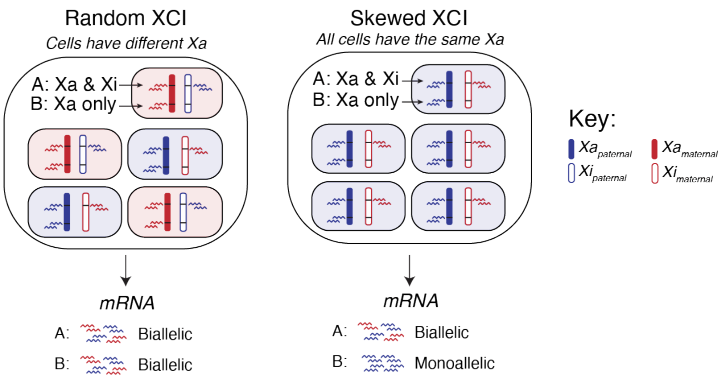

Figure S12. Identifying cell cultures with skewed XCI from RNA-seq data, related to Figure 4. 46,XX individuals typically have a mixture of cells in which either the maternal or paternal X chromosome is active. Because of this, in mRNA extracted from cell cultures with random XCI, all Chr X genes will have biallelic transcripts (*left*). On the other hand, in cell cultures with skewed XCI – where most cells in the culture have the same Xa – genes expressed only from Xa will have monoallelic transcripts, while genes expressed from both Xa and Xi will have biallelic transcripts (*right*).

Figure S13.

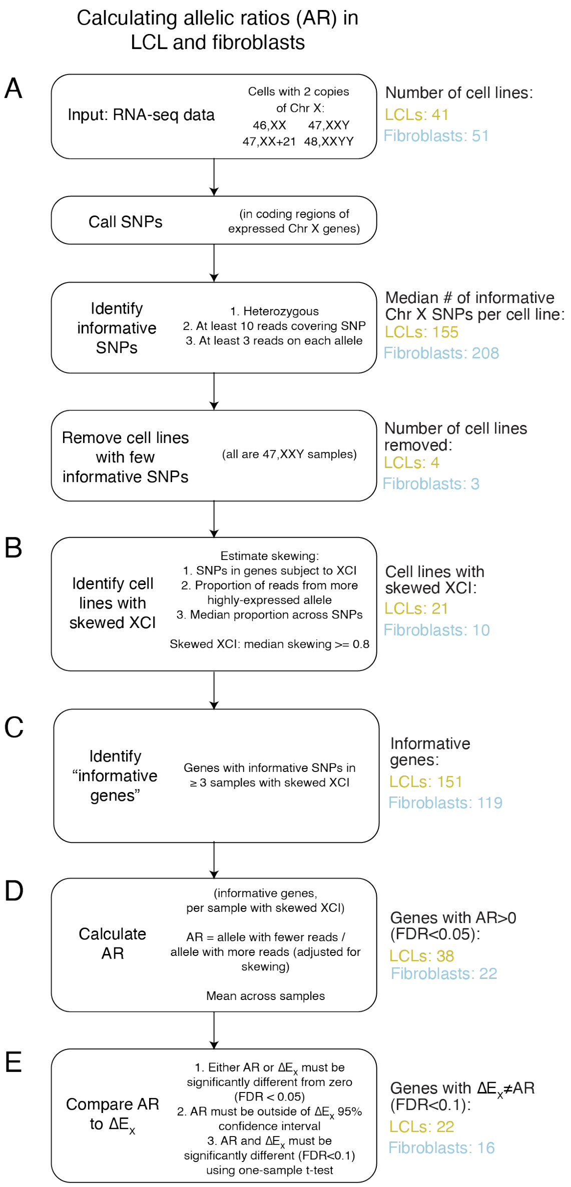

Figure S13. Analytic workflow for identifying cell lines with skewed XCI and calculating allelic ratios from RNA-seq data , related to Figure 4. Each box indicates a step in the analytical pipeline; to the right of each box are summaries of results at each step. **(A)** SNPs were called in samples with two copies of Chr X (46,XX, 46,XX,+21, 47,XXY, 48,XXYY) to identify those that were heterozygous, and could therefore distinguish between two alleles. Of the 41 LCL and 51 fibroblast samples with two copies of Chr X, 7 samples (4 LCLs and 3 fibroblasts; all 47,XXY) were removed because they had few heterozygous SNPs identified on Chr X, indicating that they have two copies of the same Chr X, most likely inherited through a maternal meiotic non-disjunction. **(B)** For samples with sufficient heterozygous Chr X SNPS, the

proportion of reads from the more highly-expressed allele in SNPs from "Xa only" genes ("skewing coefficient") was calculated. This value can range from 0.5 (equal reads from two alleles) to 1 (all reads are from one allele). Eleven LCL samples and nine fibroblast samples with at least 0.8 median skewing coefficients were considered skewed. Some samples (10 LCL samples and one fibroblast culture) had ≤5 SNPs in "Xa only" genes, but many SNPs in other Chr X genes, indicating that they were "highly skewed." Together, 21 LCL and 10 fibroblast samples had skewed XCI. **(C)** Following identification of samples with skewed XCI, "informative genes" were classified as those having informative SNPs in at least 3 samples with skewed XCI, resulting in 151 genes in LCLs and 119 in fibroblasts. **(D)** Next, the allelic ratio (AR) was calculated for each SNP by dividing the allele with fewer reads (assumed to represent Xi) by the allele with more reads (assumed to represent Xa), adjusting for the level of skewing in each sample using the median skewing estimate. **(E)** Finally, AR values were compared to ΔE_X values to identify genes that, considering their variance in each metric, were significantly non-equal, and indicated that their expression from Xa was modulated by additional copies of Xi.

Figure S14.

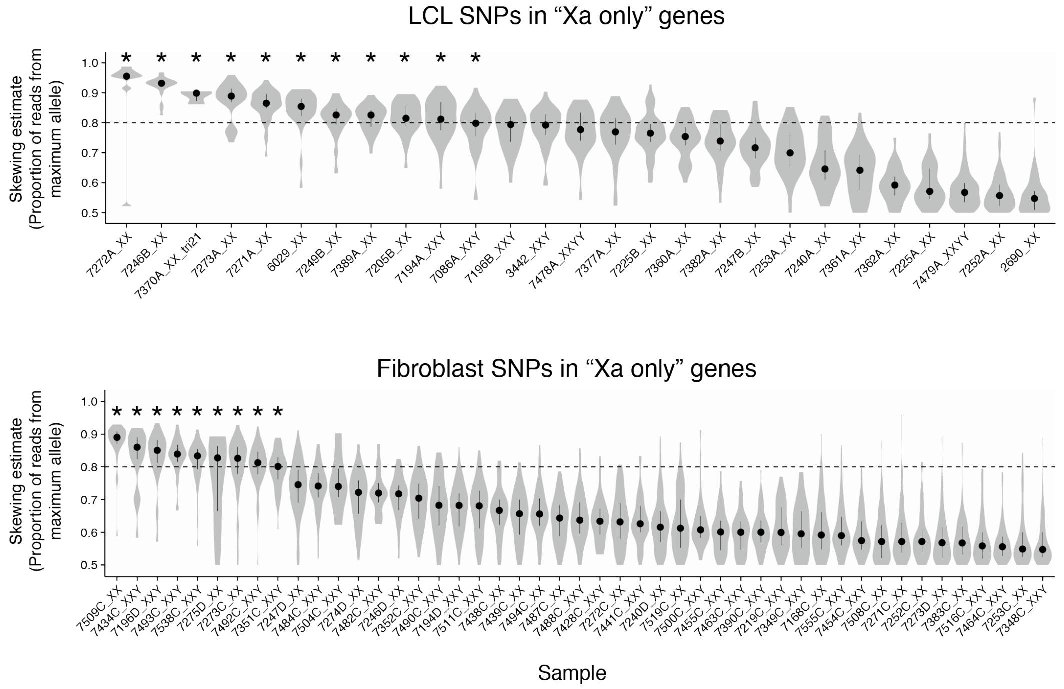

Figure S14. Samples with skewed XCI identified through SNP analysis of Chr X genes expressed from Xa only, related to Figure 4. Samples with >5 heterozygous SNPs in “Xa only” genes were ranked by the median skewing estimate across SNPs in “Xa only” genes. Asterisks, samples with skewing estimates ≥ 0.8 indicating significant skewing of XCI.

Figure S15.

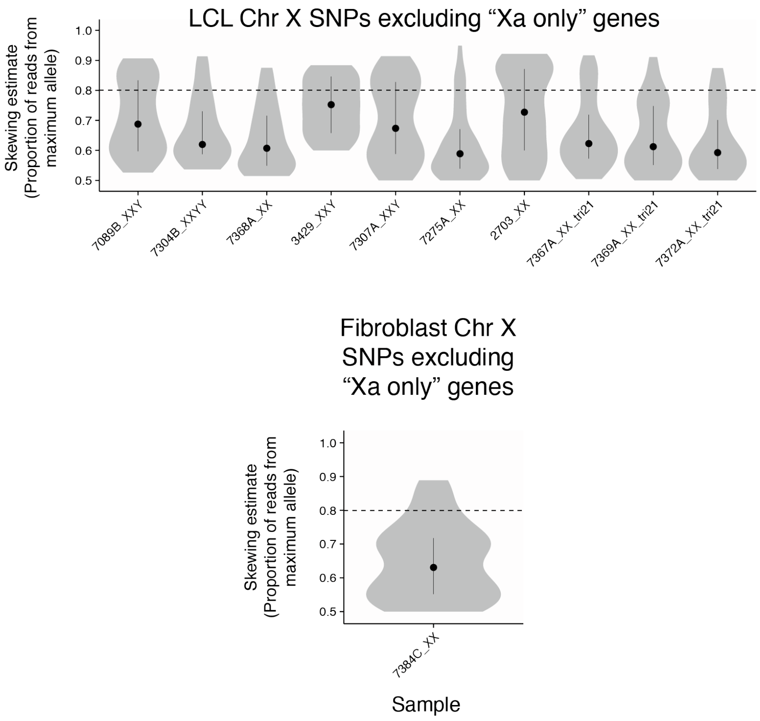

Figure S15. Presence of informative SNPs in Chr X genes not on the "Xa only" list distinguish "highly skewed" samples from those with identical copies of Chr X, related to Figure 4. Samples with ≤ 5 SNPs in "Xa only" genes have informative SNPs in other Chr X genes. This indicates that these X chromosomes are not identical, but that they are so highly skewed that informative SNPs are not identified in "Xa only" genes (due to the requirement for at least 3 reads mapping to the reference and alternative alleles).

Figure S16.

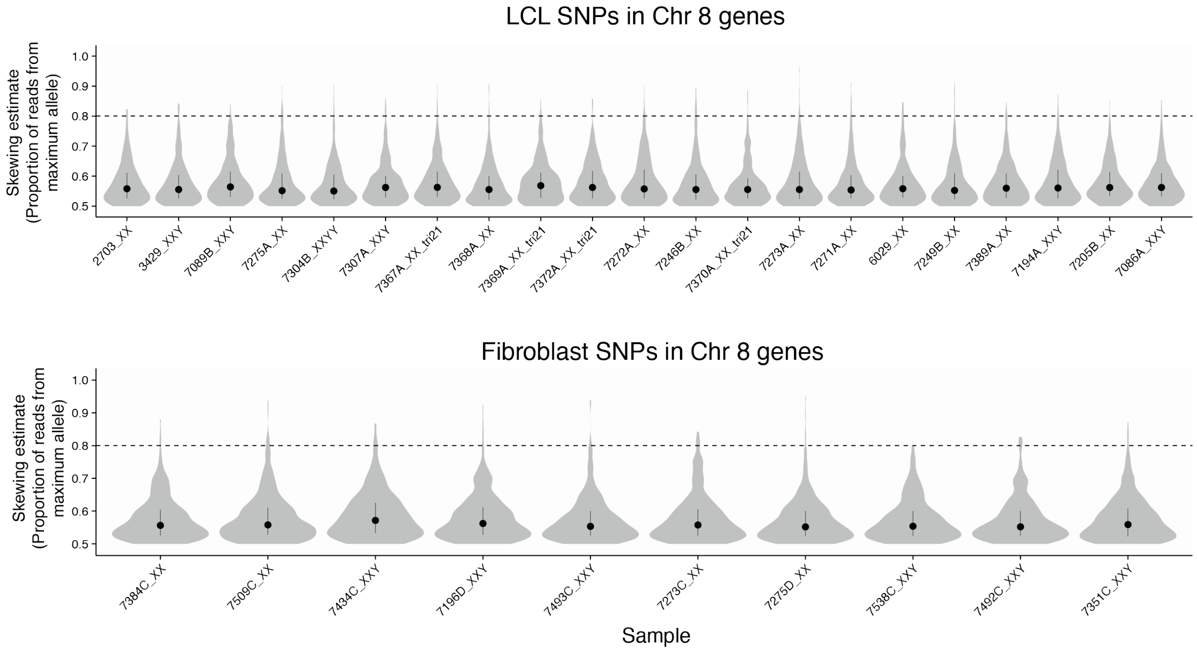

Figure S16. SNPs in Chr 8 genes do not show skewing, indicating that skewing is specific to Chr X and not an artifact observed genome-wide, related to Figure 4. Skewing analysis was performed using SNPs in expressed genes on Chr 8 on samples with skewed XCI. Note uniform skewing estimates of <0.6 across LCL and fibroblast samples, indicating relatively equal expression of each allele.

Figure S17.

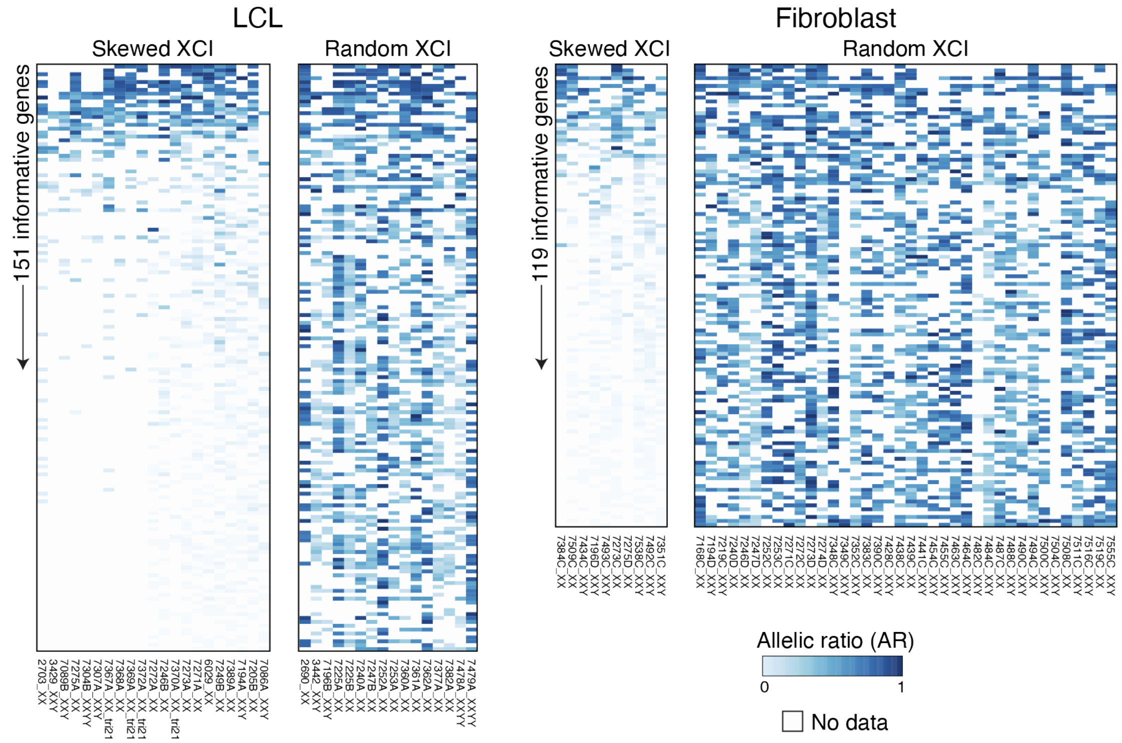

Figure S17. Genes with high and low allelic ratios can be distinguished in samples with skewed XCI, but not in samples with random XCI, related to Figure 4.

Heatmaps showing adjusted allelic ratio data for informative genes (those with data in at least 3 samples with skewed XCI) in LCLs or fibroblasts. Genes (rows) are ordered by the average adjusted AR across samples with skewed XCI. Raw AR values for samples with random XCI (those that did not reach the 0.8 median skewing threshold) are shown for comparison.

Figure S18.

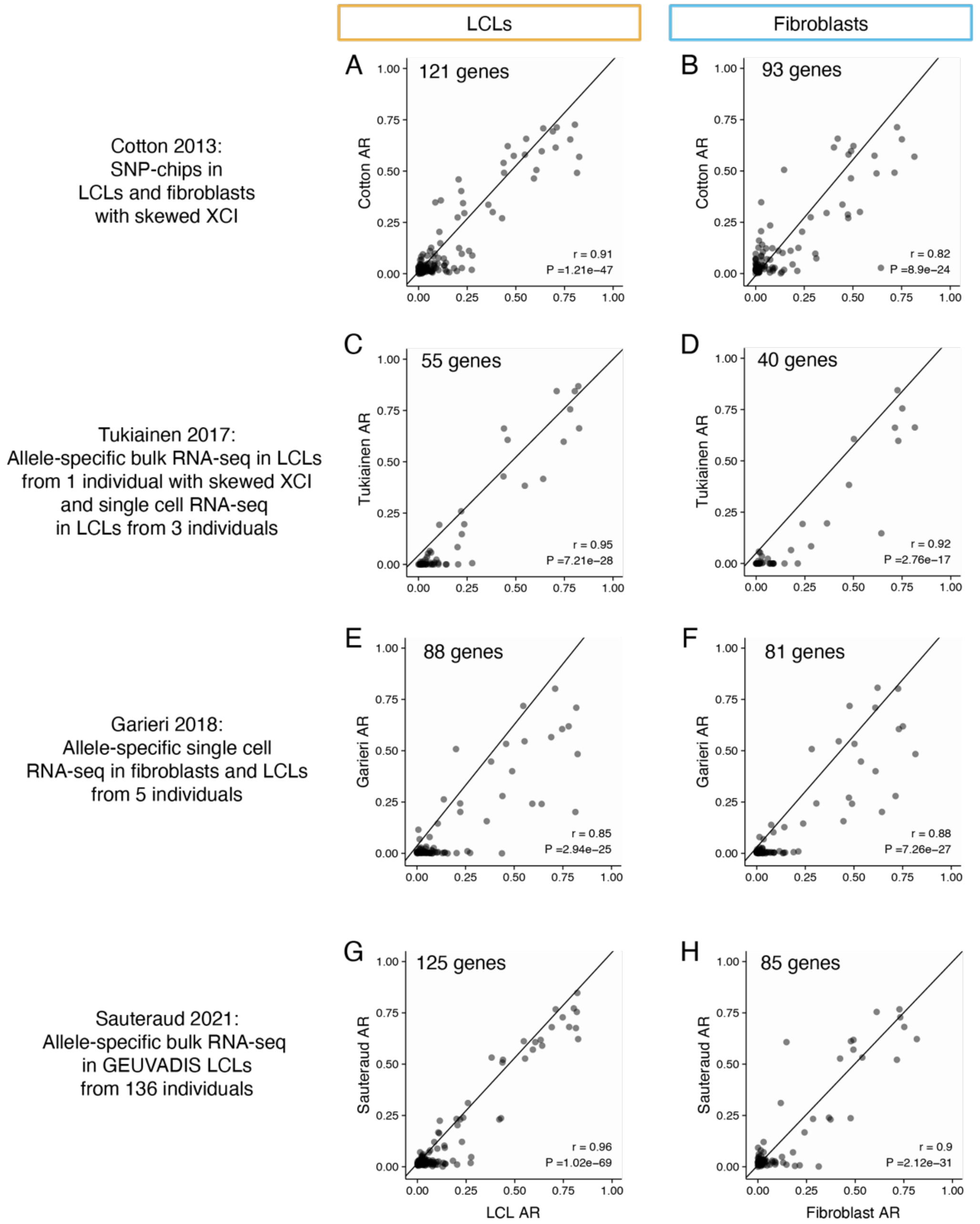

Figure S18. Allelic ratios calculated in this study are correlated with published allelic ratios, related to Figure 4. Scatterplots of the AR values calculated in this study versus AR values from published studies [S5-8]. Genes that were informative in both studies are included on the plots. Deming regression lines, Pearson correlation and P-values are indicated.

Figure S19.

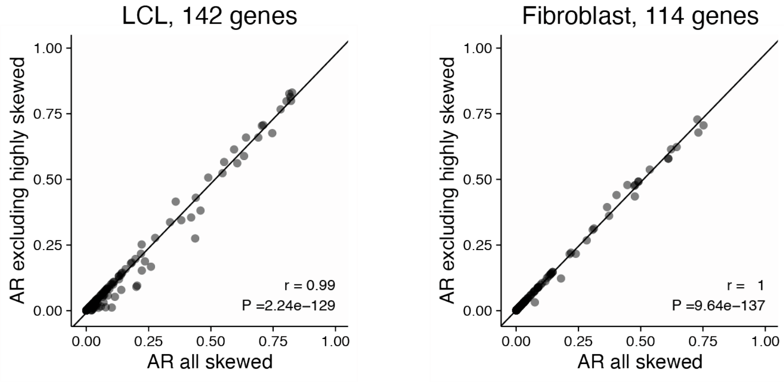

Figure S19. Including highly-skewed samples does not result in overestimation of allelic ratios, related to Figure 4. Scatterplots comparing AR calculated using all skewed samples, or without the "highly skewed" samples. Deming regression lines, Pearson correlation and P-values are indicated.

References

[S1] Goto, Y., and Kimura, H. (2009). Inactive X chromosome-specific histone H3 modifications and CpG hypomethylation flank a chromatin boundary between an X-inactivated and an escape gene. Nucleic Acids Res. *37*, 7416-7428. 10.1093/nar/gkp860.

[S2] Chen, C.Y., Shi, W., Balaton, B.P., Matthews, A.M., Li, Y., Arenillas, D.J., Mathelier, A., Itoh, M., Kawaji, H., Lassmann, T., et al. (2016). YY1 binding association with sex-biased transcription revealed through X-linked transcript levels and allelic binding analyses. Sci. Rep. *6*, 37324. 10.1038/srep37324.

[S3] Balaton, B.P., Fornes, O., Wasserman, W.W., and Brown, C.J. (2021). Cross-species examination of X-chromosome inactivation highlights domains of escape from silencing. Epigenetics Chromatin *14*, 12. 10.1186/s13072-021-00386-8.

[S4] Raznahan, A., Parikshak, N.N., Chandran, V., Blumenthal, J.D., Clasen, L.S., Alexander-Bloch, A.F., Zinn, A.R., Wangsa, D., Wise, J., Murphy, D.G.M., et al. (2018). Sex-chromosome dosage effects on gene expression in humans. Proc. Natl. Acad. Sci. USA *115*, 7398-7403. 10.1073/pnas.1802889115.

[S5] Cotton, A.M., Ge, B., Light, N., Adoue, V., Pastinen, T., and Brown, C.J. (2013). Analysis of expressed SNPs identifies variable extents of expression from the human inactive X chromosome. Genome Biol. *14*, R122. 10.1186/gb-2013-14-11-r122.

[S6] Tukiainen, T., Villani, A.C., Yen, A., Rivas, M.A., Marshall, J.L., Satija, R., Aguirre, M., Gauthier, L., Fleharty, M., Kirby, A., et al. (2017). Landscape of X chromosome inactivation across human tissues. Nature *550*, 244-248. 10.1038/nature24265.

[S7] Garieri, M., Stamoulis, G., Blanc, X., Falconnet, E., Ribaux, P., Borel, C., Santoni, F., and Antonarakis, S.E. (2018). Extensive cellular heterogeneity of X inactivation revealed by single-cell allele-specific expression in human fibroblasts. Proc. Natl. Acad. Sci. USA *115*, 13015-13020. 10.1073/pnas.1806811115.

[S8] Sauteraud, R., Stahl, J.M., James, J., Englebright, M., Chen, F., Zhan, X., Carrel, L., and Liu, D.J. (2021). Inferring genes that escape X-Chromosome inactivation reveals important contribution of variable escape genes to sex-biased diseases. Genome Res. *31*, 1629-1637. 10.1101/gr.275677.121.
